# Defining biologically relevant and hierarchically nested population units to inform wildlife management

**DOI:** 10.1002/ece3.9565

**Published:** 2022-11-30

**Authors:** Michael S. O'Donnell, David R. Edmunds, Cameron L. Aldridge, Julie A. Heinrichs, Adrian P. Monroe, Peter S. Coates, Brian G. Prochazka, Steve E. Hanser, Lief A. Wiechman

**Affiliations:** ^1^ U.S. Geological Survey Fort Collins Science Center Fort Collins Colorado USA; ^2^ Natural Resource Ecology Laboratory, U.S. Geological Survey, Fort Collins Science Center Colorado State University Fort Collins Colorado USA; ^3^ U.S. Geological Survey Western Ecological Research Center Dixon California USA; ^4^ U.S. Geological Survey Ecosystems Mission Area Fort Collins Colorado USA

**Keywords:** *Centrocercus urophasianus*, clustering, greater sage‐grouse, hierarchical population management units, population structure, sagebrush biome

## Abstract

Wildlife populations are increasingly affected by natural and anthropogenic changes that negatively alter biotic and abiotic processes at multiple spatiotemporal scales and therefore require increased wildlife management and conservation efforts. However, wildlife management boundaries frequently lack biological context and mechanisms to assess demographic data across the multiple spatiotemporal scales influencing populations. To address these limitations, we developed a novel approach to define biologically relevant subpopulations of hierarchically nested population levels that could facilitate managing and conserving wildlife populations and habitats. Our approach relied on the Spatial “K”luster Analysis by Tree Edge Removal clustering algorithm, which we applied in an agglomerative manner (bottom‐to‐top). We modified the clustering algorithm using a workflow and population structure tiers from least‐cost paths, which captured biological inferences of habitat conditions (functional connectivity), dispersal capabilities (potential connectivity), genetic information, and functional processes affecting movements. The approach uniquely included context of habitat resources (biotic and abiotic) summarized at multiple spatial scales surrounding locations with breeding site fidelity and constraint‐based rules (number of sites grouped and population structure tiers). We applied our approach to greater sage‐grouse (*Centrocercus urophasianus*), a species of conservation concern, across their range within the western United States. This case study produced 13 hierarchically nested population levels (akin to cluster levels, each representing a collection of subpopulations of an increasing number of breeding sites). These closely approximated population closure at finer ecological scales (smaller subpopulation extents with fewer breeding sites; cluster levels ≥2), where >92% of individual sage‐grouse's time occurred within their home cluster. With available population monitoring data, our approaches can support the investigation of factors affecting population dynamics at multiple scales and assist managers with making informed, targeted, and cost‐effective decisions within an adaptive management framework. Importantly, our approach provides the flexibility of including species‐relevant context, thereby supporting other wildlife characterized by site fidelity.

## Graphical abstract

There is a long‐recognized need to consider population dynamics and outcomes at multiple spatial scales for improving management. Therefore, we developed a novel approach to define biologically relevant subpopulations of hierarchically nested population‐levels that could facilitate the management and conservation of wildlife populations and habitats. The approach can be used to distinguish population dynamics driven by processes occurring at fine scales (small cluster levels) to processes operating at larger scales, such as population cycling.

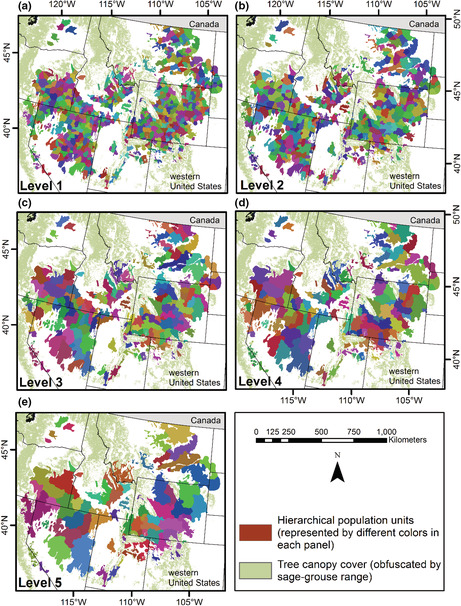

## 1 INTRODUCTION

Wildlife management boundaries frequently reflect a single‐scaled jurisdictional demarcation without considering ecological processes affecting wildlife (e.g., habitat and wildlife movements; Meisingset et al., 2018; Schultz et al., 2019). Changes to environmental conditions and factors affecting wildlife demographics occur across jurisdictional boundaries that are affected by processes operating at multiple scales. Defining boundaries that reflect a species' population structure (i.e., genetic or spatial configuration of population distributions and movements) can better align management to factors that influence demographic rates, such as changes in habitat conditions, predator–prey dynamics, ecosystem processes (e.g., climate and available habitat resources), disease, and management practices (e.g., hunting; Jackson & Fahrig, 2012). Species' responses to environmental variation are frequently scale‐dependent (i.e., scale of effect), but managing populations while considering scale has eluded researchers and managers (Gurevitch et al., 2016; Jackson & Fahrig, 2012). Therefore, we present an approach to define biologically informed, multi‐scaled, and hierarchically nested population units that could improve evaluation of population changes and thus increase effectiveness of wildlife management.

Modularity is one approach to defining biologically relevant subpopulations, conservation units, or biodiversity management units that partition the landscape into modules or subpopulation units (Peterman et al., 2016). Modularity relies on graph theory, a mathematical construct of edges connecting nodes (van Steen, 2010), to capture similar functional (species' responses to the environment) aggregations of patches (nodes representing populations or important habitat areas) as modules where interactions within groups are more similar than among groups. However, modularity approaches have led to a single scaled unit while analyzing a multitude of data, such as genetic networks (Peterman et al., 2016), mark‐recapture data (Reichert et al., 2016), and habitat and dispersal data (Gao et al., 2013). Because landscape configuration within habitats can affect individual movements (Ritchie, 2010), most species are spatially structured (i.e., population structure) at multiple scales due to differences in dispersal abilities (Ryberg et al., 2013). Therefore, these characteristics can build on the concepts of modularity to define multi‐scaled and biologically relevant management boundaries.

Population structure can describe differences in allele frequencies (Cardon & Palmer, 2003) and capture information about the landscape affecting the configuration of populations (Brown et al., 2013), habitat use, and dispersal patterns that characterize population connectivity (O'Donnell et al., 2022a). Graph theory, via the use of least‐cost paths (edges) and habitat patches (nodes), can capture the spatial structure of populations while accounting for multiple scales of dispersal movements informed from structural (e.g., configuration of habitat patches), functional (e.g., species' responses to environmental conditions), and potential (physical attributes of the landscape affecting dispersal ability) connectivity (Calabrese & Fagan, 2004; O'Donnell et al., 2022a; Tischendorf & Fahrig, 2000). Therefore, population structures constructed from graph theory may help inform the development of multi‐scaled modules (i.e., population units).

Researchers investigating wildlife population dynamics for conservation often assume population closure (i.e., there is no change due to births, deaths, immigration, and emigration) during a survey period to minimize bias of detectability when estimating abundance (Gardner et al., 2018; Iijima, 2020). The assumption of demographic closure (Stanley & Burnham, 1999) is often met by surveying species' populations during shorter periods and life stages that reduce occurrences of births and deaths. Geographic closure assumes there is no immigration and emigration during a survey period, which researchers have accomplished by using physical features (e.g., nets surrounding survey area where electrofishing is used for sampling) to prevent movements during surveys (Wathen et al., 2017), but is only tenuously met for studies of population dynamics (McDevitt et al., 2021). Using ecologically relevant boundaries that encompass frequent and shorter‐distance movements between survey sites can function as a “virtual net” and therefore reduce effects of immigration and emigration during a survey period to meet the assumption of geographic closure.

Ignoring or assuming a single scale that best characterizes a system can lead to ineffective management decisions (Cash et al., 2006; Fattorini et al., 2020). Assessing wildlife populations with multiple scales and across scales (hereafter, cross‐scale linkages) is important because processes affecting change occur at different institutional, spatial, and temporal scales (Cash et al., 2006). Co‐management by institutions occurs at multiple scales (e.g., local, regional, and national) that will not necessarily align with hierarchical scales of ecological processes (Keeley et al., 2022). The spatiotemporal scale at which researchers choose to investigate population dynamics of a particular species is critical because different patterns can emerge at different scales (Bissonette, 2017; Fuhlendorf et al., 2002; McGarigal et al., 2016). For example, population data collected at fine scales are commonly aggregated to determine trends at larger scales; however, extrapolation errors of population demographic data can arise without appropriate consideration of the scales in which biological processes occur (Bissonette, 2017). One solution is to estimate, for example, population rates of change across multiple hierarchical scales, allowing estimates to reflect ecological phenomena occurring at different scales (Coates et al., 2021; Edmunds et al., 2018). Another benefit of cross‐scale assessments is the ability to investigate changes of population demographics within and among scales, which could, for example, improve understanding of meta‐population dynamics (source‐sink dynamics) because changes to populations and functional habitat use are driven by ecological processes occurring at different spatial scales. The cross‐scale assessments can also increase support to managers when identifying local population changes relative to regionally connected populations that may or may not be changing.

Multi‐scaled approaches have additional benefits for researchers and managers. For example, they can increase support of adaptive management practices, where knowledge of a system is incomplete or even incorrect, yet new management prescriptions can be based on learned knowledge through previously failed attempts (Allen et al., 2011; Williams, 2011; Williams et al., 2009). Assessments of cross‐scale linkages may increase the inclusion of political and cultural differences affecting natural resource management practices which can improve adaptive management (Wilson et al., 2006). With multi‐scaled population management units and their capacity to assess cross‐scale population linkages, we can investigate, compare, and learn how populations vary spatially and temporally, thereby potentially improving management. Examples of adjusting mismatches of management units and scale that encapsulate movements have been demonstrated for marine fish (Kerr et al., 2017), turtles (Wallace et al., 2010), and ungulates (Meisingset et al., 2018), allowing for improved management actions. Hierarchically nested population levels may also support investigations of temporal issues surrounding population demographics, which could not be addressed with fixed‐scale population units. For example, managers may encounter challenges in deciding upon the need and timing of appropriate management actions if populations cycle (Jones, 2017; Myers, 2018)—especially when variation of periodicity (i.e., wavelength) and amplitude reflect stochasticity. The utility of cross‐scaled linkages may aid researchers and managers when working with species that have cycling populations (e.g., Coates et al., 2021).

We aimed to develop population management units that could address the following needs to aid wildlife management: (1) define biologically meaningful boundaries, (2) produce nested levels of population units that could describe local and regional processes affecting populations (i.e., scale‐dependent factors), (3) encapsulate wildlife movements approximating geographic closure during a survey period when demographic data are collected, (4) account for wildlife with cycling population trends that may vary spatially and temporally, and (5) provide a systematic method of defining population units that scientists and practitioners can use to manage populations. With hierarchically nested and biologically relevant population units, we can later assess population demographic data across scales to examine biologically relevant population trends and mechanistic studies of wildlife responses to landscape changes and processes occurring at multiple temporal and spatial scales. Importantly, our framework to define hierarchically nested population units provides the flexibility of including species‐relevant information, allowing methods to be applied to other species that have site fidelity during a life stage and when general dispersal and habitat selection information are known. As a case study, we applied our framework to greater sage‐grouse (*Centrocercus urophasianus*; hereafter, sage‐grouse) across their range within the western United States to assist managers with making informed, targeted, and cost‐effective decisions within an adaptive management framework.

## 2 MATERIALS AND METHODS

### 2.1 Modeling overview

**FIGURE 1:**
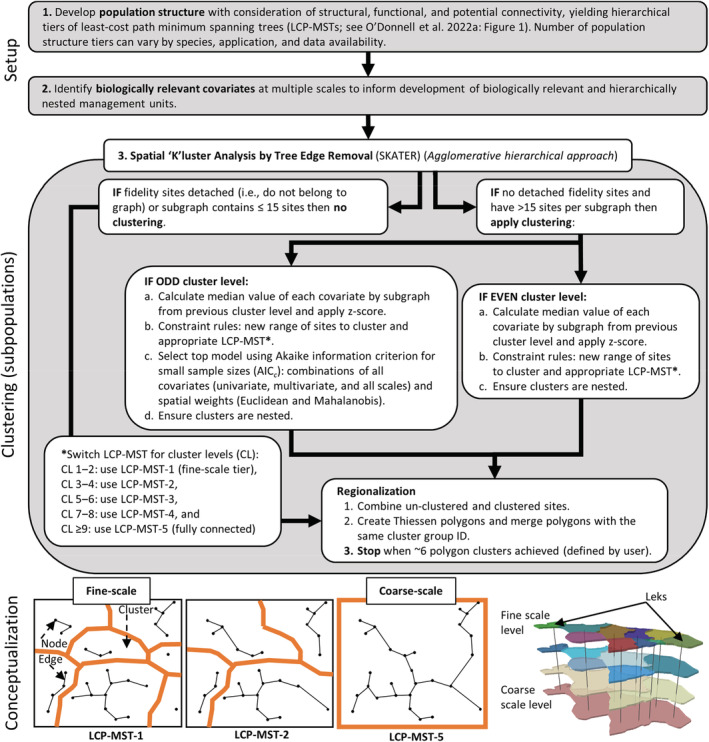
Scientific workflow for developing biologically relevant units of hierarchically nested populations for species with high site fidelity. Our case study used greater sage‐grouse (*Centrocercus urophasianus*) lek sites (high‐fidelity breeding sites) within the western United States. Step one (top) refers to the development of population structures based on a least‐cost path minimum spanning tree (LCP‐MST) and decomposition of the fully connected graph into population structure tiers described in O'Donnell et al. (2022a). The focus of this study is the clustering of sites of fidelity and delineation of subpopulation units to help inform adaptive management.

We developed an approach that defines biologically relevant population units (hereafter, population units) characterized by hierarchically nested population levels (akin to cluster levels, each representing a collection of subpopulations of aggregated sties of fidelity) (Figure 1). The general approach relied on known locations of site fidelity (e.g., breeding sites) and habitat data (e.g., terrain indices, vegetation cover, and climate). First, we developed population structure (i.e., spatial connectivity among populations) graphs (O'Donnell et al., 2022a) as a least‐cost path minimum spanning tree (LCP‐MST), which we then decomposed into hierarchical population structures based on dispersal distances (potential connectivity) and functional connectivity (e.g., factors attenuating movements). The least‐cost path (LCP) captures the shortest cumulative cost‐weighted distance based on the distance and cost to travel between two points (Etherington, 2016). The fully connected graph represented a minimum spanning tree (MST), denoting the smallest total cost of all connections among nodes (e.g., leks; Cormen et al., 2009). These population structure tiers reflect hierarchical levels (as subgraphs), describing dispersal frequency and factors affecting avoidance to identify modules or groupings of structure (O'Donnell et al., 2022a; Ryberg et al., 2013). Second, we acquired and developed relevant habitat covariates to inform the clustering of high‐fidelity sites. Third, we used the hierarchical population tiers and habitat covariates summarized at multiple spatial scales (e.g., landscape surrounding breeding sites averaged at multiple radii) encompassing each high‐fidelity site as data inputs to the clustering algorithm, Spatial “K”luster Analysis by Tree Edge Removal (SKATER; AssunÇão et al., 2006; AssunÇão et al., 2012).

We uniquely developed a framework of steps, allowing us to define a hierarchical schema using SKATER, where clustering was agglomerative (bottom‐to‐top). These steps broadly included starting with the least connected population structure tier (i.e., LCP‐MST with the greatest number of subgraphs), testing all combinations of covariates, and selecting the best model. For each cluster level, we defined a different range of sites with fidelity to cluster (i.e., constraint‐based rule). We swapped out a different population structure for each odd cluster level (e.g., 1, 3, and 5). With even cluster levels (e.g., 2, 4, and 6), we used the top model selected from the previous odd cluster level for a more parsimonious approach. In other words, the approach of not selecting a different population structure and combination of covariates allowed for a progression of the clustering. For example, level 2 (an even level) used the same information as level 1 (an odd level) to slowly group the leks before considering new covariates and larger landscapes for level 3 (an odd level). After completing each cluster level, we defined Thiessen polygons (Thiessen, 1911) around the sites of fidelity assigned to clusters, thereby partitioning the landscape into contiguous population units. Thiessen polygons define the equal distance between non‐similar classified points where any location inside the polygon is closer to the point(s) used to define the polygon.

### 2.2 Case study background

**FIGURE 2:**
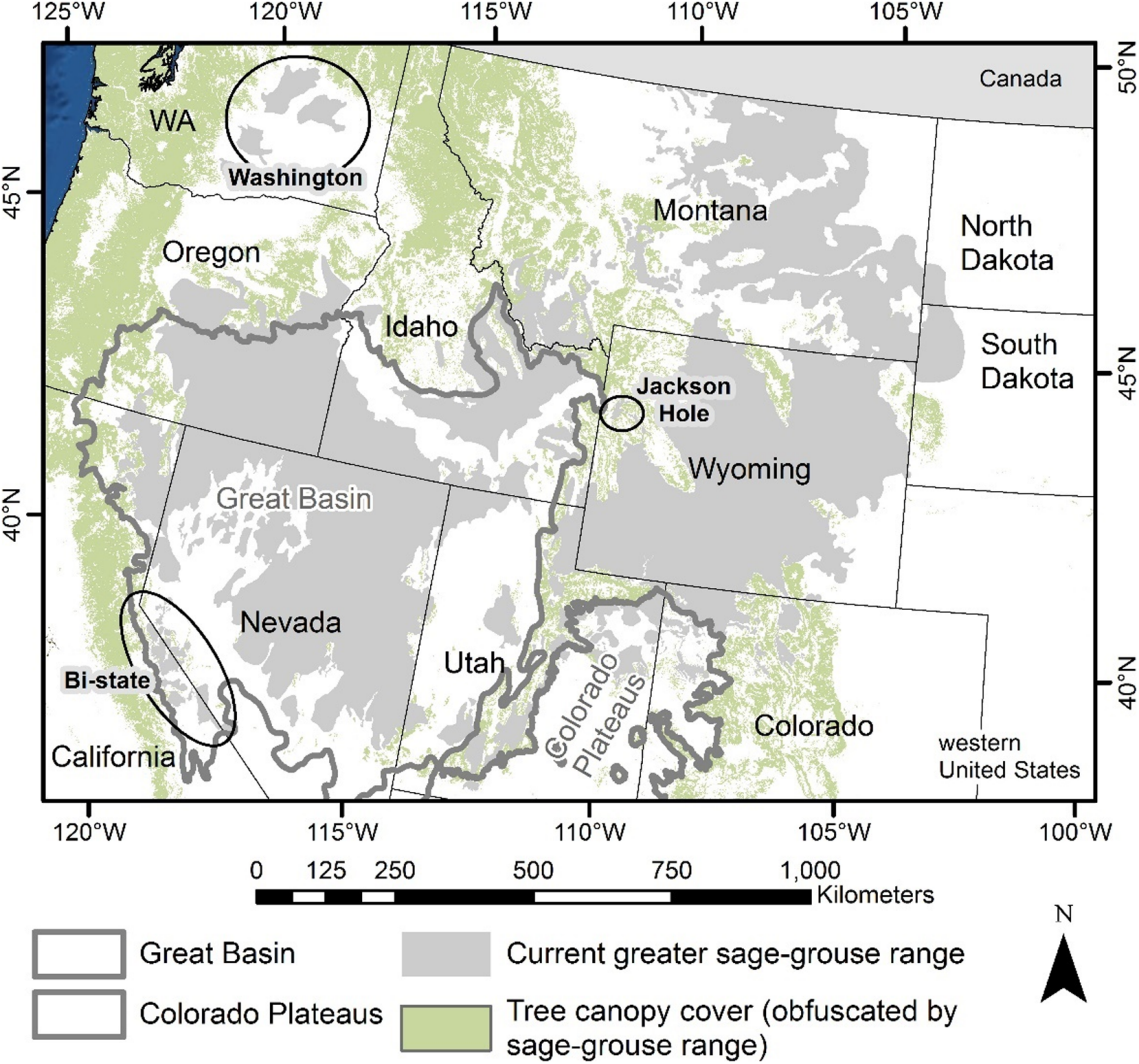
Current greater sage‐grouse (*Centrocercus urophasianus*) range in the western United States. Washington (WA), Bi‐state (Nevada‐California), and Jackson Hole (Wyoming) represent three genetically isolated sage‐grouse subpopulations.

Sage‐grouse are a sagebrush obligate species (Hanser & Knick, 2011; Rowland et al., 2006) located within the sagebrush biome of the western United States (Figure 2). The sagebrush biome includes many mountain ranges with salt deserts in the Great Basin and Colorado Plateau. It is characterized by shrubs interspersed with grasses and forbs at lower elevations and forests at higher elevations (Pyke et al., 2015). The ecosystem predominately includes sagebrush (*Artemisia* spp.) with less dominant non‐sagebrush species of rabbitbrush (*Chrysothamnus/Ericameria* spp.) and bitterbrush (*Purshia* spp.; Remington et al., 2021, chapter A). During recent decades, fragmentation, habitat loss, and degradation have increased due to multiple factors including energy development, agricultural conversion, urbanization, conifer expansion, and wildfire and annual grass invasion (Knick & Connelly, 2011; Remington et al., 2021, chapters J‐P). The current sage‐grouse range (United States and Canada) is ~56% of its pre‐European settlement extent (Schroeder et al., 2004), with 0.83% to ~3% annual population declines occurring since 1965 (Coates et al., 2021; Connelly et al., 2004; Garton et al., 2015; Western Association of Fish and Wildlife Agencies, 2015). Sage‐grouse population sizes oscillate with varying degrees across the species range (Coates et al., 2021; Edmunds et al., 2018; Fedy & Aldridge, 2011; Garton et al., 2011), and these degrees of cyclicity might be related to climate at broad scales (e.g., Coates et al., 2018) or fragmentation of the landscape at finer scales, where the latter has been shown to reduce the cycles' amplitude and period for some species (Myers, 2018).

Sage‐grouse are an advantageous case study because they have been monitored by state natural resource agencies since the 1950s and are of conservation concern due to significant range contraction and population declines. Cross‐scale linkages for co‐management of sage‐grouse have become increasingly important to avoid the U.S. Fish and Wildlife Service (USFWS) listing the species under the Endangered Species Act (ESA). The USFWS declared in 2015 that listing of sage‐grouse under the ESA was not warranted (U.S. Fish and Wildlife Service, 2015), but called for greater integration of adaptive management into land‐use planning. Our objective was to hierarchically group sage‐grouse high‐fidelity breeding sites (leks) based on biological inferences that captured approximately closed population units (i.e., geographic closure). This approach was similar to O'Donnell et al. (2019a), where these methods were piloted for sage‐grouse in Nevada and Wyoming. Here, we employed important and extensive enhancements (supplemental S1 in Appendix S1) to the data and workflow for a range‐wide application across 11 western states (United States). Broadly, these included a newly standardized lek database (O'Donnell et al., 2021), methods to define population structure graphs (hierarchical population tiers; O'Donnell et al., 2022a), additional candidate habitat covariates, modifications to clustering workflows, and a significant increase in marked birds for evaluation. Because we transitioned from two disparate and non‐contiguous states to the complete sage‐grouse range, we eliminated problems with confining population units to jurisdictional state boundaries (O'Donnell et al., 2019a). We worked collaboratively with lead sage‐grouse biologists from 11 western states, who formed the Sage and Columbian Sharp‐tailed Grouse Technical Team (hereafter, Technical Team; see Acknowledgements), to evaluate methods and results in a co‐production environment. We developed Python™ (Rossum & Drake, 2009) and program R (R Core Team, 2020) open‐source software (*popcluster* v.2.0) for all analyses, which we had revised from the pilot (*popcluster* v.1.0; O'Donnell et al., 2019b).

### 2.3 Population structure overview

Our approach for defining sage‐grouse population structure relied on active sage‐grouse lek locations and a resistance surface. We used a range‐wide, standardized greater sage‐grouse lek (breeding display ground) database (O'Donnell et al., 2021) to define population structures developed by O'Donnell et al. (2022a). The lek database included multiple types of information, such as lek locations (high site fidelity to breeding grounds) and conservation status. We standardized state lek count databases by fixing typos, assigning standard columns names, standardizing how leks are classified, and made significant data improvements allowing for a range‐wide database. Leks used in the development of population structure and population units included “Active” and “Pending New” classifications of conservation status (*n* = 5832 leks), which best reflect current conditions and habitat use across the species' range. Active leks represented leks with ≥2 males per 2 observations occurring on different years recorded in the previous 10 years. Pending New leks required one observation of ≥2 males in the previous 10 years and at least one observation of ≥2 males longer than 10 years ago. With the exception of using counts of individuals to define active leks, we did not use demographic data for defining population structure or population units. Sage‐grouse return to lek locations each year and across generations (Connelly et al., 2003) and therefore are used for monitoring populations by state wildlife agencies. Sage‐grouse disperse ~3–7 km from leks to nesting habitat (Dahlgren et al., 2016; Holloran et al., 2005; Wakkinen et al., 1992) and typically disperse farther distances for brood‐rearing, summer, and winter habitats (O'Donnell et al., 2019a: see supplemental S6 in Appendix S1).

**FIGURE 3:**
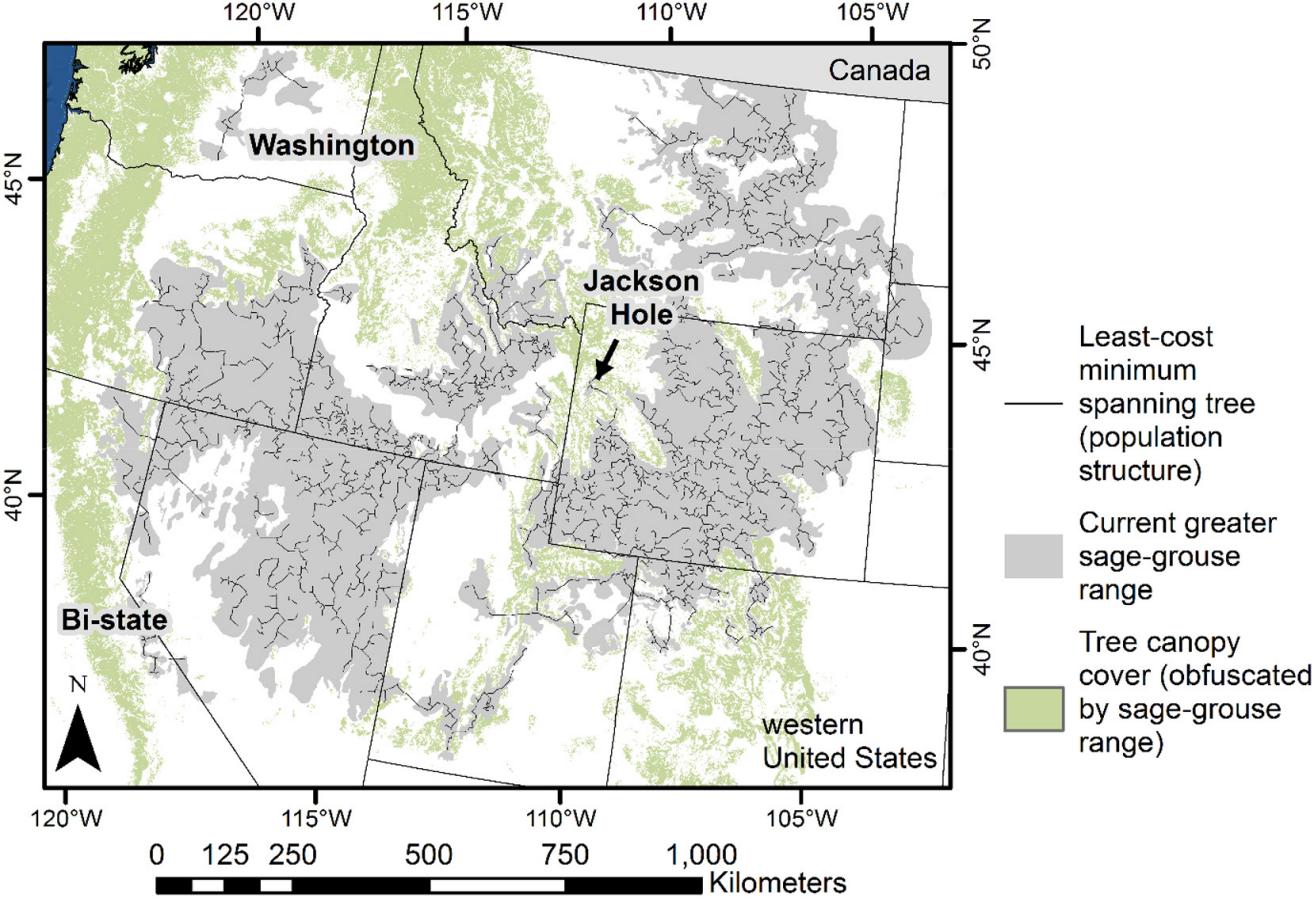
Population structure of greater sage‐grouse (*Centrocercus urophasianus*) in the western United States. We used active sage‐grouse leks to define the current population structure (breeding display grounds) and biological inferences. These inferences encompassed dispersal capabilities, genetic flow, seasonal habitat conditions, and functional processes (e.g., selection of habitat at multiple scales) affecting movements (O'Donnell et al., 2022a).

The resistance surface reflected costs of movements between neighboring leks based on habitat conditions preferred by sage‐grouse. For example, the resistance surface captured how sage‐grouse generally avoid traversing large mountain ranges and rugged terrain, dense forests, large water bodies, and salt flats but select for greater sagebrush cover (O'Donnell et al., 2022a: see Figure S1). Using the resistance surface and sage‐grouse lek locations, we generated least‐cost paths (edges) between neighboring leks (nodes) to define a fully connected graph (population structure; Figure 3) with a distance‐weighted cost value assigned to each edge (O'Donnell et al., 2022a).

We then decomposed the fully connected population structure into hierarchical tiers of subpopulation structures (i.e., subgraphs) that can provide additional rules of clustering leks. The decomposition was accomplished by removing edges between nodes to reflect patterns of dispersal capabilities (e.g., 15 km inter‐lek movement distance [95th percentile]; O'Donnell et al., 2019a), genetic flow, genetic isolation, and landscape features attenuating movements (e.g., roads with high traffic volumes) that describe functional and potential connectivity (O'Donnell et al., 2022a: see Table 1). Any sites of fidelity not connected to a graph (i.e., detached lek) were a result of removing an edge and isolating the node/lek from neighboring sites. These hierarchical tiers of population structure reflect a range of short and more frequent dispersals (smaller subpopulation units) to far and less frequent dispersals (larger subpopulation units). For example, tier one (e.g., least‐cost path minimum spanning tree, tier 1 [LCP‐MST‐1]) has the greatest number of subgraphs (reflecting shorter dispersals and less connected high‐fidelity sites), tier four (e.g., LCP‐MST‐4) has the fewest number of subgraphs, and tier five (e.g., LCP‐MST‐5) is fully connected except for three genetically isolated populations (Washington, Nevada, and California Bi‐state region, and Jackson Hole, Wyoming; O'Donnell et al., 2022a). Here, we used the population structure tiers and associated edge weights as connectivity rules (i.e., constraint‐based rules) to help inform the clustering of leks into nested population levels of subpopulation units. For example, the population structure tiers described gradients of connectivity among populations and represented rules on when connectivity increased as populations were aggregated at coarser scales.

**TABLE 1:** Examples of species that one could potentially develop biologically relevant and hierarchically nested population units for wildlife management

Species (conservation importance)	Geographic location	Years monitored (examples)	Site fidelity type and known locations	Dispersal and subpopulation information (References)	References (examples)
Lesser prairie chicken (*Tympanuchus pallidicinctus*)^a^	NM‐TX: New Mexico (NM) and Texas (TX) CO‐KS‐OK: Colorado (CO), Kansas (KS), and Oklahoma (OK)	NM‐TX: 1971–2018 Species' range: 2002–2012	Lekking (breeding): open sites of shortgrass prairies NM: *n* = 1023 Species' range: *n* = 1257	Dispersal: mean of 8.8–16.2 km Subpopulations: NM‐TX versus CO‐KS‐OK	Schilder et al. (2022) Jarnevich et al. (2016) Jarnevich et al. (2021) Earl et al. (2016)
Booted eagle (*Hieraaetus pennatus*)^b^	Europe, North Africa, Asia, western South Africa, and Namibia (European populations generally migrate to Africa during winter)	Spain: 1998–2015 Armenia: 2003–2019	Territorial nesting; some populations migrate Spain: *n* = 163 Armenia: *n* = 21–29	Dispersal: 25 km from nest locations Subpopulations: territories can include one (typical) to several nests	Martínez, Pagán, and Calvo (2006) Martínez, Pagán, Palazón, et al. (2006) Jiménez‐Franco et al. (2020) Suârez et al. (2000) Aghababyan and Stepanyan (2020)
Polar bears (*Ursus maritimus*)^c^	Alaska (AK), U.S. Canada (CAN) Circumpolar	AK: 2001–2016 (count data) CAN: 2011–2016 Circumpolar: 1967–2018; 1910–2018; 1970–2018 (harvests by subpopulation)	Denning (sites/areas): primarily along shorelines but also sea ice AK: *n* = 530 CAN: *n* = 1593 Circumpolar: *n* = Unknown	Dispersal: >1000 km annually, movement distances becoming shorter for some populations due to food shortages and lack of ice needed hunting; home ranges as large as 133 km^b^ Subpopulations: 19–20	Florko et al. (2020) Durner et al. (2020) Vongraven et al. (2012) Vongraven et al. (2022) Atwood et al. (2020) Durner et al. (2018) Brun et al. (2021) Parks et al. (2006) Amstrup et al. (2005) Koehler et al. (2019) Laidre et al. (2022) Obbard et al. (2018)

*Note*: Monitoring data are not required to define population units using methods we have outlined (i.e., defining spatial population structure and clustering sites of high‐fidelity using habitat covariates), but they are necessary to assess demographic data using the hierarchical population units. We provide a sample of information about each species to demonstrate species with high site fidelity and where studies and monitoring have occurred; however, we expect our methods would be useful for many other species.

^a^ Northern distinct population segments (DPS) are proposed as threatened, while the southern DPS is proposed as endangered (https://www.fws.gov/lpc, accessed 6 Oct 2022).

^b^ Listed as least concern under the International Union for Conservation of Nature (IUCN) Red List (https://www.iucnredlist.org/species/22696092/206456835, accessed 6 Oct 2022).

^c^ The International Union for the Conservation of Nature (IUCN) lists polar bears as “vulnerable to extinction” while the U.S. Fish and wildlife Service under the Endangered Species Act listed them as threatened with extinction (U.S. Fish and Wildlife Service, 2021).

### 2.4 Habitat covariates

We considered important candidate habitat covariates and scales identified in resource selection functions defining habitat suitability for clustering (Coates et al., 2019; Fedy et al., 2014; Rice et al., 2016; Saher et al., 2022). The continuous habitat covariate data included terrain indices (5 datasets), percent cover of shrubland vegetation communities (10 datasets; Rigge et al., 2021), and bioclimatic variables (5 datasets; O'Donnell & Ignizio, 2012). For each covariate, we used zonal statistics to summarize the spatial data surrounding a lek within seven radii (an additional three radii were used for terrain indices) occurring between 30 m and 6400 m (7–10 datasets). Although 30‐m pixel sizes are small, this scale is commonly considered in sage‐grouse species distribution models (Coates et al., 2019; Fedy et al., 2014; Rice et al., 2016; Saher et al., 2022). The pixels associated with each lek are also summarized across all leks in a group (a minimum of 15 leks), so the combined area is larger than 30 m. We calculated arithmetic mean and coefficient of variation (standard deviation / mean) for the zonal statistics (see supplemental S2 and Tables S1 and S2 for definitions of indices, data sources, scales, and statistics).

We used environmental variables describing habitat conditions and lekking site fidelity as a method to group leks with similar abiotic and biotic conditions at multiple spatial scales. Habitat conditions are dynamic and functional habitat loss can lead to new movements and shifts in population distributions. The habitat conditions alone do not necessarily describe fitness consequences (e.g., source‐sink dynamics), which has been shown for sage‐grouse (Aldridge & Boyce, 2007; Brussee et al., 2022; Kirol et al., 2015; O'Neil et al., 2020) and other species (Williams et al., 2021).

### 2.5 Developing population units

Clustering analyses are used to group objects that maximize similarities within clusters and dissimilarities among clusters. Many clustering algorithms exist (Wegmann et al., 2021; Xu & Tian, 2015) to handle different data types, such as spatial (Varghese et al., 2013; Wang & Wang, 2010) and non‐spatial data (Carey et al., 2016), and objectives (Estivill‐Castro, 2002; Jain, 2010; Kleinberg, 2002). Clustering is frequently used as a data mining exercise and although we have identified relevant habitat covariates, we have considered many candidate covariates, scales per candidate, and combinations of covariates. Our objective for clustering sage‐grouse leks was to group breeding sites located within similar habitats using an algorithm that supports regionalization (partitioning) of adjacent leks into contiguous geographic space (AssunÇão et al., 2006; van Steen, 2010). We chose the SKATER algorithm (AssunÇão et al., 2006, 2012) because it supports regionalization through graph theory, the inclusion of one or more covariates, adjacency of connected nodes in the graphs, and the ability to use different pairwise distance measures between multivariate attributes (e.g., cartesian aspatial weights of Euclidean and Mahalanobis to assess covariate similarities). The SKATER algorithm uses a fully connected graph with nodes and edges. For the assignment of nodes (e.g., leks) to groups (i.e., subpopulations), the graph is iteratively pruned (i.e., decomposed) based on two objective functions that result in the largest sum of squared deviations and approximately equal‐sized subgraphs (AssunÇão et al., 2006, 2012).

Currently available clustering algorithms that partition landscapes do not create hierarchically nested spatial clusters. Therefore, we applied an agglomerative approach using a workflow (Figure 1) that achieves a spatially hierarchical clustering algorithm yielding nested clusters. Some clustering approaches support constraint‐based rules, providing domain knowledge and semi‐supervised clustering (Bair, 2013). Such approaches are typically accomplished using tree‐based algorithms (e.g., decision trees and random forest) that include rules about barriers (lack of connectivity), number of features to cluster, flow rates, and distance thresholds (Bair, 2013; Basu et al., 2008; Rooijen et al., 2021). However, SKATER—like many algorithms—has limited support for constraint‐based rules. We used the hierarchical tiers of population structure, denoting degrees of connectivity, as constraint‐based rules during the clustering. SKATER natively uses a graph of Euclidean distances (least‐cost minimum spanning tree; LC‐MST) that may lack representation of habitat connectivity (O'Donnell et al., 2022a). Therefore, we substituted the LC‐MST used by SKATER with our LCP‐MSTs, representing the hierarchical tiers of sage‐grouse population structure.

The clustering of sage‐grouse leks using SKATER incorporated hierarchical sage‐grouse population structures, candidate habitat covariates summarized at multiple spatial scales of each lek, and a distance measure of attribute space (i.e., aspatial weight of Euclidean and Mahalanobis). We implemented the SKATER model via *popcluster* using the function *skater* (spdep library; Bivand & Piras, 2015), which requires a graph with nodes (e.g., leks with assigned covariate values) and edges (e.g., population structure tiers with attribution of the cost to move between nodes) for clustering. Before clustering occurred at each level, we calculated the median value of covariates assigned at leks within a subgraph per cluster level and applied a z‐score transformation to standardize the covariate units (O'Donnell et al., 2019a). The SKATER clustering algorithm was applied using an agglomerative approach (i.e., fine‐scale clusters to coarse‐scale clusters). We swapped out population structure tiers at every other cluster level (e.g., tier‐one informed cluster levels one and two and tier five informed cluster levels nine and greater), where connectivity increased with subsequent aggregation of leks. We also specified a different range of leks for grouping at each cluster level (constraint‐based rule; supplemental, Table S3), avoiding the need to select the number of clusters to generate and to increase support of a spatially balanced distribution of population units. Because lek groupings must remain nested across cluster levels, which is not a criterion of the SKATER algorithm, the software (*popcluster*) adjusts groupings to enforce nesting. Each population structure tier included multiple subgraphs where SKATER clustered leks located on each subgraph independently. Therefore, selection of the best combination of habitat covariates, scale, summary statistics, and aspatial weights were determined for each subgraph (i.e., denotes a single model) of the population structure tier.

We selected the best combination of data for each subgraph within each odd cluster level using Akaike information criterion corrected for small sample sizes (AIC_*c*_) calculated from the sum of squared errors (Ashour et al., 2018; Banks & Joyner, 2017; Burnham et al., 2011). For each subgraph, we report the top three models. The clustering was iteratively applied until reaching ≤6 populations of clustered leks (number defined by user depending on application need). We identified six for our study because we had three genetically isolated populations and three additional subpopulations seemed reasonable for the species (species' range usually split east and west). After generating all clusters, we modified lek assignment and population units if grouped leks were not adjacent and then partitioned the landscape using Thiessen polygons of leks clustered into groups. Each subgraph defined by the five LCP‐MSTs reflected a different model, and we summarized the relative importance of selected covariates, scale, and aspatial weights by calculating the number of leks associated with top model covariate divided by total number of leks (O'Donnell et al., 2019a).

### 2.6 Evaluating population units

The purpose of evaluating population units was to determine how well polygons (clusters, representing population units) within each cluster level captured individual home ranges of birds and to identify the smallest cluster level that could support population models (i.e., cluster level that most closely approximated geographic closure). We worked with the Technical Team to evaluate cluster assignments based on expert knowledge of sage‐grouse movements and habitat use. For the evaluation, we discussed with individual states and the full Technical Team the clustering approaches and results, allowing us to incorporate local expert knowledge and perspectives. State lead biologists from the Technical Team reviewed each cluster level, respective to their state, and provided feedback on any warranted changes. Also, in several group settings, all states and authors discussed the methods and resulting population units to facilitate discussions and product improvements. We encountered occurrences where *skater* did not consistently capture nesting of lek assignments to clusters or did not consistently enforce adjacency of nodes in the graph. Therefore, we evaluated and reported on these occurrences. Additionally, we evaluated the clusters using empirical data distributed across the sage‐grouse range, including location data collected from very high frequency (VHF) and global positioning system (GPS) marked sage‐grouse from multiple field studies.

Researchers studying sage‐grouse used our *popcluster* software to analyze their independent data collected from respective studies (see Acknowledgements). With *popcluster*, we assessed all use locations based on a biological year (i.e., movements during all life stages within a year, beginning during the breeding season [March 1]). The GPS and VHF location data were collected from both males and females of three age classes (juvenile, yearling, and adult). The temporal frequency for GPS locations varied among and within studies and ranged between one and three hours when provided. For evaluating clusters with VHF and GPS data, we defined a home cluster for each bird by identifying the polygon (cluster) that contained the maximum number of use locations (VHF) or amount of time (GPS) each bird occurred within a biological year. We calculated the proportion of each bird's locations falling outside its home cluster across all biological years to evaluate VHF data. For evaluating clusters with GPS data, we used the *brownian.bridge.dyn* function, a dynamic Brownian bridge movement model (dBBMM; Kranstauber et al., 2012) available in program R (library move; Kranstauber et al., 2020), and calculated the proportion of time birds spent outside their designated home cluster. The dBBMM defined the home range (utilization distribution) by accounting for the temporal information of the GPS data and allowing the bridge to expand and contract based on the length of time between successive locations and the Brownian motion variance. We then calculated the mean and standard error (SE) for the amount of time and proportion of use locations individual birds spent outside their home cluster (polygon) for each cluster level. We expected individuals to spend most of their time within a single population unit, approximating a geographic closure; however, we did not know which of the smaller cluster levels would yield the best results.

We also evaluated the effect habitat covariates had on the delineation of population units by excluding these from the analyses. For this analysis, we needed to include the population structure tiers in the clustering workflow, and the SKATER algorithm required a minimum of one covariate. Therefore, we calculated a random distribution of integers (0–1,000,000) and assigned the value to each lek. A random distribution of integers was used because if we had assigned a constant (e.g., 1) to every lek, the resulting model would yield the greatest similarity within clusters (within group sum of squares equals 0), resulting in calculating the log of zero and therefore no AIC_*c*_ value (O'Donnell et al., 2019a: see equation 1–6). This covariate was then used in *popcluster* to generate a new set of population levels using the identical workflow for when habitat covariates were considered.

## 3 RESULTS

We present the resulting population units and the most influential model components (i.e., covariates, scales, and spatial weights) selected from the top models. We also present the range‐wide results from evaluating population units with locations from VHF or GPS‐marked sage‐grouse and the effects habitat covariates played in defining population units. Details of all model selections and cluster evaluations are reported in supplemental S3 and S4, respectively.

### 3.1 Population units

**FIGURE 4:**
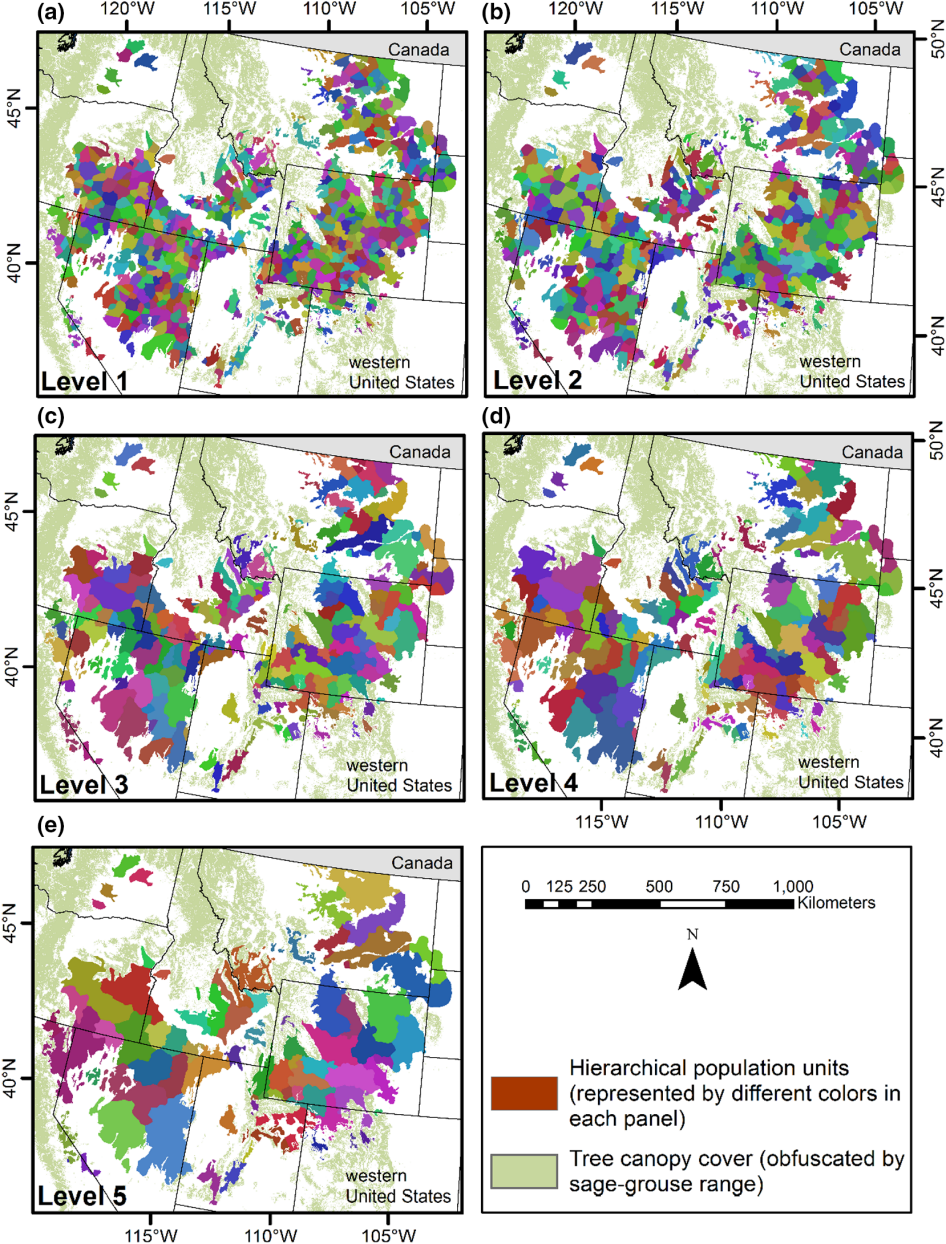
Hierarchical population levels comprised of subpopulation management units (cluster levels 1–5; respectively, Panels (a) – (e)) for greater sage‐grouse (*Centrocercus urophasianus*) in the western United States. We defined population units using sage‐grouse leks (breeding display grounds), hierarchical population structure, and habitat covariates at multiple scales with the Spatial “K”luster Analysis by Tree Edge Removal (SKATER) clustering algorithm. The polygons of subpopulation units reflect groupings of sage‐grouse lek sites, which were developed after leks were assigned/clustered.

**FIGURE 5:**
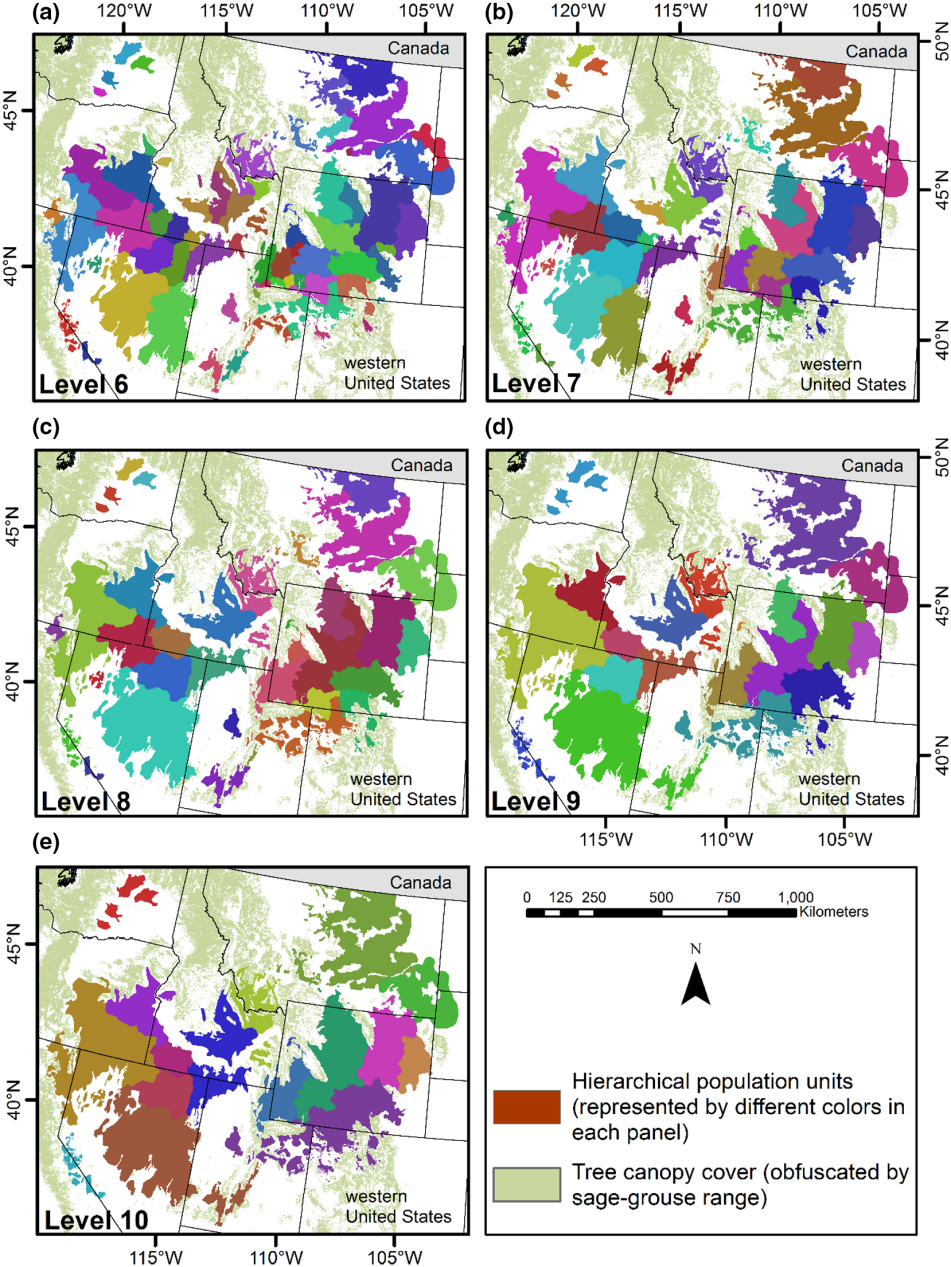
Hierarchical population levels comprised of subpopulation management units (cluster levels 6–10; respectively, Panels (a)–(e)) for greater sage‐grouse (*Centrocercus urophasianus*) in the western United States. Population units were defined using sage‐grouse leks (breeding display grounds), hierarchical population structure, and habitat covariates at multiple scales with the Spatial “K”luster Analysis by Tree Edge Removal (SKATER) clustering algorithm. The polygons of subpopulation units reflect groupings of sage‐grouse lek sites, which were developed after leks were assigned/clustered.

**FIGURE 6:**
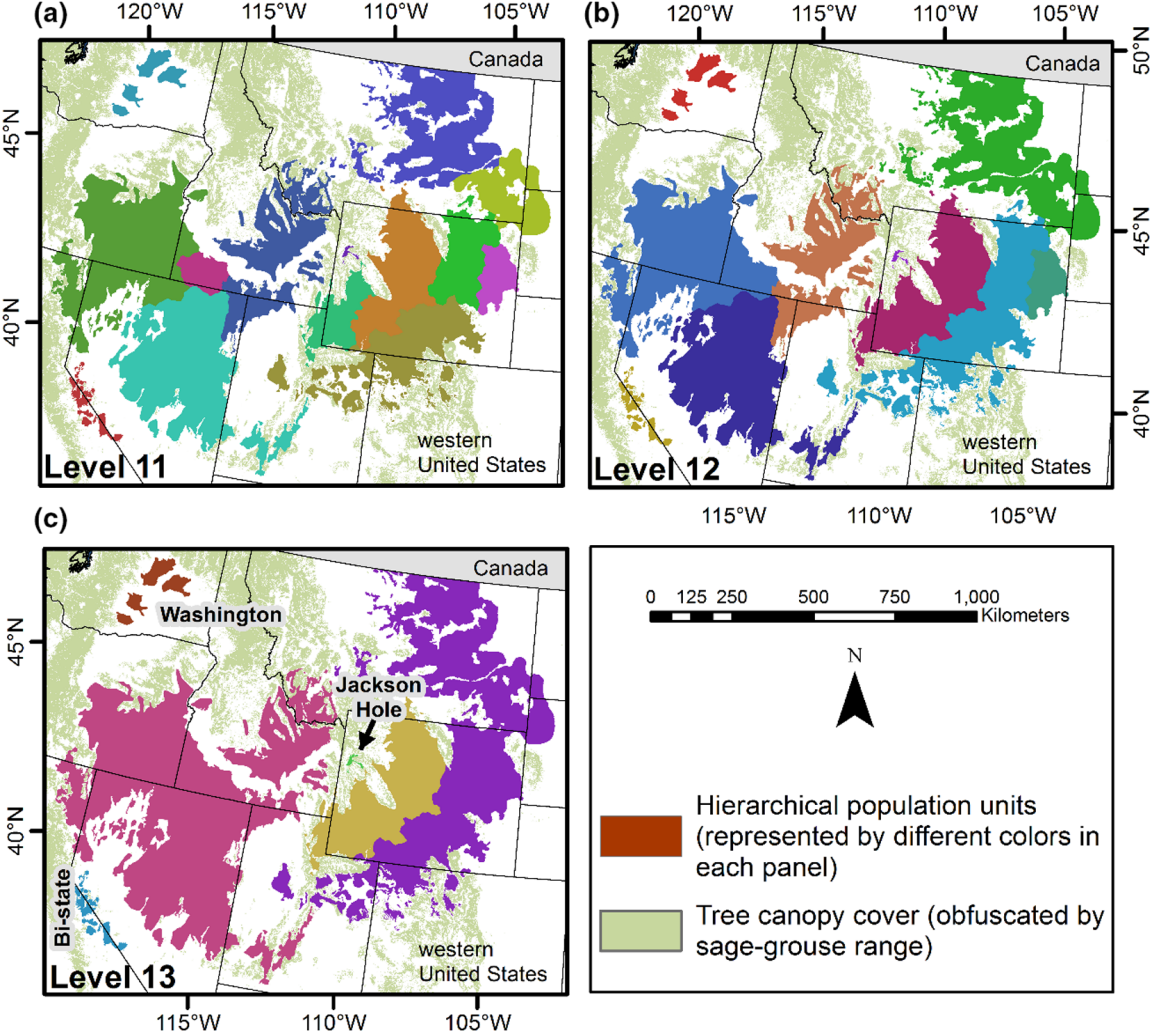
Hierarchical population levels comprised of subpopulation management units (cluster levels 11–13; respectively, Panels (a)–(c)) for greater sage‐grouse (*Centrocercus urophasianus*) in the western United States. Population units were defined using sage‐grouse leks (breeding display grounds), hierarchical population structure, and habitat covariates at multiple scales with the Spatial “K”luster Analysis by Tree Edge Removal (SKATER) clustering algorithm. The polygons of subpopulation units reflect groupings of sage‐grouse lek sites, which were developed after leks were assigned/clustered.

The clustering of sage‐grouse leks included 5832 active leks and resulted in 13 cluster levels, where cluster level 1 was the finest scale and level 13 was the coarsest scale (Figures 4, 5, 6). Cluster level 1 included 650 subpopulations, and level 13 delineated six subpopulations (supplemental, Table S4). Each cluster level contained a different number of subgraphs and detached leks (i.e., nodes disconnected from a graph; supplemental, Table S4), as defined by the population structure (Figure 3). Cluster levels 1–2 included the greatest number of detached leks (122) because they were farther than 15 km from the nearest neighbor, while cluster levels three and four had 12 each. These results occurred because of the influence the hierarchical population structures had on the clustering. They also indicate greater isolation of leks within Nevada, Oregon, eastern Wyoming, and northeastern Utah, while the most intact populations occurred in southern Idaho/northern Nevada and central and western Wyoming (supplemental Figures S2 and S3).

### 3.2 Influential model components

The relative importance of covariates selected in top models is explained here with details in supplemental (Figures S5–S9). At the finest scale population structure (LCP‐MST‐1), precipitation seasonality was most important (supplemental, Table S6 and Figure S4). Elevation and herbaceous cover were important for moderate scales (LCP‐MST‐2), while elevation, non‐big sagebrush percent cover, big sagebrush percent cover, and sagebrush height were important at coarse scales (LCP‐MST‐3 and 4; supplemental, Table S6 and Figure S6). The scales summarizing habitat with the greatest influence included 1500‐, 2200‐, 4700‐, and 6400‐m radii distances (supplemental, Figure S7). As clusters aggregated additional leks (e.g., LCP‐MST‐1 with cluster levels 1–2, LCP‐MST‐2 with cluster levels 3–4, and LCP‐MST‐3 with cluster levels 4–5), the landscape area (i.e., scale) selected in the top models generally decreased from 6400 to 1500 m (supplemental, Figure S7). The mean summary statistic (calculated from zonal statistics) for covariates used in models had the greatest influence, followed by the coefficient of variation and no summary statistic (no statistic used because not applicable to covariate; supplemental, Table S6 and Figure S8). In most cases, the Euclidean distance measure was the best cartesian distance weight to define population units (supplemental, Table S6 and Figure S9). Due to the large geographic extent of the species' range and the variability of habitat conditions, we expected variability in covariates and scales selected for the models (subgraphs of each population structure tier).

### 3.3 Evaluation

Suggested modifications from the Technical Team to the clusters required only changes to the hierarchical population structure tiers (O'Donnell et al., 2022a). The most important recommendation from states that affected our results was the exclusion of powerlines, which we initially included as a feature affecting connectivity of local populations. A few other recommendations included modifications to the connectivity of populations, which are fully described in O'Donnell et al. (2022a). With each cluster level, *popcluster* corrected non‐nested leks relative to the previous cluster level, resulting in 172 lek reassignments. The manual correction of adjacency (post‐cluster development) occurred more frequently (2007 lek occurrences), but these occurrences propagated across cluster levels because corrections were made after all results were generated (supplemental S4). Wyoming had the largest number of reassignments because a large cluster in northeast Wyoming and southeast Montana was incorrectly grouped (violation of adjacency rule; supplemental, Tables S7 and S8, Figure S10). Across all 13 cluster levels, there were 607 unique leks reassigned to different cluster groups to meet adjacency rules and hierarchical nesting (~10.4% of leks).

**FIGURE 7:**
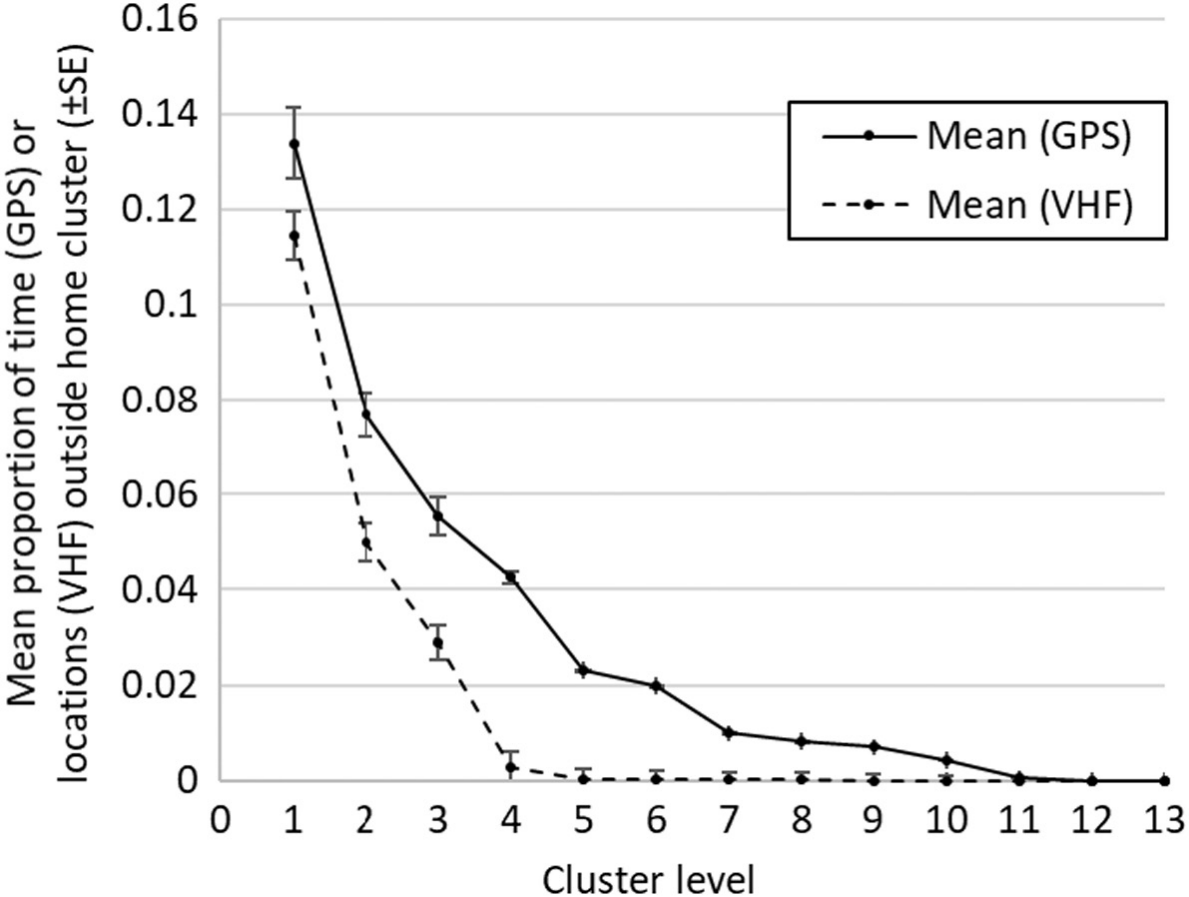
Very high frequency (VHF) and global positioning systems (GPS) location data used from multiple research studies across the greater sage‐grouse (*Centrocercus urophasianus*) range to evaluate hierarchical population units in the western United States. We used the VHF data to estimate the proportion of use locations observed outside the home range. The dynamic Brownian bridge movement model (Kranstauber et al., 2012) and GPS data were used to estimate the time spent outside the home range. We calculated the mean and standard error (SE; whiskers) of time and use birds spent outside their home cluster (polygon) for each population level (cluster level). Data include 1551 (GPS) and 1270 (VHF) uniquely marked birds, with collections spanning between 2006 and 2020 for a total of 1,685,443 (GPS) and 31,731 (VHF) observations (see supplemental, Tables S9–S15 and Figures S11–S13, for contributions and data details).

We used 1551 (GPS) and 1270 (VHF) uniquely marked birds, with studies spanning between 2006 and 2020 (median year = 2015 for GPS and median year = 2006 for VHF datasets) to analyze how well cluster polygons approximated geographic closure across all cluster levels. There were 1,685,443 GPS and 31,731 VHF locations (supplemental S4, Tables S9–S15, Figure S11). The results of the range‐wide assessment describing the proportion of time (GPS) and use (VHF) locations of individual birds that occurred inside their home cluster demonstrated cluster levels ≥2 captured >92% of time and > 95% of use (Figure 7). Each state captured >92% of time that birds remained within their home cluster when examining results at cluster level 2, except Idaho (~84.7%; supplemental, Figures S12 and S13). Cluster level 1 included greater movements of individuals into neighboring populations (13% [GPS] and 11% [VFH]), which we did not consider representative of approximating closed population units. Therefore, cluster level two reflected the smallest subpopulation units with an acceptable approximation of capturing geographic closure that also minimized the number of grouped leks, allowing for habitat management at local scales.

**FIGURE 8:**
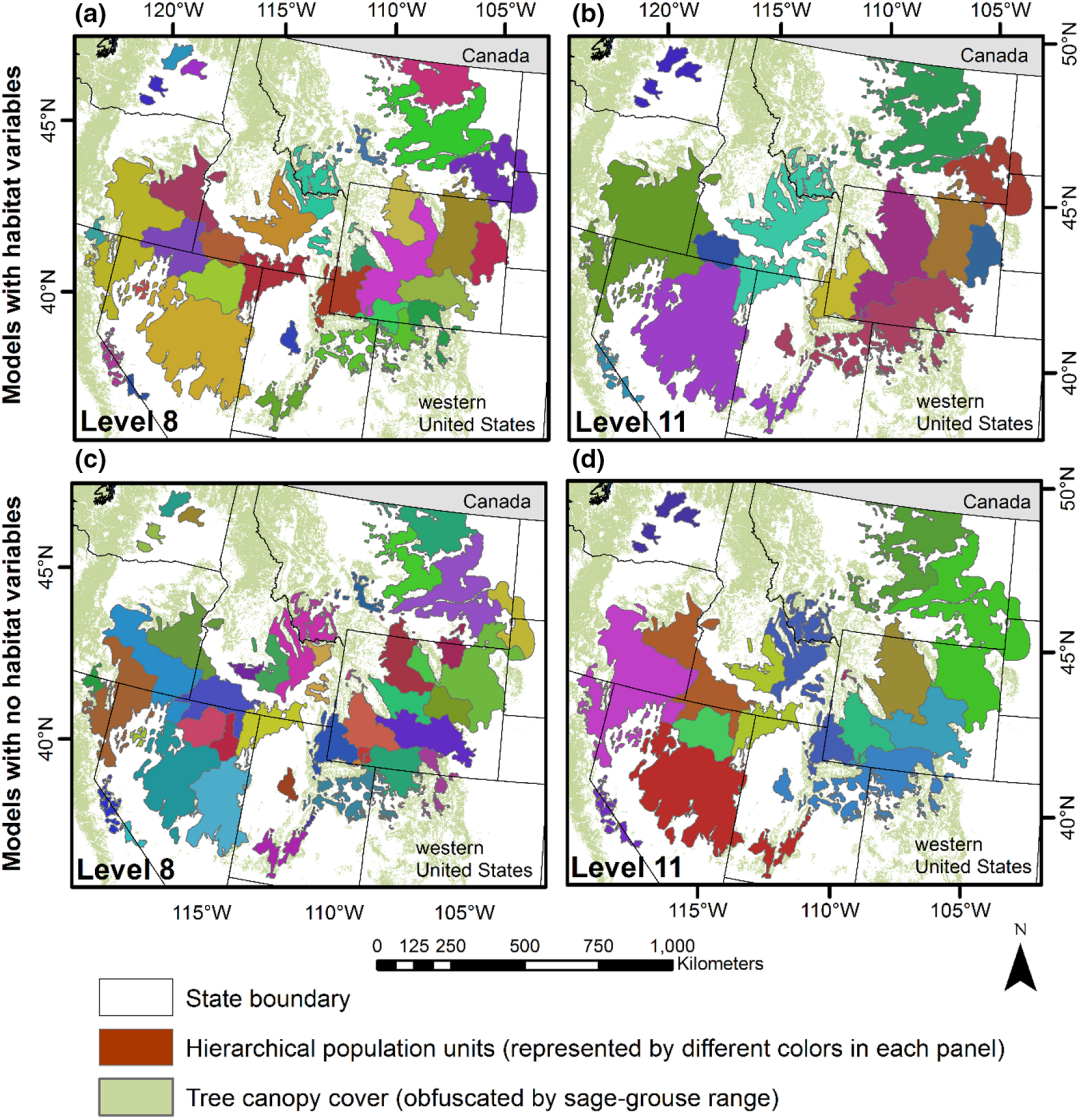
A comparison of hierarchical population levels (cluster levels 8 and 11) of greater sage‐grouse (*Centrocercus urophasianus*) in the western United States informed from sage‐grouse population structure (O'Donnell et al., 2022a) and environment covariates (panels (a) and (b)) or no environment covariates (panels (c) and (d)) using the Spatial “K”luster Analysis by Tree Edge Removal (SKATER) clustering algorithm.

The results from using a non‐habitat covariate to define population units demonstrated the selection of habitat covariates, scales, and aspatial weights played an important role in capturing biological context. All models using a non‐habitat covariate performed significantly worse than models using habitat covariates based on AIC_*c*_ rankings (see supplemental, Table S6). The resulting non‐habitat population units produced 15 cluster levels instead of 13 (obtained when using habitat information), where we defined a prior six desired units before stopping the clustering algorithm. We also observed significant spatial differences among cluster levels using a non‐habitat covariate compared to biologically informed covariates, where we have highlighted cluster levels 8 and 11 to illustrate those differences (Figure 8).

## 4 DISCUSSION

### 4.1 Highlighted findings

We developed a framework (Figure 1) to define hierarchically nested population levels (i.e., cluster levels) to function as biologically relevant management units that could improve wildlife management by supporting analyses of demographic data among different levels of aggregated subpopulation units. The approach best supports species anchored to known sites of fidelity and where monitoring occurs to assess population demographic data using the management units. The hierarchical nature of population units allows researchers to assess population demographic data via cross‐scale linkages (e.g., institutional, jurisdictional, spatial, and temporal scales) as ecological processes potentially affect populations at multiple scales. Therefore, this approach supports distinguishing population dynamics driven by processes occurring at fine scales (small cluster levels) from processes operating at larger scales. The ability to make such distinctions could help inform when management actions might be necessary due to a declining local population or the recognition of when management actions were effective at one or more scales (Coates et al., 2021). Because our approach supports the delineation of approximately closed populations during survey periods, which many population models assume (Iijima, 2020; McDevitt et al., 2021), we have increased confidence in modeling population trends at finer scales. For example, sage‐grouse generally move <15 km (95th percentile; O'Donnell et al., 2019a) between lek sites within and among years (Fremgen et al., 2017; Wann et al., 2019), and therefore, grouping leks at fine scales (e.g., cluster level 2) can account for smaller movement distances and better characterize geographic closure during survey periods (e.g., breeding season). The range‐wide assessment describing the proportion of time (GPS) and use (VHF) locations of individual birds that occurred inside their home cluster demonstrated cluster levels ≥2 captured >92% of time and >95% of use (Figure 7) and therefore these results further demonstrated the utility of using the fine‐scaled units for analyzing demographic data.

Many clustering approaches exist (Wegmann et al., 2021; Xu & Tian, 2015), but they often have limitations with incorporating biological context (e.g., population structure and scale effect). Additionally, we are unaware of any that produce spatially explicit nested clusters and support constraint‐based rules. We believe our approach provides significant biological context and is the only known instance of delineating multi‐scaled population units—an improvement over other methods that can support other species (see Section 4.4: *Applicability to other species* below). The development of population structure tiers included biological inferences of habitat conditions (functional connectivity), dispersal capabilities (potential connectivity), genetic information, and functional processes affecting movements (O'Donnell et al., 2022a). The population structure tiers reflecting connectivity provided constraint‐based rules to SKATER, which could otherwise not be captured in a clustering approach. The clustering algorithm incorporated habitat resources (biotic and abiotic) summarized at multiple spatial scales (30 –6400 m) surrounding each location of site fidelity (functional selection of habitat surrounding breeding sites) to maximize similarities of available habitat resources for grouped lekking sites. We also demonstrated the selection of habitat covariates, scales, and aspatial weights played an important role in capturing biological context (Figure 8), which invariably will differ across a species' range. By incorporating constraint‐based rules (number of sites to group and population structure [connectivity]), multi‐scaled habitat resources, and a bottom‐to‐top (agglomerative) clustering approach, we provided a semi‐supervised approach with significant biological context.

### 4.2 Hierarchical population units as a tool for wildlife managers

The sage‐grouse population units defined here are intended to support multiple research and management applications. Defining subpopulations and multiple scales of population units can improve support for identifying where and when local management is needed when population abundances decline (e.g., Coates et al., 2021), thereby improving management reactions. We did not use demographic data to define population units because it was important to define the clusters independently of abundance data, where the intent is to use the hierarchical population units for analyzing population trends. The smaller‐scaled subpopulation units allow for the evaluation of local population changes relative to populations aggregated at coarse scales (i.e., larger population units). Using different cluster levels crossing jurisdictional boundaries can aid managers in assessing population changes that reflect meaningful aggregations of connected populations. We evaluated the population units based on movements within an individual's biological year because units that capture the majority of movements (e.g., home range) may support modeling changes in vital rates that require open populations (Gardner et al., 2018). Minimally, the subpopulation units presented here capture geographic closure to reduce effects of immigration and emigration during a survey period to minimize bias of detectability when estimating abundance (Gardner et al., 2018; Iijima, 2020).

To use the hierarchically nested population units, the monitoring and collecting of demographic data (e.g., counts of males) needs to occur at locations of site fidelity, allowing for assessing demographic data across the different units of scale. United States state wildlife agencies and their partners have monitored greater sage‐grouse populations since the 1950s by counting males in the early morning on leks where they congregate to attract females for copulation in early spring (Dalke et al., 1963; Patterson, 1952; Wiley, 1973). The standardized monitoring procedures used by state wildlife agencies (Autenrieth et al., 1982) address potential biases of demographic closure, while the subpopulation units developed here address potential biases of geographic closure.

With these products, we can analyze population demographics (e.g., trends with reduced influence from emigration) and assess related mechanisms of change (e.g., populations declining due to functional habitat loss) via cross‐scale linkages, potentially providing greater conservation and adaptive management insight. The analyses of sage‐grouse population trends described in Coates et al. (2021) rely on the hierarchical population units described here and provide an example of how these can be used with sage‐grouse demographic data. Hierarchically nested management units, when juxtaposed with genetic or landscape connectivity data, may also help assess cross‐scale contributions of the units to overall landscape connectivity (Cumming et al., 2022), but assessments of connectivity and demographic data will likely not be meaningful if the units are not biologically relevant. Therefore, there are potentially numerous applications of these population units for future research and management needs.

Wildlife populations that cycle introduce multiple challenges when assessing changes in population dynamics. For example, determining if local populations are changing relative to regional populations, where both may reflect natural cycling, is problematic unless there is a mechanism to assess cross‐scale linkages. Population cycles are commonly characterized as events of high reproductive rates at low population densities, followed by density‐related mortality that reduces and stops the rate of increase, and then a prolonged condition delaying recovery (Myers, 2018). Although predator–prey relationships, disease, food limitations, and extrinsic factors like climate and solar and lunar effects have been studied as potential drivers of population cycles for many species (e.g., snowshoe hares, voles and lemmings, forest Lepidoptera and grouse), there is no consensus on causation (Myers, 2018). Regardless of the mechanisms, cross‐scale assessments of population changes may aid in ascertaining whether population changes are occurring locally (e.g., due to habitat loss) or more closely mimicking regional cyclic patterns. In other words, a local population decline occurring at one rate that is not in sync with a regional population decline potentially signals that something has affected the local population relative to the regional population. For example, effectively managing sage‐grouse is challenging because some populations cycle and the cycling of populations varies spatially and temporally; having the ability to assess trends of demographic data across scales can help differentiate between, for example, local population declines due to local drivers versus a natural cyclic decline captured at larger scales (Coates et al., 2021). The population management units cannot answer why sage‐grouse populations cycle, but they could improve how and when managers react to local population changes.

**FIGURE 9:**
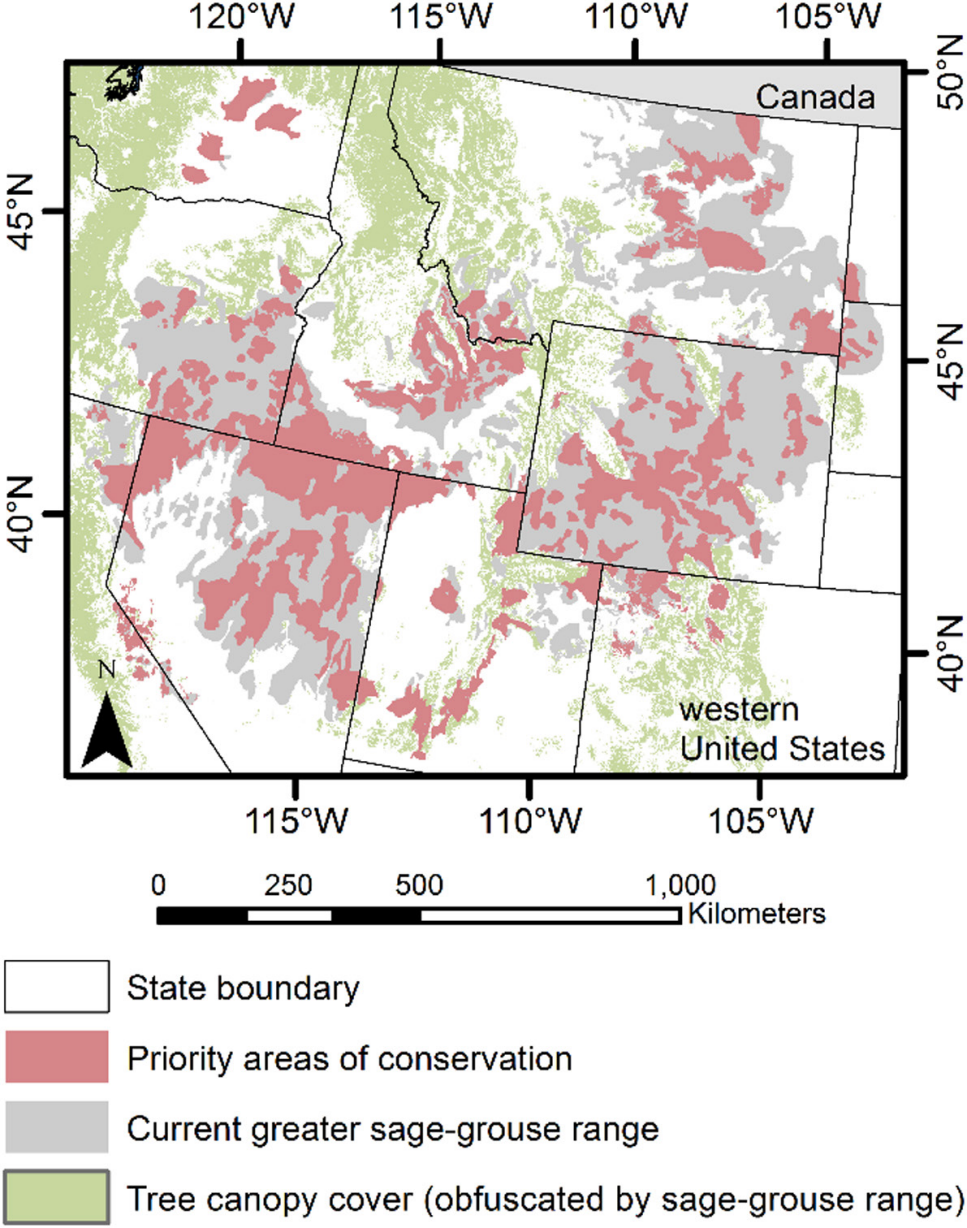
Priority areas of conservation concern for greater sage‐grouse (*Centrocercus urophasianus*) in the western United States identified by state conservation plans or other conservation efforts (U.S. Fish and Wildlife Service, 2013).

The sage‐grouse priority areas of conservation (PAC) represent important habitats identified by state conservation plans and other conservation efforts (U.S. Fish and Wildlife Service, 2013). These single‐scaled conservation areas were designed to inform where threats to sage‐grouse should be minimized to meet objectives in the 2006 Western Association of Fish and Wildlife Agencies Conservation Strategy (Stiver et al., 2006). Each state developed them using slightly different criteria but generally considered breeding bird densities (Doherty et al., 2010), telemetry locations, nesting areas, known distributions of sage‐grouse, and habitat usage (e.g., occupied, suitable, seasonal, nesting and brood rearing, and connectivity areas; U.S. Fish and Wildlife Service, 2013). These areas of conservation concern are critical for wildlife agencies in the management of sage‐grouse populations. They are used by states to focus protection of habitat and management of the core sage‐grouse populations within each state. However, these priority areas are single‐scaled and capture large geographic areas (Figure 9), and therefore, they are difficult to consider when identifying changes in local populations to inform management. States have been counting male sage‐grouse at leks since the 1950s, and these monitoring efforts have significantly increased since the early 2000s (O'Donnell et al., 2021). These demographic data can be used with population models and hierarchical scales for data aggregation to assess local and regional trends that can help improve wildlife management. For example, state wildlife agencies can use population models developed with population units presented here to determine which leks or groups of leks have declining populations within the PACs. We, therefore, believe the population management units presented here can help improve sage‐grouse management by assessing population changes across hierarchical scales of population units.

### 4.3 Caveats

Discrete boundaries are important for managers as they may help increase our understanding of complex ecological processes, but they are challenging to define because they reflect generalities among scales of processes (Patrick & Yuan, 2018; Strayer et al., 2003). We defined cluster boundaries using Thiessen polygons of grouped leks; however, this approach did not account for landscape characteristics occurring along polygon boundaries that may be relevant to sage‐grouse movements, such as ridges, canyons, or vegetation boundaries. Alternatively, we could have chosen hierarchical watersheds, which define areal extents of surface water drainage across six levels (U.S. Geological Survey, 2017), but watersheds do not necessarily reflect how birds use the landscape, especially in a relatively flat sagebrush ecosystem. Watersheds can also have diverse landscape conditions due to terrain aspect and effects of mountains on weather patterns. As another possibility, we could have used spectral clustering of vegetation indices within predefined ecoregions (divisive clustering approach) to produce finer‐scale boundaries (Cheruvelil et al., 2017; Rocchini et al., 2021). However, this likely would not reflect sage‐grouse movements that occur within and between different quality habitats. Our solution of using Thiessen polygons appears appropriate for analyzing aggregated demographic data associated with high‐fidelity sites and provides a means of associating newly located leks to existing clusters.

Modifications to software could be useful for future applications. We encountered occurrences where *skater* did not consistently capture nesting of lek assignments to clusters or did not consistently enforce adjacency. Revising software that enforces rules to ensure adjacency, a hierarchal ruleset, and methods for model selection would improve its utility and usage for similar applications.

### 4.4 Applicability to other species

We summarize data requirements for using our methods to develop biologically relevant and hierarchically nested population units, which may be applied to other species with high‐fidelity to sites. Our workflow requires the use of fidelity sites, habitat covariates, and characteristics of dispersal distances to develop population structures and clusters defining hierarchical population units. Additional to these requirements, population monitoring is necessary to use the population units for wildlife management. We then provide examples of several species in which these methods may be applied based on literature demonstrating site fidelity and adequate monitoring data.

#### 4.4.1 Population structure data requirements

Developing population structures requires three types of datasets, which include: (1) locations of high‐fidelity sites during one or more life stages, (2) environmental data (e.g., terrain, climate, or remotely sensed vegetation) to describe costs of movements for a species (informs resistance surface), and (3) dispersal characteristics. Many species have some level of site fidelity to habitat locations (Gerber et al., 2019) and many species demonstrate population structure (Ryberg et al., 2013). The number of known high‐fidelity sites could affect the development of population structure, but for sage‐grouse, O'Donnell et al. (2022a) demonstrate that delineation of population structure was robust to omission of high‐fidelity sites (e.g., removing 50% and 90% of sites captured 75.5% and 55.6% of population structure, respectively). Environmental data may include habitat preference/avoidance, barriers, and similar features that can describe how a species uses habitat between locations of site fidelity. Although dispersal data describing distances of species' movements are not required, this information can help inform the decomposition of a fully connected population structure into population structure tiers. The tiers are used to provide greater biological context during the clustering and formation of different cluster levels. One could also develop a reasonable gradient of distances traveled if minimum and maximum movement distance are known, and there is an understanding of a species' ability to move within a certain time period.

#### 4.4.2 Clustering data requirements

The clustering of site fidelity locations relies on data resulting from population structures (Section 4.4.1) and environmental data (e.g., terrain, climate, or remotely sensed vegetation) describing a species' habitat at varying spatial scales. The number of known fidelity sites could affect the number of cluster levels depending on how users define the number of features to group per cluster level; a greater number of cluster levels can increase the number of cross‐scale linkages for investigating population changes. The number of fidelity sites could also affect the size of population units per cluster level; smaller population units will better support habitat/wildlife management at local scales. However, the lack of such data should not preclude users from proceeding, as programs and agencies can revise the population units as more information is collected.

#### 4.4.3 Species examples

Long‐term monitoring programs are scarce despite their importance (e.g., Robinson et al., 2014; Witmer, 2005). One study found long‐term monitoring programs primarily focused on birds, mammals, and plants, which on average lasted for 21, 13, and 10 years for high, middle, and low‐income countries, respectively (Moussy et al., 2022). An important consideration is the appropriate duration of monitoring needed to detect significant trends, which has been shown to vary by biological classes (10–20 years; White, 2019); however, for populations that cycle, monitoring will need to include multiple nadirs. When such programs exist, standardized databases for species occurring across jurisdictional boundaries are similarly scarce due to lack of cross‐scale (institutional) coordination (O'Donnell et al., 2021; Urbano et al., 2010). The tracking of such efforts is also lacking, further presenting problems for programs like the United States ESA (Evansen et al., 2021). Monitoring methods should follow strategies appropriate for the species and that consider population closure or requirements of models used to estimate population abundances and trends. Also, the collection and use of harvest data from game animals to estimate population size and rates of population change may lead to large biases of population estimates and therefore using counting and capture‐recapture methods is also recommended (Fukasawa et al. 2020).

Examples exist of wildlife species with site fidelity to locations where population monitoring occurs, such as breeding, den, or calving locations. The most obvious application would be applying our methods to other lekking species (e.g., lesser prairie chicken [*Tympanuchus pallidicinctus]*), of which there are many. However, other species with different types of site fidelity would be appropriate. For example, the trans‐migratory booted eagle (*Hieraaetus pennatus*) found in Europe and Asia is territorial and return to nesting sites after migrating south during winter. Polar bears (*Ursus maritimus*) generally return to the same denning areas, which are more frequently located along seashore embankments. For these species or others, the process of defining population units includes generating spatial population structures from sites of high‐fidelity (e.g., lekking, nesting, or denning sites) and then clustering the sites into population units using the population structure and habitat covariates to further help characterize subpopulation units. Table 1 provides a list of these three species (lesser prairie chicken, booted eagle, and polar bears) that demonstrate site fidelity, monitoring, and each with numerous studies describing territories, subpopulations, dispersal distances/home ranges, and habitat requirements.

## 5 CONCLUSIONS

We presented a framework for defining biologically relevant population management units of hierarchically nested population levels to assist wildlife managers with monitoring population changes and targeting wildlife and habitat management actions at appropriate scales. Many species are spatially structured (Ryberg et al., 2013) and have fidelity to sites during some portion of their lifecycle (Gerber et al., 2019), and therefore, we expect our approach to work for many other species beyond those that lek. The benefit of using biologically informed population units is that changes in population demographics are relative to available resources and functional use. More so, these population units could support cross‐scale linkages of aggregated demographic data, thereby supporting the investigation of ecological processes affecting population changes at different scales while accounting for the relative availability of resources. Our approach to defining biologically relevant and hierarchically nested population management units provides wildlife managers a tool for informing habitat management and adaptive management and also provides researchers a tool for investigating where and when populations respond to changes occurring on the landscape.

## AUTHOR CONTRIBUTIONS

**Michael S. O'Donnell:** Conceptualization (equal); data curation (equal); formal analysis (equal); funding acquisition (equal); investigation (equal); methodology (equal); project administration (equal); software (equal); validation (equal); visualization (equal); writing – original draft (equal); writing – review and editing (equal). **David R. Edmunds:** Conceptualization (equal); data curation (equal); funding acquisition (equal); investigation (equal); methodology (equal); project administration (equal); validation (equal); writing – original draft (equal); writing – review and editing (equal). **Cameron L. Aldridge:** Conceptualization (equal); data curation (equal); funding acquisition (equal); investigation (equal); project administration (equal); supervision (equal); writing – original draft (equal); writing – review and editing (equal). **Julie A. Heinrichs:** Conceptualization (equal); writing – original draft (equal); writing – review and editing (equal). **Adrian P. Monroe:** Funding acquisition (equal); investigation (equal); writing – review and editing (equal). **Peter S. Coates:** Funding acquisition (equal); investigation (equal); writing – review and editing (equal). **Brian G. Prochazka:** Investigation (equal); validation (equal); writing – review and editing (equal). **Steve E. Hanser:** Data curation (equal); funding acquisition (equal); investigation (equal); project administration (equal); writing – review and editing (equal). **Lief A. Wiechman:** Data curation (equal); funding acquisition (equal); investigation (equal); project administration (equal); writing – review and editing (equal).

## CONFLICT OF INTEREST

Authors certify that they have no affiliations with or involvement in any organization or entity with any financial interest or otherwise that might be perceived as influencing objectivity of this research.

## Supporting information


Appendix S1

## Data Availability

Software (*popcluster* v.2.0) supporting this project (O'Donnell et al., 2022c; https://doi.org/10.5066/P9X68ADU) and population management units data derived for sage‐grouse (O'Donnell et al., 2022b; https://doi.org/10.5066/P9D1K0LX) are freely available to the public. The greater sage‐grouse lek data have limited availability owing to unique restrictions held by each state due to the sensitivity of the species. Contact Technical Team for more information (see *Acknowledgments*). Greater sage‐grouse location data used for evaluating population units are not available or have limited availability owing to restrictions (research proprietary interest and sensitivity of species).
