# Supplementary material for: Defining biologically relevant and hierarchically nested population units to inform wildlife management
Source: Ecol Evol. 2022 Nov 30;12(12):e9565. doi: 10.1002/ece3.9565 (PMC9712811; doi:10.1002/ece3.9565)
Supplement: Supplementary file 1 — Appendix S1 [file ECE3-12-e9565-s001.docx]

**Supplemental**

**Title**: Defining biologically relevant and hierarchically nested population units to inform wildlife management

**Authors**: Michael S. O'Donnell, David R. Edmunds, Cameron L. Aldridge, Julie A. Heinrichs, Adrian P. Monroe, Peter S. Coates, Brian G. Prochazka, Steve E. Hanser, and Lief A. Wiechman

*Any use of trade, firm, or product names is for descriptive purposes only and does not imply endorsement by the U.S. Government.*

**Table of Contents**

[S1. Enhancements to pilot analyses 2](#_Toc117929365)

[S2. Covariate development 4](#_Toc117929366)

[S3. Results of greater sage-grouse, hierarchical population units 11](#_Toc117929367)

[S4. Evaluation of greater sage-grouse, hierarchical population units 31](#_Toc117929368)

[Literature Cited 42](#_Toc117929369)

# S1. Enhancements to pilot analyses

Our pilot study explored the development of a hierarchical population monitoring framework for greater sage-grouse (*Centrocercus urophasianus; hereafter, sage-grouse*) in two disparate states, Nevada and Wyoming (O'Donnell et al., 2019). Here, we briefly describe changes implemented in the current study relative to the pilot study.

1. Lek (high fidelity breeding sites) database (O'Donnell et al., 2021):
2. We standardized state lek count databases by fixing typographical errors, assigning standard columns names, standardizing how leks are classified, among other important features to compile into a range-wide database. For example, we developed a standardized definition of conservation status for identifying active leks across the sage-grouse range. The standardized definition, which was not used in the pilot, affects the inclusion of leks in the analyses. The pilot study used 909 leks in Nevada and 2,020 leks in Wyoming based on state definitions of active leks, and the current study used 882 leks in Nevada and 1,637 leks in Wyoming based on the standardized database (definition approved by the Technical Team).
3. We included significant improvements to lek data by addressing typographical errors and inconsistent treatment of satellite leks. Satellite leks are periphery to main leks and normally have few males; however, the number of males waxes and wanes with population cycles (e.g., increased population sizes on main leks resulted in more males attending satellite leks). When lek locations within the state databases occurred within 500 m of another lek, we aggregated the counts and selected the locations that had the greatest median male count for the last 10-years. If states attributed a lek as being a satellite, we used the same rules and ignored the classification. All approaches were discussed with each of the 11 western state lead biologists.
4. Population structure (O'Donnell et al., 2022a):
5. Based on a literature review, we increased the number of documented dispersal capabilities for sage-grouse across the range, which affected the number of hierarchical tiers of the population structure used to inform the clustering analyses. The population structure developed here used additional dispersal distances, allowing for a greater number of informed population structure tires than the pilot study.
6. We included high traffic volume data to attenuate population movements, informing the hierarchical tiers of the population structure, which was only considered for I-80 in southern Wyoming in the Pilot study. This approach increased the accuracy and standardization of how roads affected the frequency of sage-grouse movements.
7. We revised the resistance surface to better represent biologically relevant habitat characteristics (e.g., sagebrush cover, terrain/vector ruggedness, and forest canopy cover) and methods informing functional connectivity (i.e., scales summarizing surrounding habitat characteristics). The pilot study used a digital elevation model as the resistance surface, and O'Donnell et al. (2022a) illustrate the differences between using the two different surfaces across the sage-grouse range with respect to changes in population structure.
8. We used centrality measures to assess the population structure relative to high bird densities and genetic flow patterns. These measures developed in O'Donnell et al. (2022a) provided substantial support, which was not explored in O'Donnell et al. (2019).
9. We evaluated the sensitivity of developing the population structure by incrementally withholding data (O'Donnell et al., 2022a). This sensitivity analysis demonstrated the robustness of population structure concepts for sage-grouse.
10. Clustering:
11. We modified the range of leks grouped at each cluster level to handle additional leks and avoid developing too many cluster levels (e.g., <15 cluster levels). In other words, we increased the number of leks to group per cluster level more quickly.
12. We increased the number of incremental scales assessed for summarizing covariates between 500 m and 6,400 m. The pilot included radii of 500 m, 1,000 m, 1,500 m, 3,200 m, and 6,400 m. The current study includes radii of 500 m, 1,000 m, 1,500 m, 2,200 m, 3,000 m, 4,700 m, and 6,400 m.
13. We used additional habitat covariates, including heat load index, weighted compound topographic index, topographic position index, and derived sagebrush/shrub cover data.
14. We included all known sage-grouse leks across the species' range instead of limiting them to two disparate state boundaries (Nevada and Wyoming), as done in the pilot. This eliminated issues of producing incomplete population structures and clusters constrained by artificial state boundaries. Restricting lek data to Nevada and Wyoming affects the population structure and clustering of leks into population units.
15. We changed methods for covariate selection when using the last least-cost path minimum spanning tree (population structure LCP-MST-5; described in sections Population structure overview and Developing population units of main article). Instead of using the best model from the last LCP-MST for all cluster levels beyond the last population structure tier (i.e., LCP-MST-5), we continued exploring additional covariates for each odd cluster level. We modified this approach to better support a parsimonious clustering framework and ensured better identification of clusters. The pilot study stopped evaluating covariates after the last population structure was incorporated into the workflow; however, the pilot had fewer cluster levels due to the fewer number of leks.
16. Consultation with state lead experts from the Greater Sage-Grouse Technical Team (hereafter referred to as the Technical Team) of the Western Association of Fish and Wildlife Agencies:
17. The Technical Team assisted with developing and standardizing the sage-grouse database and conservation status (e.g., active leks). We did not work with the Technical Team during the pilot study.
18. The Technical Team provided feedback on population structure (i.e., where connectivity required modification specific to team member’s state; see O'Donnell et al., 2022a). We did not work with the Technical Team during the pilot study.
19. The Technical Team provided feedback on the hierarchically nested population units. The suggested adjustments required changes to the hierarchical population tiers (see O'Donnell et al., 2022a). We did not work with the Technical Team during the pilot study.
20. Evaluation:
21. We used many independent research studies providing location data from global positioning systems (GPS) and very-high frequency (VHF) devices across the species’ range to identify how well each cluster level captured closed population units. Pilot study included GPS data in Nevada only (unique birds*=*247; use locations=58,887), where this study included GPS (unique birds*=*1,551; use locations=1,685,443) and VHF (unique birds*=*1,270; use locations=31,731) data in Nevada, Oregon, California, Washington, Idaho, Colorado, South Dakota, and Wyoming.
22. We used the dynamic Brownian bridge movement model (dBBMM) with GPS data and assessed VHF locations. These assessments examined the amount of time (GPS) and proportion of observations (VHF) occurring for individuals in their home range during a biological year. Here we defined the home range for individual birds using a biological year (March to March) instead of a calendar, as defined in the pilot study.

# S2. Covariate development

We evaluated numerous candidate habitat covariates at multiple spatial scales for inclusion in the Spatial “K”luster Analysis by Tree Edge Removal (R. AssunÇão, Krainski, Ribeiro, & Rodrigues, 2012; SKATER; R. M. AssunÇão, Neves, Câmara, & Da Costa Freitas, 2006) clustering algorithm. These data sources are referenced in Table S1, and the different spatial scales used to summarize habitat surrounding sage-grouse leks are specified in Table S2. We also provide definitions and interpretations for derived data not explained in Table S1, including the following: hydrologically corrected digital elevation model, compound topographic index weighted by annual precipitation, heat load index, topographic position index, vector ruggedness measure, and four bioclimatic variables describing average climate conditions. Figure S1 highlights the data inputs used to define population structure (O'Donnell et al., 2022a) and the clustering of lek locations. Each covariate data set and combinations of scales (i.e., summarizing habitat surrounding leks) were candidates for the clustering algorithm. The best model (one or more covariates, one or more scales of different covariates, and spatial weight [Euclidean or Mahalanobis]) was selected using the Akaike information criterion corrected for small sample sizes (AICc; Burnham, Anderson, & Huyvaert, 2011).

*Hydrologically corrected digital elevation model*: We created a 10-meter digital elevation model (DEM) of the western United States by mosaicking elevation tiles (U.S. Geological Survey, 2018). The DEM was hydrologically corrected to reduce the number of elevation anomalies using the optimized pit removal software (Soille, 2004; v. 1.5.1). Peaks and troughs of two to five meters are rare within 10-m DEMs and generally reflect errors (Tarboton, Bras, & Rodriguez-Iturbe, 1991). Correcting this noise improves flow accumulation analyses, which we used in different terrain-derived indices. We used these corrected results for all derived terrain covariates described below.

*Compound topographic index weighted by annual precipitation*: The compound topographic index (CTI) is a steady-state wetness index calculated from slope and upstream contributing area (Gessler, Moore, McKenzie, & Ryan, 1995). The index is calculated for 3-cells by 3-cells where flatter areas have larger values (typically not informative), and smaller catchments with steep slopes have low values. The index was calculated using the following equation: $\ln\left( (\left( flow accumulation+1 \right)* {pixel area}_{meters} \right))/\tan({slope}_{radians})$.The index is intended as a surrogate for soil moisture and vegetative productivity; however, the index is problematic when applied to large geographic extents where precipitation differs because it only considers terrain concavity. Therefore, we defined flow accumulation by weighting with 1981 – 2010 total precipitation normal climate data (Prism Climate Group, 2015), allowing comparable values of CTI across large geographic space. We then applied moving windows with multiple radii to reflect different functional scales and summary statistics.

*Heat load index*: The heat load index (HLI) identifies the potential annual direct incident radiation suitable for our latitudes of 30 – 60 degrees North (regression equation three [R2 = 0.983]; McCune & Keon, 2002). The equation includes latitude, slope, and aspect, where the coolest slopes occur on northeastern aspects and the warmest slopes on southwestern aspects (northern hemisphere). The values can range from 0 (little to no variation in the terrain and therefore coolest) to 1 (significant variation and therefore hottest), but most natural terrain have values distributed below 0.5. The index does not account for cloud cover, regional differences in the atmospheric coefficient, or shading due to topography. We then applied moving windows with multiple radii to reflect different functional scales and summary statistics.

*Topographic position index*: The topographic position index (TPI) is a relative measure of whether a particular target cell is higher or lower than surrounding cells (Weiss, 2001). The algorithm calculates the center cell elevation value of a kernel minus the average elevation of the neighborhood cells, which is achieved using a neighborhood annulus (i.e., donut) moving window. Positive TPI values indicate the center cell is higher than surrounding cells, while negative values indicate the center cell is lower than surrounding cells. The magnitude of the TPI value explains where on a slope the pixel resides relative to its neighbors. Larger neighborhoods will capture broader landscape characteristics, while smaller neighborhoods will describe characteristics of small areas (large-scale). We then applied moving windows with multiple radii to reflect different functional scales and summary statistics.

*Vector ruggedness measure*: We developed a vector ruggedness measure (VRM), defined by Sappington, Longshore, and Thompson (2007), using multiple radii to capture a gradient of terrain features depicting local and small-scale terrain features to larger mountainous features. The VRM is like other forms of calculating terrain ruggedness, but it incorporates slope and aspect, unlike other methods. Different radii were incorporated into the calculation of this index, and therefore applying summary statistics using moving windows was unnecessary.

*Bioclimatic variables*: We derived bioclimatic variables representing biologically relevant climate indices using 1981 – 2010 climate normal data (Prism Climate Group, 2015) and methods outlined in O'Donnell and Ignizio (2012). We considered annual mean temperature (°C), mean temperature (°C) of wettest season, mean temperature (°C) of warmest quarter, annual precipitation totals (mm), and precipitation seasonality. We applied moving windows with multiple radii to reflect different functional scales and summary statistics. We did not include the 500-meter radius because the source resolution of the climate data was 800 m (resampled to 30 meters).

*Greater sage-grouse current range*: The greater sage-grouse range boundary used for defining extents of hierarchically nested and biologically relevant range-wide monitoring framework was updated from the U.S. Fish and Wildlife Service data published in 2015, which was a collection of state wildlife agency-defined boundaries. We updated the original range data with input from each state. However, we required the new range to include all lek locations (e.g., historic leks), and therefore the revised boundary does not necessarily reflect rangelands currently managed by states.

**Table S1**. Spatial datasets were acquired to define and evaluate greater sage-grouse (*Centrocercus urophasianus*) population units in the western United States. All analyses of these data relied on Conical Albers Equal Area with standard parallels appropriate for the contiguous United States and a 1984 World Geodetic System datum. Not applicable (NA) denotes data derived for the current study and not downloaded.

| **Category** | **Source** | **Description** | **Source scale/ resolution** | **Geographic extent** | **Ground date** | **Access date** |
| --- | --- | --- | --- | --- | --- | --- |
| Sagebrush biome vegetation | Rigge et al. (2020); Xian, Homer, Rigge, Shi, and Meyer (2015), (U.S. Geological Survey & Homer, 2019) | Percent cover: bare ground, big sagebrush, herbaceous, all sagebrush, all shrub, and perennial grass; Height: sagebrush and shrub | 30 m | Sagebrush biome | 2016 | ^1^Shared via communication 11/14/2017 |
|  | Derived from Rigge et al. (2020); Xian et al. (2015) | Non-big sagebrush (pos) and non-sagebrush shrub (pnss) | 30 m | Sagebrush biome | 2016 |  |
| Terrain | U.S. Geological Survey (2018) | Digital elevation model (DEM) | 10 m | Contiguous U.S. | Unknown | 04/04/2018 |
|  | Derived from DEM | Vector ruggedness index | 10 m | Sagebrush biome | Unknown | NA |
|  | Derived from DEM and PRISM | Compound topographic index weighted by annual precipitation | 10 m | Sagebrush biome | Unknown | NA |
|  | Derived from DEM | Heat load index | 10 m | Sagebrush biome | Unknown | NA |
|  | Derived from DEM | Topographic position index | 10 m | Sagebrush biome | Unknown | NA |
| Climate | Prism Climate Group (2015) | PRISM climate normal (1981-2010) | 800 m | Contiguous U.S. | 1981 – 2010 | 12/20/2017 |
|  | Derived from PRISM | Bio1: The annual mean temperature (°C) | 800 m | Contiguous U.S. | 1981 – 2010 | 12/20/2017 |
|  | Derived from PRISM | Bio8: Mean temperature (°C) of wettest season | 800 m | Contiguous U.S. | 1981 – 2010 | 12/20/2017 |
|  | Derived from PRISM | Bio10: Mean temperature (°C) of warmest quarter | 800 m | Contiguous U.S. | 1981 – 2010 | 12/20/2017 |
|  | Derived from PRISM | Bio12: Annual precipitation totals (mm) | 800 m | Contiguous U.S. | 1981 – 2010 | 12/20/2017 |
|  | Derived from PRISM | Bio15: Precipitation seasonality (coefficient of variation) | 800 m | Contiguous U.S. | 1981 – 2010 | 12/20/2017 |
| Population structures | (O'Donnell et al., 2022b) | Greater sage-grouse population structures | Not applicable | Greater sage-grouse extent (sagebrush biome) | Not applicable | Not applicable |
| Greater sage-grouse | U.S. Fish and Wildlife Service (2014a) | Greater sage-grouse management zones developed by Western Association of Fish and Wildlife Agencies | Est. 1:1,000,000 | Greater sage-grouse extent (sagebrush biome) | 2015 | 06/24/2021 |
| Greater sage-grouse | U.S. Fish and Wildlife Service (2015) | Greater sage-grouse current range originally developed by Western Association of Fish and Wildlife Agencies and revised here | Est. 1:500,000 | Greater sage-grouse extent (sagebrush biome) | 2015 | 06/24/2021 |
| Greater sage-grouse | U.S. Fish and Wildlife Service (2014b) | U.S. Fish and Wildlife Service priority areas of conservation | Est. 1:500,000 | Greater sage-grouse extent (sagebrush biome) | 2015 | 06/24/2021 |

^1^A pre-release version of these data was provided for this project (C. Homer, U.S. Geological Survey retiree, written commun, 11/14/2017). A later version, where ecoregions across the sagebrush were merged, was made public (https://doi.org/10.5066/P9LTU2QM; Xian et al. 2015, Rigge et al. 2020).

**Table S2.** Spatial scales were assessed for candidate covariates (spatial datasets) and used to define greater sage-grouse (*Centrocercus urophasianus*), hierarchical population units in the western United States. Not applicable (NA) denotes data not processed at a given scale, and ‘x’ represents data processed at a given scale (“cells” indicate the number of 10-meter cells used to represent the radius; radius expressed in meters). After deriving moving windows, we resampled 10-meter terrain indices to 30 meters. The compound topographic index and topographic index included three additional scales (respectively, 30, 100, 250 meters) because we retained the original 10-meter spatial resolution when deriving these metrics in order to capture the appropriate concavity/convexness of the terrain.

| **Covariate/Dataset** | **Moving window dimensions (cells)** | | | |  | **Moving window dimensions (radius meters)** | | | | | | |
| --- | --- | --- | --- | --- | --- | --- | --- | --- | --- | --- | --- | --- |
|  | **1** | **3** | **10** | **25** |  | **500** | **1,000** | **1,500** | **2,200** | **3,000** | **4,700** | **6,400** |
| Digital elevation model (DEM) | x | NA | NA | NA |  | NA | NA | NA | NA | NA | NA | NA |
| Hydrologically corrected DEM | x | NA | NA | NA |  | x | x | x | x | x | x | x |
| Compound topographic index weighted by annual precipitation | NA | x | NA | NA |  | x | x | x | x | x | x | x |
| Heat load index | x | NA | NA | NA |  | x | x | x | x | x | x | x |
| Topographic position index | NA | x | x | x |  | x | x | x | x | x | x | x |
| Vector ruggedness index | NA | NA | NA | NA |  | x | x | x | x | x | x | x |
| Percent cover bare ground | x | NA | NA | NA |  | x | x | x | x | x | x | x |
| Percent cover big sagebrush | x | NA | NA | NA |  | x | x | x | x | x | x | x |
| Percent cover herbaceous | x | NA | NA | NA |  | x | x | x | x | x | x | x |
| Percent cover all sagebrush | x | NA | NA | NA |  | x | x | x | x | x | x | x |
| Percent cover all shrub | x | NA | NA | NA |  | x | x | x | x | x | x | x |
| Sagebrush height | x | NA | NA | NA |  | x | x | x | x | x | x | x |
| Shrub height | x | NA | NA | NA |  | x | x | x | x | x | x | x |
| Percent non-big (other) sagebrush (pos) | x | NA | NA | NA |  | x | x | x | x | x | x | x |
| Percent non-sagebrush shrub (pnss) | x | NA | NA | NA |  | x | x | x | x | x | x | x |
| Percent perennial grass | x | NA | NA | NA |  | x | x | x | x | x | x | x |
| Bio1: The annual mean temperature (°C) | NA | NA | NA | NA |  | NA | x | x | x | x | x | x |
| Bio8: Mean temperature (°C) of wettest season | NA | NA | NA | NA |  | NA | x | x | x | x | x | x |
| Bio10: Mean temperature (°C) of warmest quarter | NA | NA | NA | NA |  | NA | x | x | x | x | x | x |
| Bio12: Annual precipitation totals | NA | NA | NA | NA |  | NA | x | x | x | x | x | x |
| Bio15: Precipitation seasonality | NA | NA | NA | NA |  | NA | x | x | x | x | x | x |


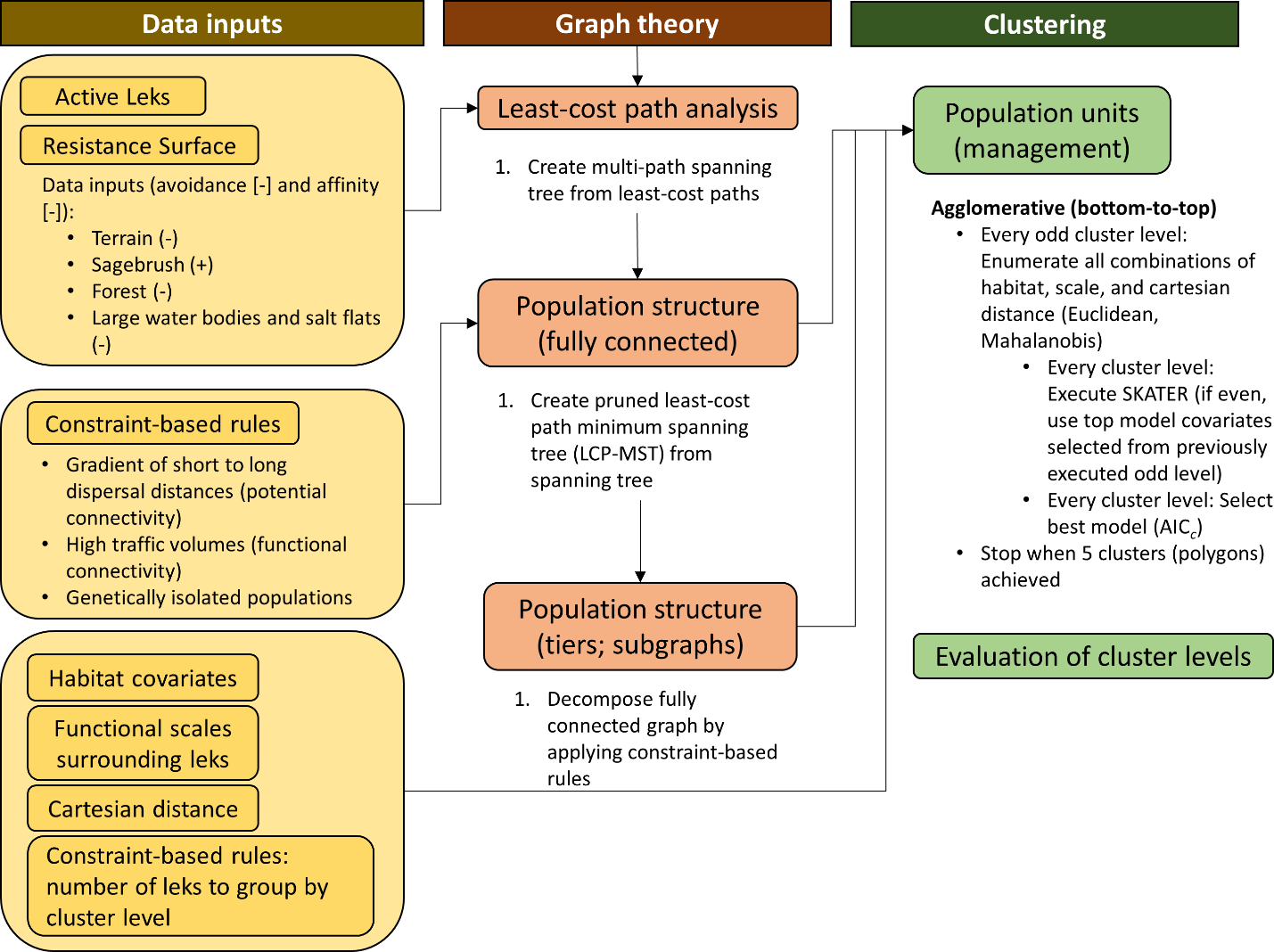


**Figure S1**. Description of spatial data inputs used for identifying population structure with graph theory and Spatial “K”luster Analysis by Tree Edge Removal (SKATER) clustering algorithm of greater sage-grouse (*Centrocercus urophasianus*) in the western United States. We used the Akaike information criterion corrected for small sample sizes (AIC*_c_*) to select the best model produced by the clustering algorithm. The clustering algorithm was applied to each subgraph of the population structure and assessed one or more candidate habitat covariates, scales/radii surrounding leks, and spatial weight (cartesian distance measurement; Euclidean and Mahalanobis).

**Table S3**. The number of greater sage-grouse (*Centrocercus urophasianus*) leks (breeding display grounds) recommended to the clustering algorithm (Spatial “K”luster Analysis by Tree Edge Removal) as a constraint-based rule during the identification of hierarchical population units in the western United States. This constraint-based rule will help ensure spatially balanced population units.

| **Cluster level** | **Minimum number of leks** | **Maximum number of leks** | **Mean leks per cluster** |
| --- | --- | --- | --- |
| 1 (smallest areal units) | 10 | 20 | 10 |
| 2 | 20 | 30 | 10 |
| 3 | 30 | 45 | 15 |
| 4 | 45 | 65 | 20 |
| 5 | 65 | 90 | 25 |
| 6 | 90 | 120 | 30 |
| 7 | 120 | 155 | 35 |
| 8 | 155 | 205 | 50 |
| 9 | 205 | 245 | 40 |
| 10 | 245 | 305 | 60 |
| 11 | 305 | 445 | 140 |
| 12 | 445 | 705 | 260 |
| 13 (largest areal units) | 705 | 1245 | 540 |

# S3. Results of greater sage-grouse, hierarchical population units

Our approach resulted in 13 cluster levels. Table S4 summarizes the number of polygons (clusters), subgraphs (i.e., subpopulations based on hierarchical population structures), and detached leks per cluster level. Table S5 provides a description of the number of clusters (population units) and range of areas captured by the different population units per cluster level. Detached leks are not connected to a subgraph because a connectivity rule prevented its affiliation with surrounding leks (O'Donnell et al., 2022a). Figures S2 – S6 and corresponding Table S6 describe each subgraph that included a sufficient number of leks for clustering (i.e., greater than maximum number of leks in Table S3 at respective cluster level). Table S6 consists of the top three habitat covariates identified using Akaike information criterion corrected for small sample sizes (AIC*_c_*). Each top model includes the best combination of a habitat covariate, scale (i.e., area surrounding a lek), summary statistic for a habitat covariate (coefficient of variation [CV] and mean), and spatial weight. We calculated the proportion of leks contributing to covariate, scale, summary statistic, and distance measure (Figures S5 – S9) per least-cost path minimum spanning tree (LCP-MST). The relative importance of selected covariates (Figure S5 – S6), scale (Figure S7), summary statistic (Figure S8), and distance measure (Figure S9) selected in the top model describe the breakdown by population structure tier.

**Table S4**. Summary of management units (cluster polygons), subgraphs of the population structure derived from a least-cost path minimum spanning tree (LCP-MST), and detached leks (breeding display ground) per cluster level for greater sage-grouse (*Centrocercus urophasianus*) in the western United States. Detached leks are nodes excluded from graphs (graph theory) of the hierarchical population structure (total of five tiers).

|  | **Cluster level** | | | | | | | | | | | | |
| --- | --- | --- | --- | --- | --- | --- | --- | --- | --- | --- | --- | --- | --- |
| **Quantity of item per cluster level** | **1** | **2** | **3** | **4** | **5** | **6** | **7** | **8** | **9** | **10** | **11** | **12** | **13** |
| Least-cost path minimum spanning tree (LCP-MST) | 1 | 1 | 2 | 2 | 3 | 3 | 4 | 4 | 5 | 5 | 5 | 5 | 5 |
| Polygons (clusters) | 650 | 472 | 193 | 147 | 77 | 70 | 39 | 34 | 20 | 17 | 14 | 10 | 6 |
| Subgraphs | 335 | 335 | 68 | 68 | 39 | 39 | 14 | 14 | 4 | 4 | 4 | 4 | 4 |
| Detached leks | 122 | 122 | 12 | 12 | 4 | 4 | 1 | 1 | 0 | 0 | 0 | 0 | 0 |

**Table S5.** Description of cluster-level population units (number of cluster polygons, minimum and maximum area, and mean area per cluster level) developed for greater sage-grouse (*Centrocercus urophasianus*) in the western United States.

| **Cluster level** | **Cluster count** | **Minimum area (km^2^)** | **Maximum area (km^2^)** | **Mean area (km^2^)** |
| --- | --- | --- | --- | --- |
| grsg_cluster_lv1 | 650 | 1.1 | 7,163.7 | 1,098.2 |
| grsg_cluster_lv2 | 472 | 1.1 | 7,831.7 | 1,505.9 |
| grsg_cluster_lv3 | 193 | 70.2 | 14,854.3 | 3,717.8 |
| grsg_cluster_lv4 | 147 | 70.2 | 28,377.1 | 4,889.1 |
| grsg_cluster_lv5 | 77 | 93.3 | 41,799.1 | 9,270.3 |
| grsg_cluster_lv6 | 70 | 93.3 | 51,962.8 | 10,197.3 |
| grsg_cluster_lv7 | 39 | 667.3 | 67,684.4 | 18,302.8 |
| grsg_cluster_lv8 | 34 | 667.3 | 93,761.9 | 20,994.4 |
| grsg_cluster_lv9 | 20 | 667.3 | 103,568.1 | 35,690.5 |
| grsg_cluster_lv10 | 17 | 667.3 | 103,568.1 | 41,988.9 |
| grsg_cluster_lv11 | 14 | 667.3 | 129,068.8 | 50,986.5 |
| grsg_cluster_lv12 | 10 | 667.3 | 143,036.3 | 71,381.1 |
| grsg_cluster_lv13 | 6 | 667.3 | 346,272.0 | 118,968.4 |


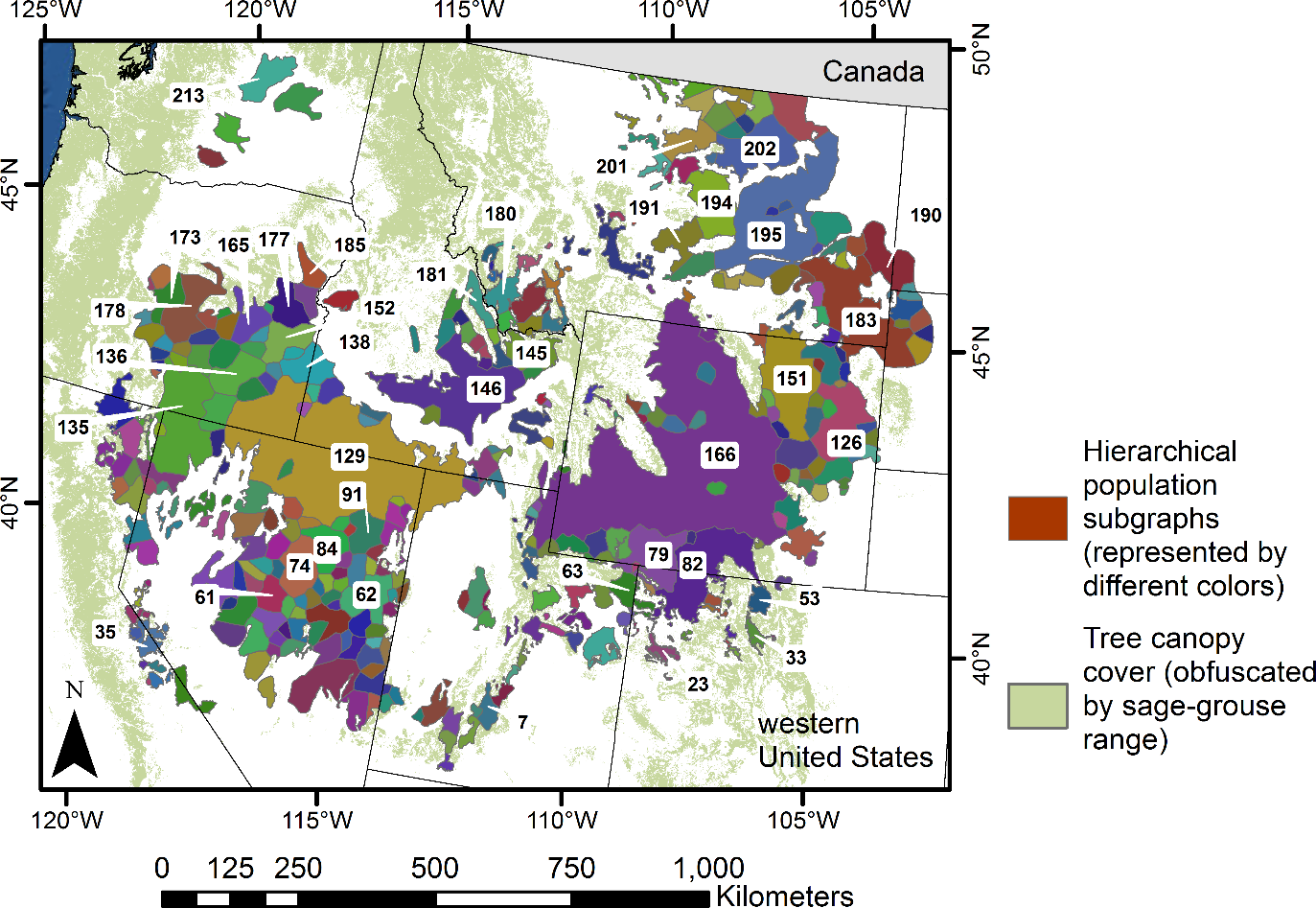


**Figure S2**. Thiessen polygons of greater sage-grouse (*Centrocercus urophasianus*) subpopulations (subgraphs) in the western United States, as defined by our hierarchal population structure (O'Donnell et al., 2022a). Subgraphs informed clustering of sage-grouse lek locations (breeding display grounds) into hierarchical cluster levels. Here, we show polygons of hierarchical population structure tier one (used for cluster levels one and two; finest scales). The numeric labels of subgraph identifiers correspond to the column “Subgraph ID” in Table S6. Unlabeled polygons lacked enough leks for clustering (i.e., less than the maximum number of leks in Table S3 or detached leks in Table S4), resulting in no model in Table S6.

**
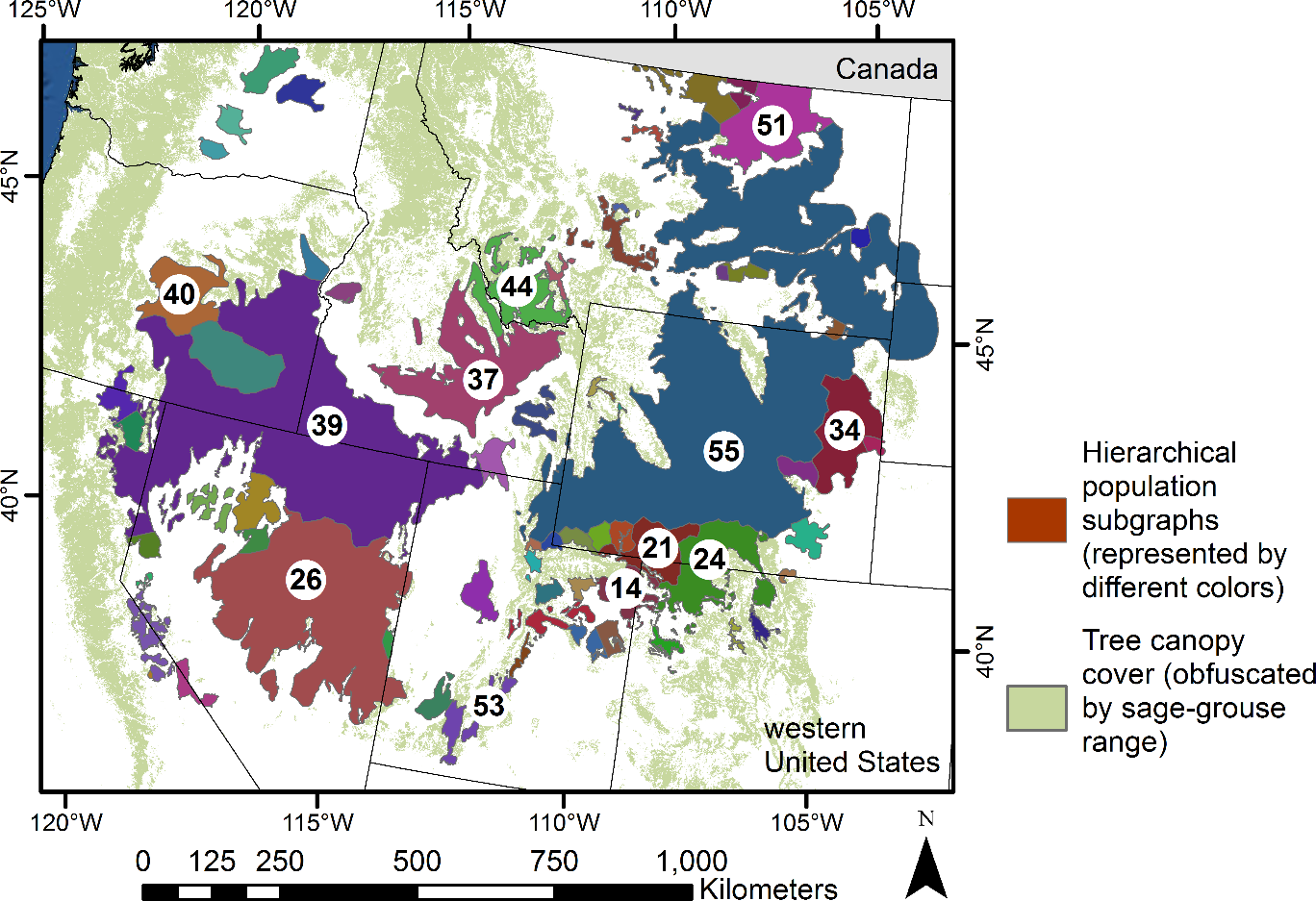
**

**Figure S3.** Thiessen polygons of greater sage-grouse (*Centrocercus urophasianus*) subpopulations (subgraphs) in the western United States, as defined by our hierarchal population structure (O'Donnell et al., 2022a). Subgraphs informed clustering of sage-grouse lek locations (breeding display grounds) into hierarchical cluster levels. Here, we show polygons of hierarchical population structure tier two (used for cluster levels three and four; moderate scales). The numeric labels of subgraph identifiers correspond to the column “Subgraph ID” in Table S6. Unlabeled polygons lacked enough leks for clustering (i.e., less than the maximum number of leks in Table S3 or detached leks in Table S4), resulting in no model in Table S6.


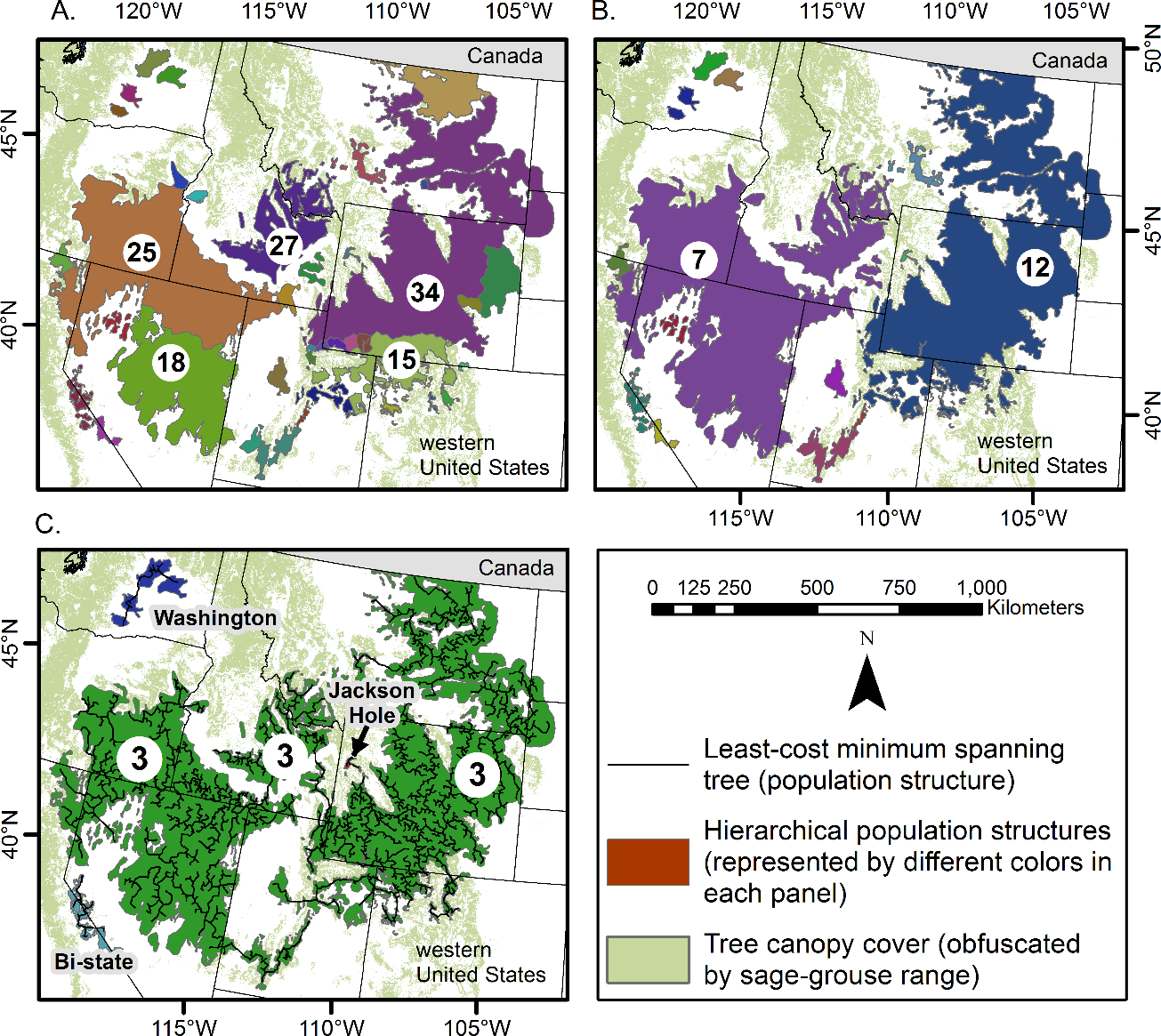


**Figure S4**. Thiessen polygons of greater sage-grouse (*Centrocercus urophasianus*) subpopulations (subgraphs) in the western United States, as defined by our hierarchal population structure (O'Donnell et al., 2022a). Subgraphs informed clustering of sage-grouse lek locations (breeding display grounds) into hierarchical cluster levels. Panel A represents subgraphs (population structure tier three) used in cluster levels five and six. Panel B reflects subgraphs (population structure tier four) used in cluster levels seven and eight. Panel C represents subgraphs (population structure tier five) used in cluster levels greater than eight. The numeric labels of subgraph identifiers correspond to the column “Subgraph ID” in Table S6. Unlabeled polygons lacked enough leks for clustering (i.e., less than the maximum number of leks in Table S3), resulting in no model in Table S6.

**Table S6**. Summary of top three combinations of habitat covariates, summary statistics, scale, and aspatial weights identified by Spatial “K”luster Analysis by Tree Edge Removal clustering algorithm for each model in a cluster level, while identifying greater sage-grouse (*Centrocercus urophasianus*) hierarchical population units in the western United States. Each model considered a habitat covariate, scale (radii surrounding a lek [breeding display ground] used to summarize underlying habitat), summary statistic of habitat covariate, and distance method of attribute space (non-spatial). Clustering occurred for each population subgraph captured in a hierarchical population structure that was represented by a least-cost path minimum spanning tree (LCP-MST). For example, Model identifier (ID) of 1 shows the three combinations of data, and a null model where a non-habitat covariate was used. The different summary statistics considered for the scales included coefficient of variation (CV), mean, and none (no statistic used [raw value]). The distance methods available to the clustering algorithm included Euclidean and Mahalanobis. We used the Akaike information criterion corrected for small sample sizes (AIC*_c_*) to rank each model (best to worst by subgraph [model ID] per population structure tier [LCP-MST]) and selected the top model. Each model can include one (univariate) or more (multivariable) covariates, scales, statistics, and spatial weight (“Distance method”). Subgraphs not clustered due to a lack of sufficient leks (defined by constraint-based rule specifying the number of leks to group at each cluster level) are denoted with ellipses.

| **Model ID** | **Cluster level** | **LCP-MST version** | **Subgraph ID** | **Selected covariate(s)** | **Selected scale** | **Statistic** | **Distance method** | **Sample size** | **AIC*_c_*** |
| --- | --- | --- | --- | --- | --- | --- | --- | --- | --- |
| 1 | 1 – 2 | LCP-MST-1 | 7 | Percent non-sagebrush shrub (pnss) | 6,400 m | CV | Euclidean | 37 | -111.85 |
|  | 1 – 2 | LCP-MST-1 | 7 | Mean temperature (°C) of wettest season (Bio8) | 1,500 m | mean | Euclidean | 37 | -99.10 |
|  | 1 – 2 | LCP-MST-1 | 7 | Percent cover bare ground | 1,000 m | mean | Euclidean | 37 | -96.51 |
|  | 1 – 2 | LCP-MST-1 | 7 | Null model | … | … | Euclidean | 37 | 886.05 |
| 2 | 1 – 2 | LCP-MST-1 | 23 | Annual precipitation totals (Bio12) | 6,400 m | mean | Euclidean | 42 | -66.92 |
|  | 1 – 2 | LCP-MST-1 | 23 | Percent non-big (other) sagebrush (pos) | 2,200 m | CV | Euclidean | 42 | -65.80 |
|  | 1 – 2 | LCP-MST-1 | 23 | Topographic position index | 250 m | none | Euclidean | 42 | -64.68 |
|  | 1 – 2 | LCP-MST-1 | 23 | Null model | … | … | Euclidean | 42 | 1,009.17 |
| 3 | 1 – 2 | LCP-MST-1 | 33 | Percent cover bare ground | 500 m | CV | Euclidean | 25 | -44.74 |
|  | 1 – 2 | LCP-MST-1 | 33 | Sagebrush height | 30 m | none | Euclidean | 25 | -42.72 |
|  | 1 – 2 | LCP-MST-1 | 33 | Hydrologically corrected digital elevation model (DEM); heat load index; shrub height | 3,000 m; 500 m; 1,000 m | CV; CV; mean | Euclidean | 25 | -39.60 |
|  | 1 – 2 | LCP-MST-1 | 33 | Null model | … | … | Euclidean | 25 | 600.66 |
| 4 | 1 – 2 | LCP-MST-1 | 35 | Precipitation seasonality (Bio15) | 30 m | none | Euclidean | 36 | -73.58 |
|  | 1 – 2 | LCP-MST-1 | 35 | Compound topographic index; percent cover herbaceous | 500 m; 4,700 m | CV; CV | Euclidean | 36 | -73.16 |
|  | 1 – 2 | LCP-MST-1 | 35 | Shrub height | 500 m | mean | Euclidean | 36 | -72.93 |
|  | 1 – 2 | LCP-MST-1 | 35 | Null model | … | … | Euclidean | 36 | 847.04 |
| 5 | 1 – 2 | LCP-MST-1 | 53 | Percent non-big (other) sagebrush (pos) | 500 m | mean | Euclidean | 41 | -111.34 |
|  | 1 – 2 | LCP-MST-1 | 53 | Percent non-big (other) sagebrush (pos) | 2,200 m | mean | Euclidean | 41 | -111.31 |
|  | 1 – 2 | LCP-MST-1 | 53 | Percent non-big (other) sagebrush (pos) | 1,500 m | mean | Euclidean | 41 | -105.67 |
|  | 1 – 2 | LCP-MST-1 | 53 | Null model | … | … | Euclidean | 41 | 992.96 |
| 6 | 1 – 2 | LCP-MST-1 | 61 | Percent non-big (other) sagebrush (pos) | 3,000 m | CV | Euclidean | 24 | -46.19 |
|  | 1 – 2 | LCP-MST-1 | 61 | Percent cover herbaceous; percent non-big (other) sagebrush (pos); percent cover all shrub | 1,000 m; 3,000 m; 2,200 m | mean; CV; CV | Euclidean | 24 | -44.38 |
|  | 1 – 2 | LCP-MST-1 | 61 | Percent cover herbaceous; percent non-sagebrush shrub (pnss) | 1,000 m; 1,500 m | mean; CV | Euclidean | 24 | -44.33 |
|  | 1 – 2 | LCP-MST-1 | 61 | Null model | … | … | Euclidean | 24 | 546.06 |
| 7 | 1 – 2 | LCP-MST-1 | 62 | Compound topographic index | 1,500 m | mean | Euclidean | 39 | -103.50 |
|  | 1 – 2 | LCP-MST-1 | 62 | Vector ruggedness index | 500 m | none | Euclidean | 39 | -98.07 |
|  | 1 – 2 | LCP-MST-1 | 62 | Compound topographic index | 2,200 m | mean | Euclidean | 39 | -89.55 |
|  | 1 – 2 | LCP-MST-1 | 62 | Null model | … | … | Euclidean | 39 | 968.64 |
| 8 | 1 – 2 | LCP-MST-1 | 63 | Percent perennial grass | 1,500 m | mean | Euclidean | 63 | -151.05 |
|  | 1 – 2 | LCP-MST-1 | 63 | Percent perennial grass | 1,000 m | mean | Euclidean | 63 | -140.46 |
|  | 1 – 2 | LCP-MST-1 | 63 | Percent cover bare ground | 2,200 m | CV | Euclidean | 63 | -136.10 |
|  | 1 – 2 | LCP-MST-1 | 63 | Null model |  |  |  |  | 1,501.06 |
| 9 | 1 – 2 | LCP-MST-1 | 74 | Percent non-big (other) sagebrush (pos) | 6,400 m | mean | Euclidean | 39 | -92.82 |
|  | 1 – 2 | LCP-MST-1 | 74 | Percent non-big (other) sagebrush (pos) | 4,700 m | mean | Euclidean | 39 | -73.67 |
|  | 1 – 2 | LCP-MST-1 | 74 | Sagebrush height | 6,400 m | mean | Euclidean | 39 | -61.11 |
|  | 1 – 2 | LCP-MST-1 | 74 | Null model | … | … | Euclidean | 39 | 956.33 |
| 10 | 1 – 2 | LCP-MST-1 | 79 | Compound topographic index; heat load index; percent cover bare ground | 500 m; 1,000 m; 6,400 m | mean; CV; CV | Euclidean | 77 | -138.13 |
|  | 1 – 2 | LCP-MST-1 | 79 | Topographic position index | 250 m | none | Euclidean | 77 | -134.02 |
|  | 1 – 2 | LCP-MST-1 | 79 | Topographic position index | 1,500 m | none | Euclidean | 77 | -132.31 |
|  | 1 – 2 | LCP-MST-1 | 79 | Null model | … | … | Euclidean | 77 | 1,862.92 |
| 11 | 1 – 2 | LCP-MST-1 | 82 | Shrub height | 1,500 m | mean | Euclidean | 254 | -412.36 |
|  | 1 – 2 | LCP-MST-1 | 82 | Hydrologically corrected digital elevation model (DEM) | 1,000 m | mean | Euclidean | 254 | -409.38 |
|  | 1 – 2 | LCP-MST-1 | 82 | Hydrologically corrected digital elevation model (DEM) | 1,500 m | mean | Euclidean | 254 | -402.31 |
|  | 1 – 2 | LCP-MST-1 | 82 | Null model | … | … | Euclidean | 254 | 6,154.72 |
| 12 | 1 – 2 | LCP-MST-1 | 83 | … | … | … | … | 16 | … |
| 13 | 1 – 2 | LCP-MST-1 | 84 | Percent non-big (other) sagebrush (pos) | 3,000 m | CV | Euclidean | 32 | -63.51 |
|  | 1 – 2 | LCP-MST-1 | 84 | Compound topographic index | 500 m | CV | Euclidean | 32 | -59.20 |
|  | 1 – 2 | LCP-MST-1 | 84 | Compound topographic index | 6,400 m | CV | Euclidean | 32 | -58.08 |
|  | 1 – 2 | LCP-MST-1 | 84 | Null model | … | … | Euclidean | 32 | 761.50 |
| 14 | 1 – 2 | LCP-MST-1 | 91 | Shrub height | 30 m | none | Euclidean | 25 | -83.88 |
|  | 1 – 2 | LCP-MST-1 | 91 | Percent cover all sagebrush | 500 m | CV | Euclidean | 25 | -73.99 |
|  | 1 – 2 | LCP-MST-1 | 91 | Percent perennial grass; sagebrush height; percent cover all shrub | 30 m; 500 m; 30 m | none; CV; none | Euclidean | 25 | -64.94 |
|  | 1 – 2 | LCP-MST-1 | 91 | Null model | … | … | Euclidean | 25 | 577.85 |
| 15 | 1 – 2 | LCP-MST-1 | 110 | … | … | … | … | 16 | … |
| 16 | 1 – 2 | LCP-MST-1 | 112 | … | … | … | … | 22 | … |
| 17 | 1 – 2 | LCP-MST-1 | 123 | … | … | … | … | 20 | … |
| 18 | 1 – 2 | LCP-MST-1 | 126 | Topographic position index | 30 m | none | Euclidean | 70 | -317.72 |
|  | 1 – 2 | LCP-MST-1 | 126 | Percent non-sagebrush shrub (pnss) | 500 m | CV | Euclidean | 70 | -137.34 |
|  | 1 – 2 | LCP-MST-1 | 126 | Topographic position index | 2,200 m | none | Euclidean | 70 | -124.24 |
|  | 1 – 2 | LCP-MST-1 | 126 | Null model | … | … | Euclidean | 70 | 1,696.32 |
| 19 | 1 – 2 | LCP-MST-1 | 129 | Precipitation seasonality (Bio15) | 6,400 m | mean | Euclidean | 928 | -2,036.07 |
|  | 1 – 2 | LCP-MST-1 | 129 | Precipitation seasonality (Bio15) | 4,700 m | mean | Euclidean | 928 | -1,875.93 |
|  | 1 – 2 | LCP-MST-1 | 129 | Precipitation seasonality (Bio15) | 2,200 m | mean | Euclidean | 928 | -1,660.09 |
|  | 1 – 2 | LCP-MST-1 | 129 | Null model | … | … | Euclidean | 928 | 22,486.65 |
| 20 | 1 – 2 | LCP-MST-1 | 135 | Shrub height | 6,400 m | mean | Euclidean | 162 | -267.91 |
|  | 1 – 2 | LCP-MST-1 | 135 | Percent cover big sagebrush | 30 m | none | Euclidean | 162 | -263.67 |
|  | 1 – 2 | LCP-MST-1 | 135 | Topographic position index | 500 m | none | Euclidean | 162 | -253.30 |
|  | 1 – 2 | LCP-MST-1 | 135 | Null model | … | … | Euclidean | 162 | 3,945.37 |
| 21 | 1 – 2 | LCP-MST-1 | 136 | Percent cover herbaceous | 500 m | CV | Euclidean | 23 | -50.70 |
|  | 1 – 2 | LCP-MST-1 | 136 | Percent cover herbaceous; percent perennial grass; percent cover all sagebrush | 500 m; pixel; 500 m | CV; none; CV | Euclidean | 23 | -44.15 |
|  | 1 – 2 | LCP-MST-1 | 136 | Vector ruggedness index; percent cover herbaceous | 4,700 m; 500 m | none; CV | Euclidean | 23 | -42.71 |
|  | 1 – 2 | LCP-MST-1 | 136 | Null model | … | … | Euclidean | 23 | 550.38 |
| 22 | 1 – 2 | LCP-MST-1 | 138 | Topographic position index | 30 m | none | Euclidean | 47 | -206.60 |
|  | 1 – 2 | LCP-MST-1 | 138 | Shrub height | 30 m | none | Euclidean | 47 | -114.66 |
|  | 1 – 2 | LCP-MST-1 | 138 | Percent non-big (other) sagebrush (pos) | 6,400 m | mean | Euclidean | 47 | -75.74 |
|  | 1 – 2 | LCP-MST-1 | 138 | Null model | … | … | Euclidean | 47 | 1,140.51 |
| 23 | 1 – 2 | LCP-MST-1 | 143 | … | … | … | … | 16 | … |
| 24 | 1 – 2 | LCP-MST-1 | 145 | Percent cover bare ground | 500 m | mean | Euclidean | 139 | -375.93 |
|  | 1 – 2 | LCP-MST-1 | 145 | Percent cover bare ground | 2,200 m | mean | Euclidean | 139 | -366.53 |
|  | 1 – 2 | LCP-MST-1 | 145 | Percent cover bare ground | 1,500 m | mean | Euclidean | 139 | -363.86 |
|  | 1 – 2 | LCP-MST-1 | 145 | Null model | … | … | Euclidean | 139 | 3,324.44 |
| 25 | 1 – 2 | LCP-MST-1 | 146 | Precipitation seasonality (Bio15) | 6,400 m | mean | Euclidean | 385 | -1,050.46 |
|  | 1 – 2 | LCP-MST-1 | 146 | Precipitation seasonality (Bio15) | 3,000 m | mean | Euclidean | 385 | -1,028.67 |
|  | 1 – 2 | LCP-MST-1 | 146 | Precipitation seasonality (Bio15) | 2,200 m | mean | Euclidean | 385 | -1,018.99 |
|  | 1 – 2 | LCP-MST-1 | 146 | Null model | … | … | Euclidean | 385 | 9,254.44 |
| 26 | 1 – 2 | LCP-MST-1 | 151 | Hydrologically corrected digital elevation model (DEM) | 30 m | none | Euclidean | 141 | -362.63 |
|  | 1 – 2 | LCP-MST-1 | 151 | Hydrologically corrected digital elevation model (DEM) | 1,500 m | mean | Euclidean | 141 | -355.03 |
|  | 1 – 2 | LCP-MST-1 | 151 | Hydrologically corrected digital elevation model (DEM) | 500 m | mean | Euclidean | 141 | -353.03 |
|  | 1 – 2 | LCP-MST-1 | 151 | Null model | … | … | Euclidean | 141 | 3,579.67 |
| 27 | 1 – 2 | LCP-MST-1 | 152 | Percent non-sagebrush shrub (pnss) | 6,400 m | CV | Euclidean | 30 | -91.69 |
|  | 1 – 2 | LCP-MST-1 | 152 | Percent non-sagebrush shrub (pnss) | 4,700 m | CV | Euclidean | 30 | -77.53 |
|  | 1 – 2 | LCP-MST-1 | 152 | Percent non-sagebrush shrub (pnss); sagebrush height | 6,400 m | CV; mean | Euclidean | 30 | -59.61 |
|  | 1 – 2 | LCP-MST-1 | 152 | Null model | … | … | Euclidean | 30 | 733.65 |
| 28 | 1 – 2 | LCP-MST-1 | 165 | Percent perennial grass | 1,000 m | mean | Euclidean | 22 | -50.87 |
|  | 1 – 2 | LCP-MST-1 | 165 | Percent perennial grass | 4,700 m | CV | Euclidean | 22 | -45.64 |
|  | 1 – 2 | LCP-MST-1 | 165 | Percent perennial grass | 1,500 m | mean | Euclidean | 22 | -45.59 |
|  | 1 – 2 | LCP-MST-1 | 165 | Null model |  |  |  | 22 | 526.81 |
| 29 | 1 – 2 | LCP-MST-1 | 166 | Precipitation seasonality (Bio15) | 6,400 m | mean | Euclidean | 1,128 | -2,652.82 |
|  | 1 – 2 | LCP-MST-1 | 166 | Precipitation seasonality (Bio15) | 1,500 m | mean | Euclidean | 1,128 | -2,598.20 |
|  | 1 – 2 | LCP-MST-1 | 166 | Precipitation seasonality (Bio15) | 2,200 m | mean | Euclidean | 1,128 | -2,590.92 |
|  | 1 – 2 | LCP-MST-1 | 166 | Null model | … | … | Euclidean | 1,128 | 27,321.46 |
| 30 | 1 – 2 | LCP-MST-1 | 170 | … | … | … | … | 18 | … |
| 31 | 1 – 2 | LCP-MST-1 | 173 | Vector ruggedness index | 1,500 m | none | Euclidean | 26 | -67.08 |
|  | 1 – 2 | LCP-MST-1 | 173 | Vector ruggedness index | 1,000 m | none | Euclidean | 26 | -50.09 |
|  | 1 – 2 | LCP-MST-1 | 173 | Percent perennial grass | 3,000 m | mean | Euclidean | 26 | -44.40 |
|  | 1 – 2 | LCP-MST-1 | 173 | Null model | … | … | Euclidean | 26 | 647.37 |
| 32 | 1 – 2 | LCP-MST-1 | 177 | Percent cover big sagebrush | 30 m | none | Euclidean | 35 | -67.51 |
|  | 1 – 2 | LCP-MST-1 | 177 | Compound topographic index | 4,700 m | CV | Euclidean | 35 | -63.88 |
|  | 1 – 2 | LCP-MST-1 | 177 | Compound topographic index | 1,500 m | CV | Euclidean | 35 | -60.65 |
|  | 1 – 2 | LCP-MST-1 | 177 | Null model | … | … | Euclidean | 35 | 846.81 |
| 33 | 1 – 2 | LCP-MST-1 | 178 | Percent perennial grass | 6,400 m | mean | Euclidean | 44 | -115.73 |
|  | 1 – 2 | LCP-MST-1 | 178 | Percent cover all sagebrush | 6,400 m | CV | Euclidean | 44 | -115.60 |
|  | 1 – 2 | LCP-MST-1 | 178 | Percent cover herbaceous | 4,700 m | CV | Euclidean | 44 | -108.43 |
|  | 1 – 2 | LCP-MST-1 | 178 | Null model | … | … | Euclidean | 44 | 1,047.52 |
| 34 | 1 – 2 | LCP-MST-1 | 180 | Percent cover big sagebrush | 1,500 m | CV | Euclidean | 25 | -56.63 |
|  | 1 – 2 | LCP-MST-1 | 180 | Percent cover big sagebrush | 500 m | CV | Euclidean | 25 | -46.77 |
|  | 1 – 2 | LCP-MST-1 | 180 | Shrub height | 1,000 m | CV | Euclidean | 25 | -43.71 |
|  | 1 – 2 | LCP-MST-1 | 180 | Null model | … | … | Euclidean | 25 | 600.34 |
| 35 | 1 – 2 | LCP-MST-1 | 181 | Heat load index | 1,500 m | mean | Euclidean | 23 | -99.12 |
|  | 1 – 2 | LCP-MST-1 | 181 | Heat load index | 1,000 m | mean | Euclidean | 23 | -96.97 |
|  | 1 – 2 | LCP-MST-1 | 181 | Heat load index | 1,500 m | CV | Euclidean | 23 | -92.31 |
|  | 1 – 2 | LCP-MST-1 | 181 | Null model | … | … | Euclidean | 23 | 522.22 |
| 36 | 1 – 2 | LCP-MST-1 | 183 | Mean temperature (°C) of wettest season (Bio8) | 2,200 m | mean | Euclidean | 178 | -494.61 |
|  | 1 – 2 | LCP-MST-1 | 183 | Mean temperature (°C) of wettest season (Bio8) | 3,000 m | mean | Euclidean | 178 | -490.23 |
|  | 1 – 2 | LCP-MST-1 | 183 | Mean temperature (°C) of wettest season (Bio8) | 1,500 m | mean | Euclidean | 178 | -469.04 |
|  | 1 – 2 | LCP-MST-1 | 183 | Null model | … | … | Euclidean | 178 | 4,347.85 |
| 37 | 1 – 2 | LCP-MST-1 | 185 | Percent cover big sagebrush | 30 m | none | Euclidean | 28 | -60.17 |
|  | 1 – 2 | LCP-MST-1 | 185 | Topographic position index | 1,500 m | none | Euclidean | 28 | -56.67 |
|  | 1 – 2 | LCP-MST-1 | 185 | Heat load index; percent cover big sagebrush; percent cover herbaceous | 30 m; 30 m; 6,400 m | none; none; mean | Euclidean | 28 | -55.14 |
|  | 1 – 2 | LCP-MST-1 | 185 | Null model | … | … | Euclidean | 28 | 670.41 |
| 38 | 1 – 2 | LCP-MST-1 | 190 | Vector ruggedness index | 6,400 m | none | Euclidean | 55 | -117.05 |
|  | 1 – 2 | LCP-MST-1 | 190 | Percent non-sagebrush shrub (pnss) | 3,000 m | CV | Euclidean | 55 | -111.89 |
|  | 1 – 2 | LCP-MST-1 | 190 | Percent cover big sagebrush | 2,200 m | mean | Euclidean | 55 | -107.06 |
|  | 1 – 2 | LCP-MST-1 | 190 | Null model | … | … | Euclidean | 55 | 1,347.55 |
| 39 | 1 – 2 | LCP-MST-1 | 191 | Topographic position index | 30 m | none | Euclidean | 23 | -56.59 |
|  | 1 – 2 | LCP-MST-1 | 191 | Topographic position index | 4,700 m | none | Euclidean | 23 | -56.000 |
|  | 1 – 2 | LCP-MST-1 | 191 | Topographic position index; percent perennial grass; percent non-big (other) sagebrush (pos) | 30 m; 1,000 m; pixel | none; CV; none | Euclidean | 23 | -46.26 |
|  | 1 – 2 | LCP-MST-1 | 191 | Null model | … | … | Euclidean | 23 | 572.77 |
| 40 | 1 – 2 | LCP-MST-1 | 194 | Percent non-sagebrush shrub (pnss) | 500 m | mean | Euclidean | 94 | -187.98 |
|  | 1 – 2 | LCP-MST-1 | 194 | Hydrologically corrected digital elevation model (DEM) | 2,200 m | mean | Euclidean | 94 | -185.33 |
|  | 1 – 2 | LCP-MST-1 | 194 | Hydrologically corrected digital elevation model (DEM) | 3,000 m | mean | Euclidean | 94 | -185.27 |
|  | 1 – 2 | LCP-MST-1 | 194 | Null model | … | … | Euclidean | 94 | 2,303.62 |
| 41 | 1 – 2 | LCP-MST-1 | 195 | Mean temperature (°C) of wettest season (Bio8) | 30 m | none | Euclidean | 192 | -669.76 |
|  | 1 – 2 | LCP-MST-1 | 195 | Mean temperature (°C) of wettest season (Bio8) | 1,500 m | mean | Euclidean | 192 | -644.16 |
|  | 1 – 2 | LCP-MST-1 | 195 | Mean temperature (°C) of wettest season (Bio8) | 2,200 m | mean | Euclidean | 192 | -599.38 |
|  | 1 – 2 | LCP-MST-1 | 195 | Null model | … | … | Euclidean | 192 | 4,690.24 |
| 42 | 1 – 2 | LCP-MST-1 | 197 | … | … | … | … | 19 | … |
| 43 | 1 – 2 | LCP-MST-1 | 201 | Percent perennial grass | 1,000 m | CV | Euclidean | 33 | -70.81 |
|  | 1 – 2 | LCP-MST-1 | 201 | Shrub height | 30 m | none | Euclidean | 33 | -69.33 |
|  | 1 – 2 | LCP-MST-1 | 201 | Percent cover herbaceous | 1,000 m | CV | Euclidean | 33 | -68.05 |
|  | 1 – 2 | LCP-MST-1 | 201 | Null model |  |  |  | 33 | 784.68 |
| 44 | 1 – 2 | LCP-MST-1 | 202 | Percent cover herbaceous | 1,500 m | mean | Euclidean | 116 | -190.81 |
|  | 1 – 2 | LCP-MST-1 | 202 | Percent perennial grass | 1,000 m | mean | Euclidean | 116 | -178.20 |
|  | 1 – 2 | LCP-MST-1 | 202 | Percent cover bare ground | 1,500 m | mean | Euclidean | 116 | -175.51 |
|  | 1 – 2 | LCP-MST-1 | 202 | Null model | … | … | Euclidean | 116 | 2,849.58 |
| 45 | 1 – 2 | LCP-MST-1 | 213 | Percent cover herbaceous | 1,000 m | CV | Euclidean | 30 | -62.06 |
|  | 1 – 2 | LCP-MST-1 | 213 | Percent non-sagebrush shrub (pnss) | 30 m | none | Euclidean | 30 | -54.97 |
|  | 1 – 2 | LCP-MST-1 | 213 | Heat load index | 30 m | none | Euclidean | 30 | -48.00 |
|  | 1 – 2 | LCP-MST-1 | 213 | Null model | … | … | Euclidean | 30 | 737.55 |
| 46 | 3 – 4 | LCP-MST-2 | 5 | … | … | … | … | 44 | … |
| 47 | 3 – 4 | LCP-MST-2 | 7 | … | … | … | … | 28 | … |
| 48 | 3 – 4 | LCP-MST-2 | 8 | … | … | … | … | 19 | … |
| 49 | 3 – 4 | LCP-MST-2 | 9 | … | … | … | … | 59 | … |
| 50 | 3 – 4 | LCP-MST-2 | 14 | Percent cover all sagebrush | 30 m | none | Euclidean | 70 | 53.76 |
|  | 3 – 4 | LCP-MST-2 | 14 | Percent non-sagebrush shrub (pnss) | 6,400 m | CV | Euclidean | 70 | 57.21 |
|  | 3 – 4 | LCP-MST-2 | 14 | Sagebrush height | 6,400 m | CV | Euclidean | 70 | 60.63 |
|  | 3 – 4 | LCP-MST-2 | 14 | Null model | … | … | Euclidean | 70 | 1,867.23 |
| 51 | 3 – 4 | LCP-MST-2 | 21 | Percent cover bare ground | 30 m | none | Euclidean | 81 | -145.33 |
|  | 3 – 4 | LCP-MST-2 | 21 | Percent cover bare ground | 30 m; 30 m | none; none | Euclidean | 81 | -125.27 |
|  | 3 – 4 | LCP-MST-2 | 21 | Percent cover herbaceous | 30 m | none | Euclidean | 81 | -107.71 |
|  | 3 – 4 | LCP-MST-2 | 21 | Null model | … | … | Euclidean |  | 2,126.05 |
| 52 | 3 – 4 | LCP-MST-2 | 24 | Percent non-sagebrush shrub (pnss); shrub height | 500 m; pixel | CV; none | Mahalanobis | 311 | -274.68 |
|  | 3 – 4 | LCP-MST-2 | 24 | Percent non-sagebrush shrub (pnss) | 500 m | CV | Euclidean | 311 | 35.46 |
|  | 3 – 4 | LCP-MST-2 | 24 | Hydrologically corrected digital elevation model (DEM); percent non-sagebrush shrub (pnss) | 2,200 m; 500 m | mean; CV | Mahalanobis | 311 | 108.38 |
|  | 3 – 4 | LCP-MST-2 | 24 | Null model | … | … | Euclidean | 311 | 8,287.67 |
| 53 | 3 – 4 | LCP-MST-2 | 25 | … | … | … | … | 16 | … |
| 54 | 3 – 4 | LCP-MST-2 | 26 | Percent cover all shrub | 6,400 m | CV | Euclidean | 325 | -73.73 |
|  | 3 – 4 | LCP-MST-2 | 26 | Percent cover herbaceous | 2,200 m | mean | Euclidean | 325 | -63.30 |
|  | 3 – 4 | LCP-MST-2 | 26 | Percent non-sagebrush shrub (pnss) | 4,700 m | CV | Euclidean | 325 | -51.90 |
|  | 3 – 4 | LCP-MST-2 | 26 | Null model | … | … | Euclidean | 325 | 8,110.59 |
| 55 | 3 – 4 | LCP-MST-2 | 28 | … | … | … | … | 21 | … |
| 56 | 3 – 4 | LCP-MST-2 | 31 | … | … | … | … | 24 | … |
| 57 | 3 – 4 | LCP-MST-2 | 34 | Percent non-big (other) sagebrush (pos) | 2,200 m | CV | Euclidean | 97 | -29.26 |
|  | 3 – 4 | LCP-MST-2 | 34 | Sagebrush height | 6,400 m | mean | Euclidean | 97 | -20.94 |
|  | 3 – 4 | LCP-MST-2 | 34 | Compound topographic index | 30 m | none | Euclidean | 97 | -16.58 |
|  | 3 – 4 | LCP-MST-2 | 34 | Null model | … | … | Euclidean | 97 | 2,487.22 |
| 58 | 3 – 4 | LCP-MST-2 | 36 | … | … | … | … | 41 | … |
| 59 | 3 – 4 | LCP-MST-2 | 37 | Sagebrush height; mean temperature (°C) of wettest season (Bio8) | 6,400 m; 6,400 m | mean; mean | Mahalanobis | 597 | -422.66 |
|  | 3 – 4 | LCP-MST-2 | 37 | Sagebrush height | 6,400 m | mean | Euclidean | 597 | 41.18 |
|  | 3 – 4 | LCP-MST-2 | 37 | Percent cover bare ground; mean temperature (°C) of wettest season (Bio8) | 4,700 m; 6,4000 m | mean; mean; CV | Mahalanobis | 597 | 108.12 |
|  | 3 – 4 | LCP-MST-2 | 37 | Null model | … | … | Euclidean | 597 | 15,830.26 |
| 60 | 3 – 4 | LCP-MST-2 | 39 | Percent cover herbaceous | 4,700 m | CV | Euclidean | 1,383 | 1,503.47 |
|  | 3 – 4 | LCP-MST-2 | 39 | Percent cover herbaceous | 500 m | CV | Euclidean | 1,383 | 1,589.97 |
|  | 3 – 4 | LCP-MST-2 | 39 | Percent cover herbaceous; mean temperature (°C) of wettest season (Bio8) | 4,700 m; 4,700 m | CV; mean | Mahalanobis | 1,383 | 1,596.06 |
|  | 3 – 4 | LCP-MST-2 | 39 | Null model | … | … | Euclidean | 1,383 | 36,765.94 |
| 61 | 3 – 4 | LCP-MST-2 | 40 | Percent cover all shrub | 3,000 m | CV | Euclidean | 78 | -108.86 |
|  | 3 – 4 | LCP-MST-2 | 40 | Percent cover all sagebrush | 3,000 m | CV | Euclidean | 78 | -105.19 |
|  | 3 – 4 | LCP-MST-2 | 40 | Percent cover all shrub | 4,700 m | CV | Euclidean | 78 | -98.66 |
|  | 3 – 4 | LCP-MST-2 | 40 | Null model | … | … | Euclidean | 78 | 1,985.92 |
| 62 | 3 – 4 | LCP-MST-2 | 42 | … | … | … | … | 28 | … |
| 63 | 3 – 4 | LCP-MST-2 | 44 | Sagebrush height | 4,700 m | mean | Euclidean | 103 | -132.29 |
|  | 3 – 4 | LCP-MST-2 | 44 | Sagebrush height | 6,400 m | mean | Euclidean | 103 | -77.95 |
|  | 3 – 4 | LCP-MST-2 | 44 | Mean temperature (°C) of wettest season (Bio8) | 3,000 m | mean | Euclidean | 103 | 2.16 |
|  | 3 – 4 | LCP-MST-2 | 44 | Null model | … | … | Euclidean | 103 | 2,755.03 |
| 64 | 3 – 4 | LCP-MST-2 | 51 | Compound topographic index | 2,200 m | mean | Euclidean | 137 | 31.68 |
|  | 3 – 4 | LCP-MST-2 | 51 | Compound topographic index | 3,000 m | mean | Euclidean | 137 | 151.97 |
|  | 3 – 4 | LCP-MST-2 | 51 | Heat load index | 1,000 m | CV | Euclidean | 137 | 167.88 |
|  | 3 – 4 | LCP-MST-2 | 51 | Null model | … | … | Euclidean | 137 | 3,828.48 |
| 65 | 3 – 4 | LCP-MST-2 | 53 | Hydrologically corrected digital elevation model (DEM) | 6,400 m | mean | Euclidean | 72 | -160.88 |
|  | 3 – 4 | LCP-MST-2 | 53 | Mean temperature (°C) of warmest quarter (Bio10) | 6,400 m | mean | Euclidean | 72 | -130.90 |
|  | 3 – 4 | LCP-MST-2 | 53 | Hydrologically corrected digital elevation model (DEM) | 3,000 m | mean | Euclidean | 72 | -125.92 |
|  | 3 – 4 | LCP-MST-2 | 53 | Null model | … | … | Euclidean | 72 | 1,688.79 |
| 66 | 3 – 4 | LCP-MST-2 | 55 | Hydrologically corrected digital elevation model (DEM) | 4,700 m | mean | Euclidean | 2,028 | -815.49 |
|  | 3 – 4 | LCP-MST-2 | 55 | Hydrologically corrected digital elevation model (DEM) | 30 m | none | Euclidean | 2,028 | -714.64 |
|  | 3 – 4 | LCP-MST-2 | 55 | Hydrologically corrected digital elevation model (DEM) | 6,400 m | mean | Euclidean | 2,028 | -592.34 |
|  | 3 – 4 | LCP-MST-2 | 55 | Null model | … | … | Euclidean | 2,028 | 53,948.73 |
| 67 | 3 – 4 | LCP-MST-2 | 56 | … | … | … | … | 30 | … |
| 68 | 5 – 6 | LCP-MST-3 | 4 | … | … | … | … | 44 | … |
| 69 | 5 – 6 | LCP-MST-3 | 6 | … | … | … | … | 28 | … |
| 70 | 5 – 6 | LCP-MST-3 | 7 | … | … | … | … | 25 | … |
| 71 | 5 – 6 | LCP-MST-3 | 9 | … | … | … | … | 63 | … |
| 72 | 5 – 6 | LCP-MST-3 | 15 | Percent cover all shrub | 4,700 m | CV | Euclidean | 478 | 1,415.18 |
|  | 5 – 6 | LCP-MST-3 | 15 | Shrub height | 30 m | none | Euclidean | 478 | 1,479.59 |
|  | 5 – 6 | LCP-MST-3 | 15 | Heat load index | 1,500 m | mean | Euclidean | 478 | 1,512.50 |
|  | 5 – 6 | LCP-MST-3 | 15 | Null model | … | … | Euclidean | 478 | 12,326.10 |
| 73 | 5 – 6 | LCP-MST-3 | 16 | … | … | … | … | 16 | … |
| 74 | 5 – 6 | LCP-MST-3 | 18 | Percent non-sagebrush shrub (pnss) | 500 m | CV | Euclidean | 349 | 124.87 |
|  | 5 – 6 | LCP-MST-3 | 18 | Compound topographic index; percent non-sagebrush shrub (pnss) | 6,400 m; 500 m | mean; CV | Mahalanobis | 349 | 172.03 |
|  | 5 – 6 | LCP-MST-3 | 18 | Compound topographic index; shrub height | 6,400 m; 6,400 m | mean; CV | Mahalanobis | 349 | 246.34 |
|  | 5 – 6 | LCP-MST-3 | 18 | Null model | … | … | Euclidean | 349 | 9,176.73 |
| 75 | 5 – 6 | LCP-MST-3 | 20 | … | … | … | … | 24 | … |
| 76 | 5 – 6 | LCP-MST-3 | 22 | … | … | … | … | 99 | … |
| 77 | 5 – 6 | LCP-MST-3 | 25 | Percent non-big (other) sagebrush (pos); sagebrush height | 30 m; 6,400 m | none; mean | Mahalanobis | 1,505 | 1,885.81 |
|  | 5 – 6 | LCP-MST-3 | 25 | Compound topographic index; sagebrush height | 1,000 m; 6,400 m | mean; mean | Mahalanobis | 1,505 | 2,481.580 |
|  | 5 – 6 | LCP-MST-3 | 25 | Sagebrush height | 6,400 m | mean | Euclidean | 1,505 | 2,875.74 |
|  | 5 – 6 | LCP-MST-3 | 25 | Null model | … | … | Euclidean | 1,505 | 42,508.86 |
| 78 | 5 – 6 | LCP-MST-3 | 26 | … | … | … | … | 28 | … |
| 79 | 5 – 6 | LCP-MST-3 | 27 | Percent cover all sagebrush | 500 m | CV | Euclidean | 701 | 2,010.67 |
|  | 5 – 6 | LCP-MST-3 | 27 | Mean temperature (°C) of wettest season (Bio8) | 30 m | none | Euclidean | 701 | 2,030.85 |
|  | 5 – 6 | LCP-MST-3 | 27 | Mean temperature (°C) of wettest season (Bio8) | 1,500 m | mean | Euclidean | 701 | 2,031.80 |
|  | 5 – 6 | LCP-MST-3 | 27 | Null model | … | … | Euclidean | 701 | 19,842.51 |
| 80 | 5 – 6 | LCP-MST-3 | 31 | … | … | … | … | 144 | … |
| 81 | 5 – 6 | LCP-MST-3 | 32 | … | … | … | … | 72 | … |
| 82 | 5 – 6 | LCP-MST-3 | 34 | Hydrologically corrected digital elevation model (DEM) | 1,500 m | mean | Euclidean | 2,507 | 3,409.90 |
|  | 5 – 6 | LCP-MST-3 | 34 | Hydrologically corrected digital elevation model (DEM) | 1,000 m | mean | Euclidean | 2,507 | 3,477.32 |
|  | 5 – 6 | LCP-MST-3 | 34 | Hydrologically corrected digital elevation model (DEM) | 2,200 m | mean | Euclidean | 2,507 | 3,527.57 |
|  | 5 – 6 | LCP-MST-3 | 34 | Null model | … | … | Euclidean | 2,507 | 56,029.57 |
| 83 | 5 – 6 | LCP-MST-3 | 35 | … | … | … | … | 30 | … |
| 84 | 7 – 8 | LCP-MST-4 | 4 | … | … | … | … | 64 | … |
| 85 | 7 – 8 | LCP-MST-4 | 7 | Percent cover herbaceous; annual mean temperature (°C; Bio1) | 30 m; 30 m | none; none | Mahalanobis | 2,635 | 7,204.32 |
|  | 7 – 8 | LCP-MST-4 | 7 | Percent perennial grass | 30 m | none | Euclidean | 2,635 | 7,756.38 |
|  | 7 – 8 | LCP-MST-4 | 7 | Percent cover herbaceous | 30 m | none | Euclidean | 2,635 | 7,873.44 |
|  | 7 – 8 | LCP-MST-4 | 7 | Null model | … | … | Euclidean | 2,635 | 71,239.23 |
| 86 | 7 – 8 | LCP-MST-4 | 9 | … | … | … | … | 16 | … |
| 87 | 7 – 8 | LCP-MST-4 | 10 | … | … | … | … | 86 | … |
| 88 | 7 – 8 | LCP-MST-4 | 12 | Percent cover big sagebrush; sagebrush height | 30 m; 30 m | none; none | Mahalanobis | 2,955 | 5,851.77 |
|  | 7 – 8 | LCP-MST-4 | 12 | Percent cover big sagebrush | 30 m | none | Euclidean | 2,955 | 6,978.17 |
|  | 7 – 8 | LCP-MST-4 | 12 | Percent cover big sagebrush; precipitation seasonality (Bio15) | 30 m; 6,400 m | none; mean | Mahalanobis | 2,955 | 7,219.77 |
|  | 7 – 8 | LCP-MST-4 | 12 | Null model | … | … | Euclidean | 2,955 | 84,369.87 |
| 89 | 7 – 8 | LCP-MST-4 | 13 | … | … | … | … | 30 | … |
| 90 | 9 – 10 | LCP-MST-5 | 1 | … | … | … | … | 66 | … |
| 91 | 9 – 10 | LCP-MST-5 | 3 | Mean temperature (°C) of wettest season (Bio8) | 2,200 m | mean | Euclidean | 5,707 | 13,525.11 |
|  | 9 – 10 | LCP-MST-5 | 3 | Mean temperature (°C) of wettest season (Bio8) | 1,500 m | mean | Euclidean | 5,707 | 13,972.47 |
|  | 9 – 10 | LCP-MST-5 | 3 | Mean temperature (°C) of wettest season (Bio8) | 3,000 m | mean | Euclidean | 5,707 | 14,586.21 |
|  | 9 – 10 | LCP-MST-5 | 3 | Null model | … | … | Euclidean | 5,707 | 140,172.42 |
| 92 | 9 – 10 | LCP-MST-5 | 4 | … | … | … | … | 47 | … |
| 93 | 11 – 12 | LCP-MST-5 | 1 | … | … | … | … | 66 | … |
| 94 | 11 – 12 | LCP-MST-5 | 3 | Percent non-sagebrush shrub (pnss) | 30 m | none | Euclidean | 5,707 | -243.70 |
|  | 11 – 12 | LCP-MST-5 | 3 | Topographic position index | 1,500 m | none | Euclidean | 5,707 | 15,717.91 |
|  | 11 – 12 | LCP-MST-5 | 3 | Topographic position index | 2,200 m | none | Euclidean | 5,707 | 15,949.33 |
|  | 11 – 12 | LCP-MST-5 | 3 | Null model | … | … | Euclidean | 5,707 | 139,765.33 |
| 95 | 11 – 12 | LCP-MST-5 | 4 | … | … | … | … | 47 | … |
| 96 | 13 | LCP-MST-5 | 1 | … | … | … | … | 66 | … |
| 97 | 13 | LCP-MST-5 | 3 | Heat load index | 6,400 m | CV | Euclidean | 5,707 | 40,390.37 |
|  | 13 | LCP-MST-5 | 3 | Heat load index | 4,700 m | CV | Euclidean | 5,707 | 40,807.58 |
|  | 13 | LCP-MST-5 | 3 | Hydrologically corrected digital elevation model (DEM) | 6,400 m | CV | Euclidean | 5,707 | 40,874.49 |
|  | 13 | LCP-MST-5 | 3 | Null model | … | … | Euclidean | 5,707 | 139,988.91 |
| 98 | 13 | LCP-MST-5 | 4 | … | … | … | … | 47 | … |


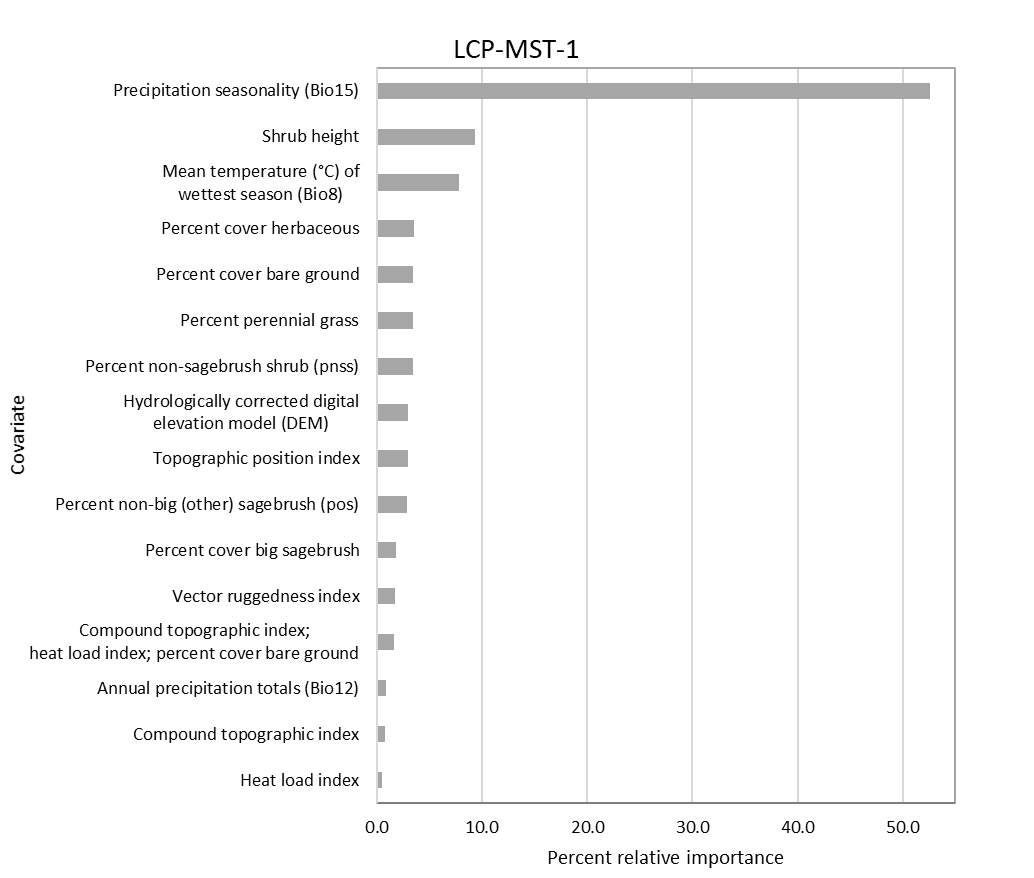


**Figure S5**. Relative importance of covariates selected in the top model of the Spatial “K”luster Analysis by Tree Edge Removal clustering algorithm for population structure tier one while identifying greater sage-grouse (*Centrocercus urophasianus*) hierarchical population units in the western United States. Least-cost path minimum spanning tree, tier 1(LCP-MST-1).

| 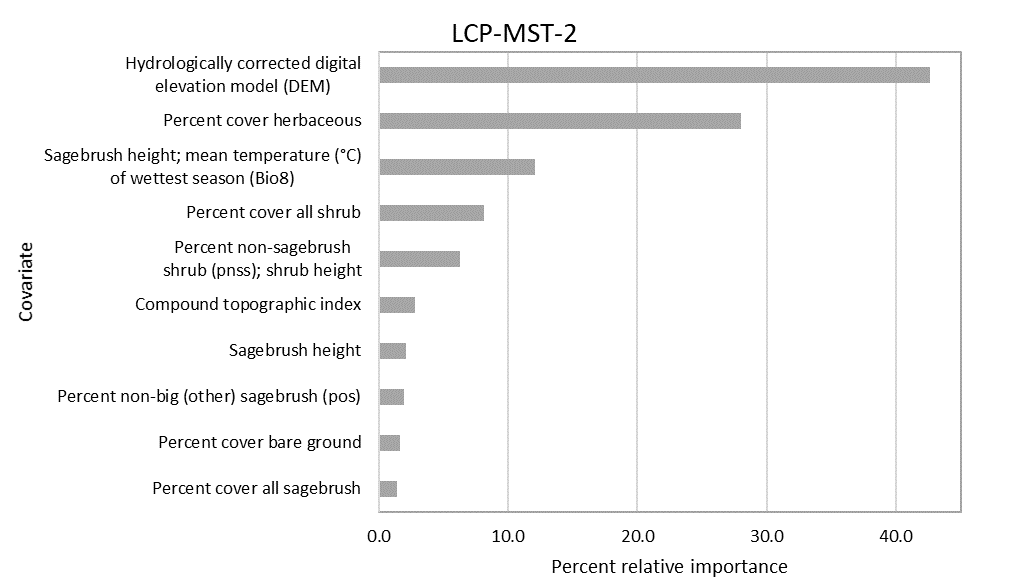 |
| --- |
| 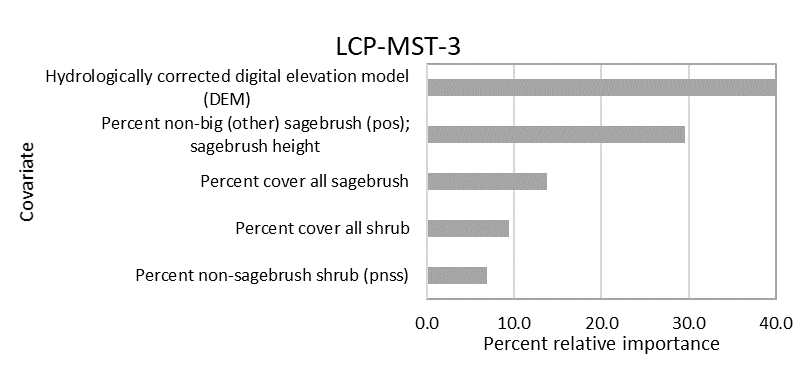 |
| 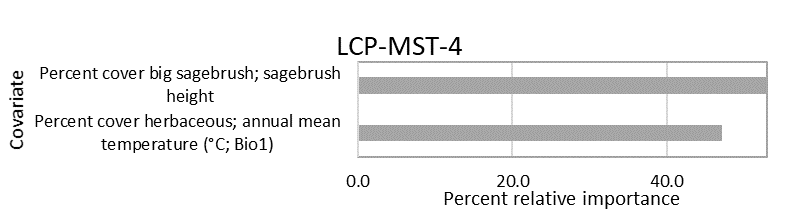 |

**Figure S6**. Relative importance of covariates selected in the top model of the Spatial “K”luster Analysis by Tree Edge Removal clustering algorithm for population structure tiers two to four while identifying greater sage-grouse (*Centrocercus urophasianus*) hierarchical population units in the western United States. For LCP-MST-5 (least-cost path minimum spanning tree tier 5; not shown), cluster levels 9 – 10 identified sagebrush height and mean temperature (°C) of wettest season (Bio8). Similarly, cluster levels 11 – 12 identified percent non-sagebrush shrub (pnss), and cluster-level 13 identified heat load index. The LCP-MST-5 was not displayed because it had no competing models.

| 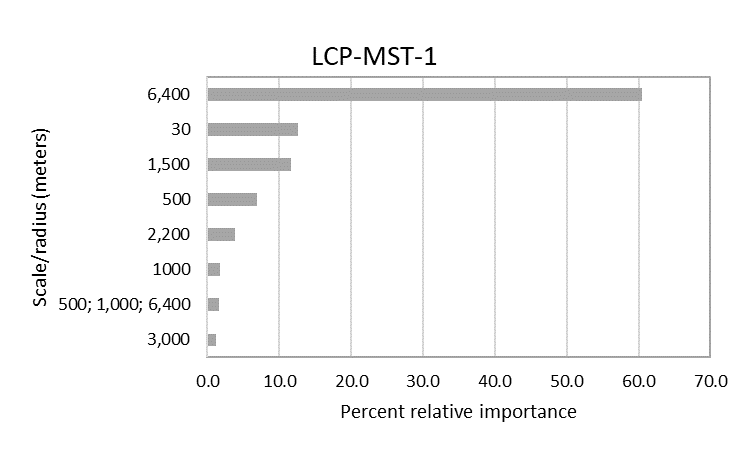 |
| --- |
| 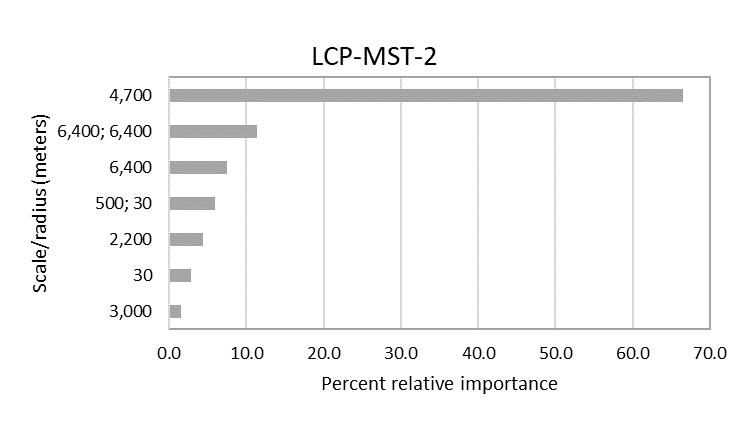 |
| 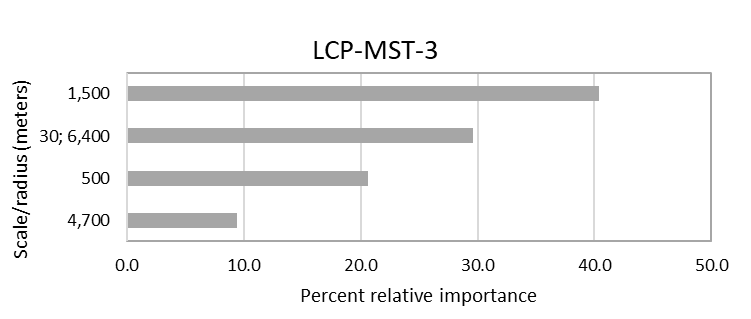 |

**Figure S7**. Relative importance of scales selected in the top model of the Spatial “K”luster Analysis by Tree Edge Removal clustering algorithm for each population structure tier while identifying greater sage-grouse (*Centrocercus urophasianus*) hierarchical population units in the western United States. The LCP-MST-4 (least-cost path minimum spanning tree tier 4) identified a scale of 30 meters with no competing models. For LCP-MST-5, cluster levels 9 – 10 identified a scale of 2,200 meters, cluster levels 11 – 12 identified a scale of 30 meters, and cluster-level 13 identified a scale of 6,400 meters. The LCP-MST-4 and -5 were not displayed because they had no competing models.

| **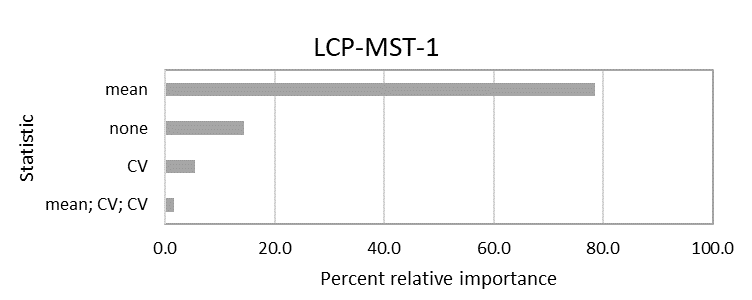** |
| --- |
| **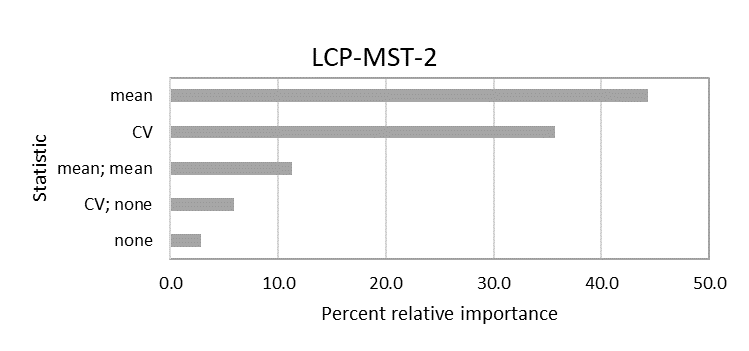** |
| **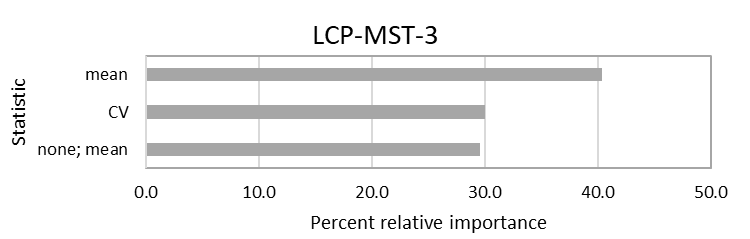** |

**Figure S8**. Relative importance of summary statistics selected in top model of the Spatial “K”luster Analysis by Tree Edge Removal clustering algorithm for each population structure tier while identifying greater sage-grouse (*Centrocercus urophasianus*) hierarchical population units in the western United States. The LCP-MST-4 (least-cost path minimum spanning tree tier 4) did not use any habitat covariates with summary statistics. For LCP-MST-5, cluster levels 9 – 10 identified mean, cluster levels 11 – 12 did not use any covariates with a summary statistic, and cluster-level 13 identified the coefficient of variation (CV). The LCP-MST-4 and -5 were not displayed because they had no competing models.

| **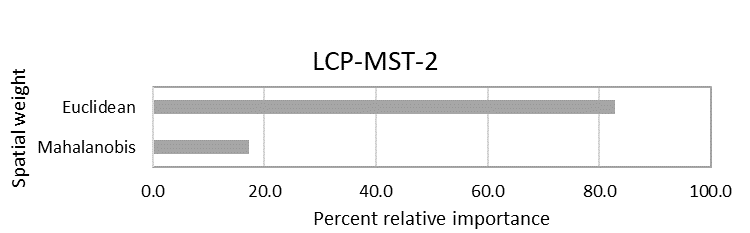** |
| --- |
| **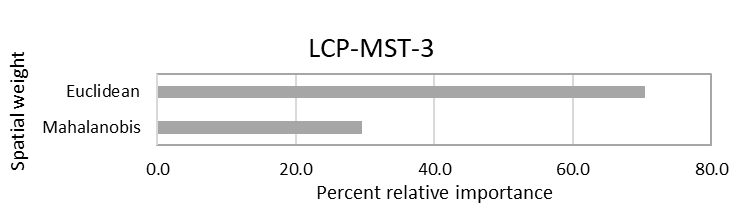** |

**Figure S9**. Relative importance of distance method (Euclidean or Mahalanobis) selected in top model of the Spatial “K”luster Analysis by Tree Edge Removal clustering algorithm for each population structure tier while identifying greater sage-grouse (*Centrocercus urophasianus*) hierarchical population units in the western United States. The LCP-MST-1 (least-cost path minimum spanning tree tier 1) used Euclidean distance method for all subgraphs. The LCP-MST-4 used Mahalanobis distance method for all subgraphs. For LCP-MST-5, cluster levels 9 – 13 used Euclidean distance method. LCP-MST-1, -4, and -5 were not displayed because they had no competing models.

# S4. Evaluation of greater sage-grouse, hierarchical population units

We manually evaluated the results to ensure the grouping of adjacent leks; we found that SKATER sometimes did not enforce adjacency, defined as neighboring leks within the population structure (planar graph network). Figure S10 shows the locations of edits made by *popcluster* and manual ones made after clustering was completed. Cluster levels 8 – 12 included a large cluster in northeast Wyoming and southeast Montana; they were not directly connected in the population structure and therefore incorrectly aggregated by SKATER due to non-adjacency. This modification required corrections to 99 leks for each of these levels. In southern Utah, we delayed clustering due to the effects of an interstate highway believed to affect movements (Technical Team modification). If we had modified the LCP-MST before clustering, these changes would not have been necessary; this accounted for 12 leks in levels 3, 66 in level 5, and 66 in level 6. Table S7 reports all modifications of lek assignment to clusters handled by the code and manually by state. The largest number of changes occurring in Wyoming resulted from an error in the SKATER algorithm, as previously described.

**Table S7**. Description of greater sage-grouse (*Centrocercus urophasianus*) leks (breeding display grounds) changed by software and manually to address occurrences where clustering algorithm (Spatial “K”luster Analysis by Tree Edge Removal) incorrectly resulted in non-hierarchical nesting of clusters (population units). The manual changes will frequently result in propagation of errors because modifications were applied after processing clustering. For the clustering algorithm, we used leks with an “Active” conservation status (n=5,832 leks). Table S8 documents 607 unique leks that were reassigned to different cluster groups to meet our adjacency rules and hierarchical nesting. The percentage of leks changed is relative to leks connected to a population structure tier (i.e., excludes detached leks; see Table S4 for the number of detached leks by cluster level).

|  | **Changed leks** | |  | **Code and manual change** | |
| --- | --- | --- | --- | --- | --- |
| **Cluster level** | **Code change** | **Manual change** |  | **Changed leks** | **Percent change** |
| grsg_cluster_lv1 | 0 | 7 |  | 7 | 0.1 |
| grsg_cluster_lv2 | 5 | 69 |  | 64 | 1.1 |
| grsg_cluster_lv3 | 0 | 111 |  | 111 | 1.9 |
| grsg_cluster_lv4 | 10 | 88 |  | 78 | 1.3 |
| grsg_cluster_lv5 | 1 | 179 |  | 178 | 3.1 |
| grsg_cluster_lv6 | 36 | 142 |  | 106 | 1.8 |
| grsg_cluster_lv7 | 11 | 69 |  | 58 | 1.0 |
| grsg_cluster_lv8 | 90 | 451 |  | 361 | 6.2 |
| grsg_cluster_lv9 | 0 | 324 |  | 324 | 5.6 |
| grsg_cluster_lv10 | 19 | 229 |  | 210 | 3.6 |
| grsg_cluster_lv11 | 0 | 229 |  | 229 | 3.9 |
| grsg_cluster_lv12 | 0 | 109 |  | 109 | 1.9 |
| grsg_cluster_lv13 | 0 | 0 |  | 0 | 0 |
| *Summary* | *172* | *1,835* |  | *2,007* | *34.4* |

**Table S8**. Description of greater sage-grouse (*Centrocercus urophasianus*) lek (breeding display grounds) reassignment to clusters by state after changes made by software and manually to address occurrences where clustering algorithm (Spatial “K”luster Analysis by Tree Edge Removal) incorrectly resulted in non-hierarchical nesting of clusters (population units). There were 607 unique leks reassigned to different cluster groups to meet our adjacency rules and hierarchical nesting.

| **State** | **Number of lek changes** |
| --- | --- |
| CO | 33 |
| ID | 69 |
| MT | 36 |
| NV | 23 |
| OR | 45 |
| UT | 74 |
| WY | 327 |


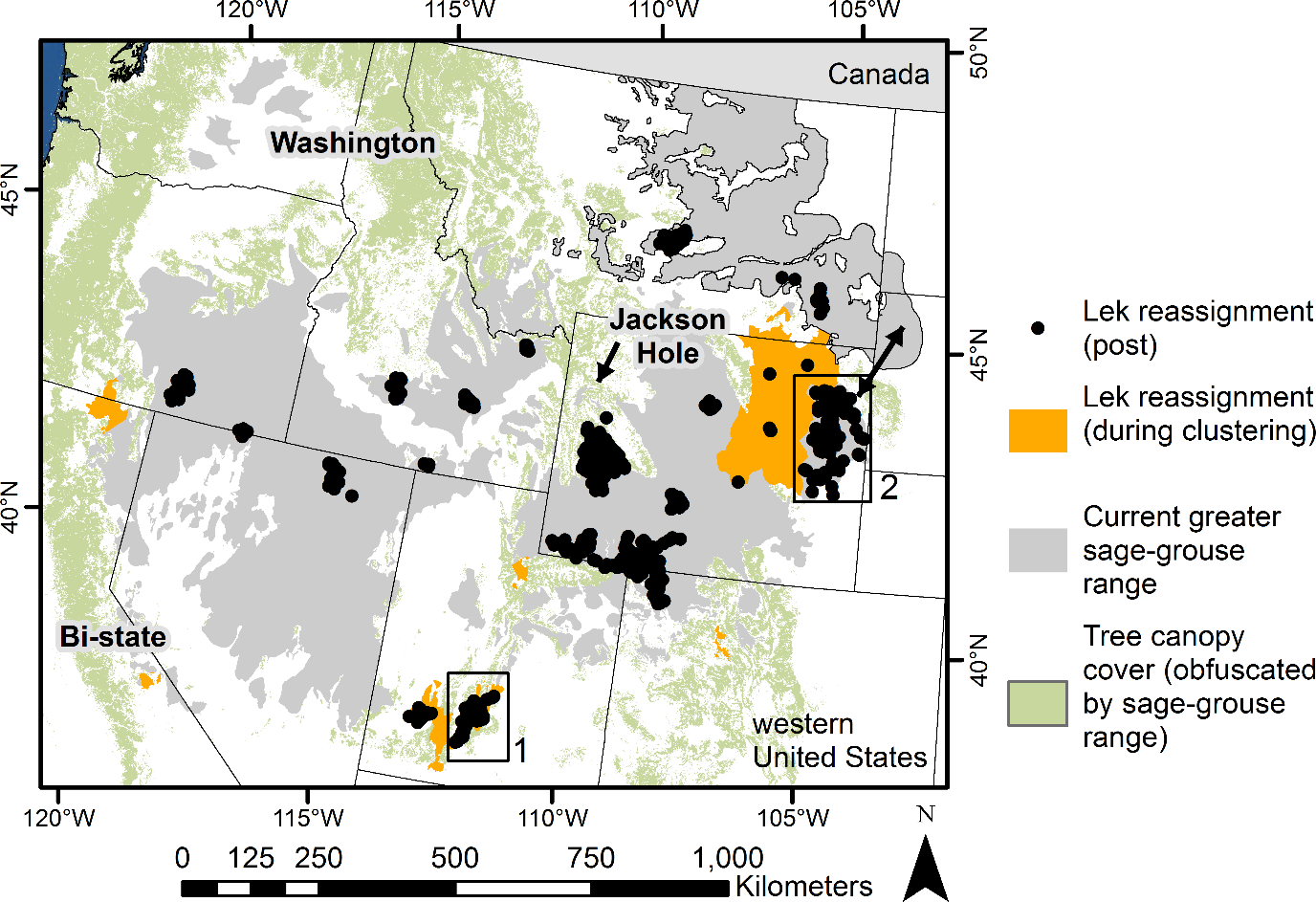


**Figure S10**. Reassignment of greater sage-grouse (*Centrocercus urophasianus*) leks (breeding display grounds) during clustering and post clustering (clustering algorithm: Spatial “K”luster Analysis by Tree Edge Removal [SKATER]) in the western United States. We reassigned 607 unique leks to different cluster groups to meet our adjacency rules and hierarchical nesting using 5,832 active leks. Label 1 denotes reassignment of leks to clusters due to a change suggested by the lead sage-grouse biologist for the Utah Division of Wildlife Resources to the population structure after clustering occurred (not considered an error). Label 2 shows reassignment of leks to clusters where SKATER failed to enforce adjacency using the population structure.

Sage-grouse population units that best minimize the effects of births, deaths, inter-lek movements, immigration, and emigration during a survey period are needed to meet closed population assumptions for modeling (e.g., Iijima, 2020). We worked with numerous state wildlife agency biologists and university and federal researchers to acquire sage-grouse telemetry data (global positioning system [GPS] and very-high frequency [VHF]) to evaluate our sage-grouse population units. Researchers used our software and their data to execute the analyses in some cases. We used the telemetry data to determine how well each cluster level captured an individual bird’s annual home range, providing information about which cluster level best captured closed populations. The home range of individual birds was assigned to a polygon (cluster) where most time (GPS) or locations (VHF) occurred during a biological year (breeding season to breeding season). We describe all available information on movement data in Tables S9 – S15. Figure S10 illustrates the spatial locations of movement studies, and Figures S11 – 12 describe how each cluster level captured closed populations by study area. Figure 7 (main article) shows how each cluster level captured closed populations when considering all location data combined across the species’ range. We did not combine GPS and VHF data for this analysis because the methods differed, and sampling bias was more likely with VHF data.

**Table S9.** Description of Washington greater sage-grouse (*Centrocercus urophasianus*) location data used to evaluate hierarchical population units in the western United States where we grouped locations by biological year (breeding season to breeding season).

| **Data attribute** | **Study area 1** |
| --- | --- |
| Study site location | Washington (Crab Creek and Moses Coulee) |
| Data collection method | Global positioning system |
| Make/model of unit(s) | Platform transmitter terminal |
| Weight of unit | Unknown |
| Temporal frequency | Varies seasonally with 3-7 points per day (3 pts Nov-Feb, 4 pts Mar, 5 pts  Apr, 7 pts May-Aug, 5 pts Sep-Oct) |
| Spatial accuracy threshold | Unknown |
| Data condition (cleaned or raw) | Cleaned by researcher(s) |
| Differentiation of sex | Male only |
| Differentiation of age | Unknown |
| Translocated birds | Crab Creek only |
| Number of birds | 30 |
| Range of years | 2014 – 2019 |
| Median year for biological and breeding (March 1 – June 30) distribution of observations by year | 2017; 2017 |
| Total number of original observations | 48,771 |
| Number of biological (breeding to breeding) and breeding (March 1 – June 30) observations cleaned and clipped to species boundary | 47,978; 15,939 |
| Researcher(s) | Peter J. Olsoy (Washington State University), Daniel Thornton (Washington State University), Michael Schroeder (Washington Department of Fish and Wildlife) |
| Publications associated with data | Unknown |

**Table S10.** Description of Southern Oregon and northern Nevada greater sage-grouse (*Centrocercus urophasianus*) location data used to evaluate hierarchical population units in the western United States where we grouped locations by biological year (breeding season to breeding season).

| **Data attribute** | **Study area 1** | **Study area 2** |
| --- | --- | --- |
| Study site location | Trout Creek southeastern Oregon along Nevada state boundary | Warner Mountains southwestern Oregon along California state boundary |
| Data collection method | Global positioning system | Global positioning system |
| Make/model of unit(s) | Platform transmitter terminal | Platform transmitter terminal |
| Weight of unit | Unknown | Unknown |
| Temporal frequency | Unknown | Unknown |
| Spatial accuracy threshold | Unknown | Unknown |
| Data condition (cleaned or raw) | Cleaned by researcher(s) | Cleaned by researcher(s) |
| Differentiation of sex | Unknown | Unknown |
| Differentiation of age | Unknown | Unknown |
| Translocated birds | Unknown | Unknown |
| Number of birds | 181 | 157 |
| Range of years | 2013 – 2018 | 2015 – 2018 |
| Median year for biological and breeding (March 1 – June 30) distribution of observations by year | 2018; 2018 | 2016; 2016 |
| Total number of original observations | 207,508 | 157,432 |
| Number of biological (breeding to breeding) and breeding (March 1 – June 30) observations cleaned and clipped to species boundary | 206,427; 58,174 | 147,274; 62,161 |
| Researcher(s) | Christopher R. Anthony (Oregon State University), Christian Hagen (Oregon State University) | Christopher R. Anthony, Christian Hagen |
| Publications associated with data | Unknown | Unknown |

**Table S11.** Description of Nevada and California greater sage-grouse (*Centrocercus urophasianus*) location data used to evaluate hierarchical population units in the western United States where we grouped locations by biological year (breeding season to breeding season).

| **Data attribute** | **Study area 1** | **Study area 2** |
| --- | --- | --- |
| Study site location | Nevada and California (including Bi-State) | Bi-state population (Nevada and California state boundary) |
| Data collection method | Global positioning system | Same |
| Make/model of unit(s) | Platform transmitter terminal | Same |
| Weight of unit | Unknown | Same |
| Temporal frequency | Unknown | Same |
| Spatial accuracy threshold | Unknown | Same |
| Data condition (cleaned or raw) | Cleaned by researcher(s) | Same |
| Differentiation of sex | Yes (male [n=174], female [n=257]) | Yes (male [n=59], female [n=52]) |
| Differentiation of age | Yes (adult [n=240], juvenile [n=82], yearling [n=108], and unknown [n=1]) | Yes (adult [n=64], juvenile [n=17], yearling [n=30], and unknown [n=0]) |
| Translocated birds | Unknown | Unknown |
| Number of birds | 431 | 111 |
| Range of years | 2012 – 2020 | Same |
| Median year for biological and breeding (March 1 – June 30) distribution of observations by year | 2013; 2017 | 2015; 2017 |
| Total number of original observations | 592,202 | ~112,640 (some removed when outside sage-grouse range) |
| Number of biological (breeding to breeding) and breeding (March 1 – June 30) observations cleaned and clipped to species boundary | 588,476; 297,514 | 112,640; 59,238 |
| Researcher(s) | Peter Coates (U.S. Geological Survey) | Same |
| Publications associated with data | Unknown | Same |

**Table S12.** Description of Idaho greater sage-grouse (*Centrocercus urophasianus*) location data used to evaluate hierarchical population units in the western United States where we grouped locations by biological year (breeding season to breeding season).

| **Data attribute** | **Study area 1** |
| --- | --- |
| Study site location | Greater sage-grouse range within Idaho |
| Data collection method | Global positioning system |
| Make/model of unit(s) | Platform transmitter terminal |
| Weight of unit | Unknown |
| Temporal frequency | Unknown |
| Spatial accuracy threshold | Unknown |
| Data condition (cleaned or raw) | Cleaned by researcher(s) |
| Differentiation of sex | Yes (male [n=68], female [n=185]) |
| Differentiation of age | Yes (adult [n=110], juvenile [n=72], and yearling [n=23], and unknown [n=48]) |
| Translocated birds | Unknown |
| Number of birds | 325 |
| Range of years | 2012 – 2018 |
| Median year for biological and breeding (March 1 – June 30) distribution of observations by year | 2015; 2015 |
| Total number of observations (uncleaned and cleaned) | 313,696; 308,256 |
| Number of biological (breeding to breeding) and breeding (March 1 – June 30) observations cleaned and clipped to species boundary | 255,564; 160,500 |
| Researcher(s) | Ethan Ellsworth (Bureau of Land Management-Idaho), Vince Guyer (Bureau of Land Management-Idaho), Ann Moser (Idaho Department of Fish and Game), Sara Norman (Idaho Department of Fish and Game), Jordan Rabon (Idaho Department of Fish and Game), Bruce Schoeberl (Idaho Department of Fish and Game), and many field technicians |
| Publications associated with data | Unknown |

**Table S13.** Description of South Dakota greater sage-grouse (*Centrocercus urophasianus*) location data used to evaluate hierarchical population units in the western United States where we grouped locations by biological year (breeding season to breeding season).

| **Data attribute** | **Study area 1** | **Study area 2** |
| --- | --- | --- |
| Study site location | Northwestern South Dakota | Northwestern South Dakota |
| Data collection method | Global positioning system | Very-high frequency |
| Make/model of unit(s) | Platform transmitter terminal | Unknown |
| Weight of unit | Unknown | Unknown |
| Temporal frequency | Unknown | Unknown |
| Spatial accuracy threshold | <=30 m | Unknown |
| Data condition (cleaned or raw) | Cleaned by researcher(s) | Cleaned by researcher(s) |
| Differentiation of sex | Unknown | Unknown |
| Differentiation of age | Unknown | Unknown |
| Translocated birds | Unknown | Tried to not include |
| Number of birds | 8 | 233 |
| Range of years | 2020 | 2006 – 2008; 2016 – 2017 |
| Median year for biological and breeding (March 1 – June 30) distribution of observations by year | 2020; 2020 | 2016; 2007 |
| Total number of original observations | 1,186 | 6,325 |
| Number of biological (breeding to breeding) and breeding (March 1 – June 30) observations cleaned and clipped to species boundary | 1,186; 1,186 | 4,797; 2,204 |
| Researcher(s) | Rebecca E. Newton (Bureau of Land Management), Aaron Johnston (U.S. Geological Survey), Robb Diel (U.S. Geological Survey), Erik Beever | Travis Runia, Chelsea Sink, Jackie Gehrt, Kelsey Norton, Lindsey Bichoff, Shannon Hone (South Dakota Game, Fish and Parks) |
| Publications associated with data | Unknown | Unknown |

**Table S14.** Description of Wyoming greater sage-grouse (*Centrocercus urophasianus*) location data used to evaluate hierarchical population units in the western United States where we grouped locations by biological year (breeding season to breeding season).

| **Data attribute** | **Study area 1** |
| --- | --- |
| Study site location | Bighorn Basin north central Wyoming |
| Data collection method | Global positioning system |
| Make/model of unit(s) | Microwave Telemetry solar Argos/GPS PTTs |
| Weight of unit | 30g |
| Temporal frequency | Winter, Nov 15 – Jan 31: 0900 – 2100 with locations every 3 hours (no Ground Track) 5 locations/day  Spring, Feb 1 – Mar 31: 0600 – 2100 with locations every 3 hours (GT) (6/day)  Summer, Apr 1 – July 31: 0600 – 2200 with locations every 1 hours (GT) (17 /day)  Fall, Aug 1 – Nov 14: 0600 – 2100 with locations every 3 hours (GT) (6/day) |
| Spatial accuracy threshold | Unknown |
| Data condition (cleaned or raw) | Cleaned by researcher(s) |
| Differentiation of sex | All females |
| Differentiation of age | Mixed |
| Translocated birds | None |
| Number of birds | 59 |
| Range of years | 2011 – 2015 |
| Median year for biological and breeding (March 1 – June 30) distribution of observations by year | 2014; 2014 |
| Total number of original observations | 115,002 |
| Number of biological (breeding to breeding) and breeding (March 1 – June 30) observations cleaned and clipped to species boundary | 114,701; 114,701 |
| Researcher(s) | Jimmy Taylor (currently, U.S. Department of Agriculture), Jonathan B. Dinkins (currently, Oregon State University) |
| Publications associated with data | Unknown |

**Table S15.** Description of Colorado greater sage-grouse (*Centrocercus urophasianus*) location data used to evaluate hierarchical population units in the western United States where we grouped locations by biological year (breeding season to breeding season).

| **Data attribute** | **Study area 1** | **Study area 2** |
| --- | --- | --- |
| Study site location | Greater sage-grouse range in northwest Colorado | Greater sage-grouse range in northwest Colorado |
| Data collection method | Global positioning system | Very-high frequency |
| Make/model of unit(s) | Platform transmitter terminal | Unknown |
| Weight of unit | Unknown | Unknown |
| Temporal frequency | Unknown | Unknown |
| Spatial accuracy threshold | Unknown | Unknown |
| Data condition (cleaned or raw) | Cleaned by researcher(s) | Cleaned by researcher(s) |
| Differentiation of sex | Unknown | Unknown |
| Differentiation of age | Unknown | Unknown |
| Translocated birds | Unknown | Unknown |
| Number of birds | 360 | 1037 |
| Range of years | 2009 – 2019 | 2001 – 2014 |
| Median year for biological and breeding (March 1 – June 30) distribution of observations by year | 2010; 2010 | 2004; 2004 |
| Total number of original observations | 329,270 | 27,450 |
| Number of biological (breeding to breeding) and breeding (March 1 – June 30) observations cleaned and clipped to species boundary | 323,837; 108,394 | 26,934; 9,529 |
| Researcher(s) | Anthony D. Apa, Michelle Cowardin, Brian E. Holmes, Liza G. Rossi, and Brett L. Walker (Colorado Parks and Wildlife) | Same |
| Publications associated with data | Unknown | Unknown |


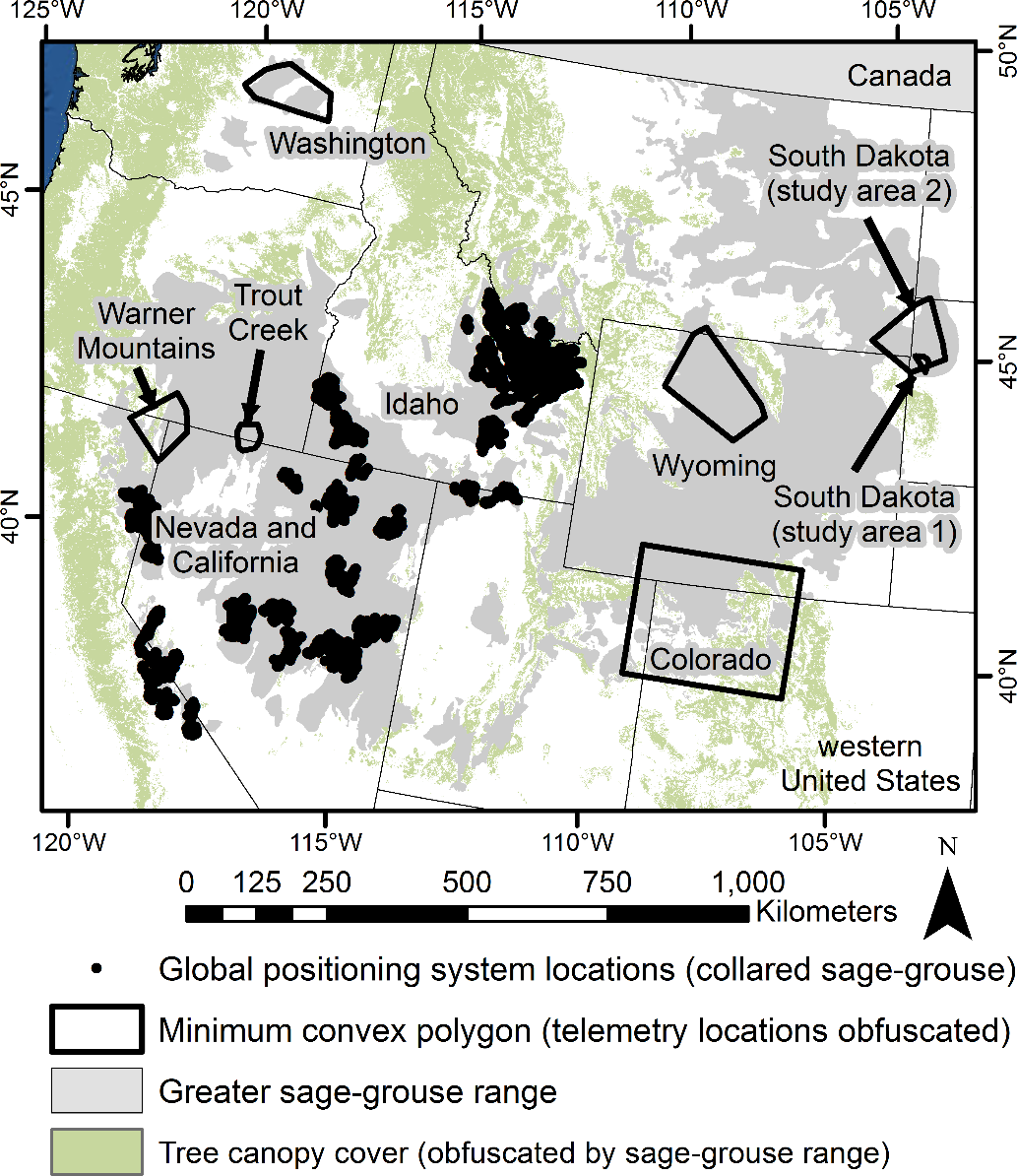


**Figure S11**. Very high frequency (VHF) and global positioning systems (GPS) data were used from multiple independent research studies across the greater sage-grouse (*Centrocercus urophasianus*) range to evaluate hierarchical population units in the western United States. Each study name within the illustration corresponds to data descriptions and data contributors in Tables S9 – S15.

| Mean proportion of time (GPS) or locations (VHF) outside home cluster (±SE) | 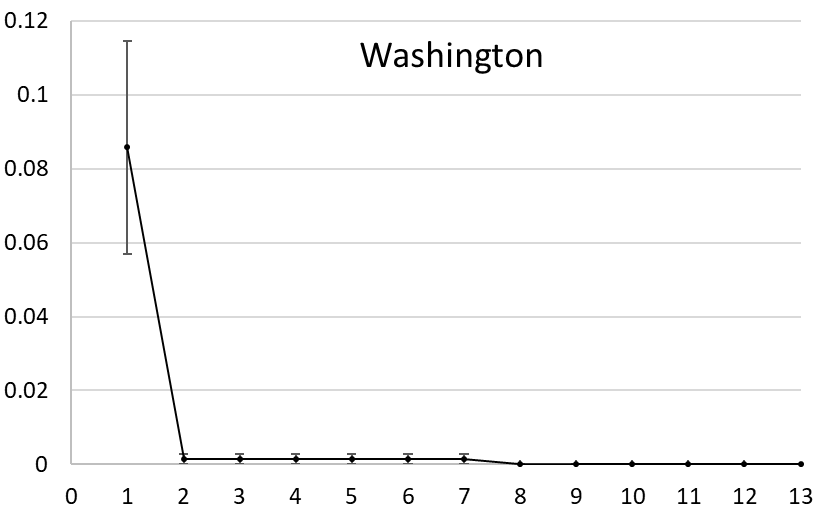 | 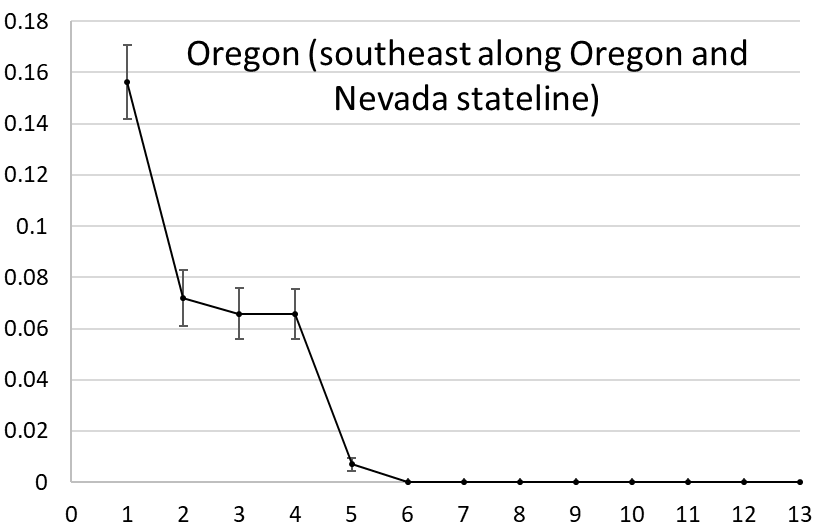 |
| --- | --- | --- |
|  | 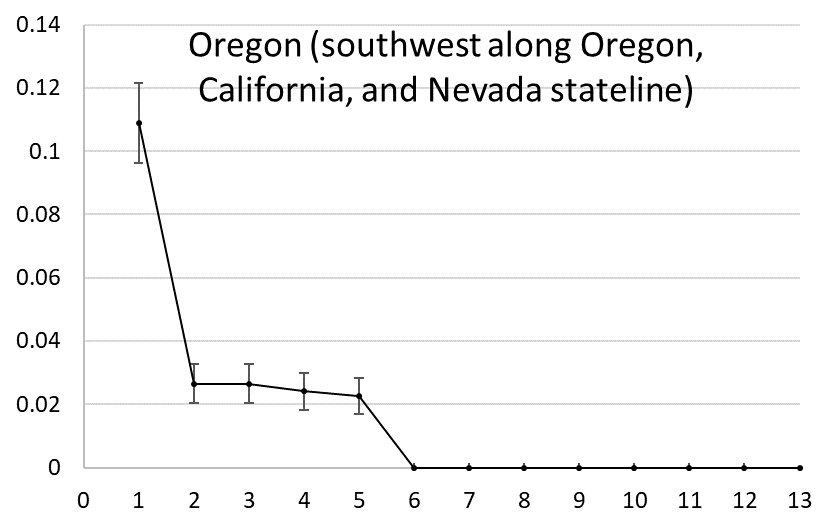 | 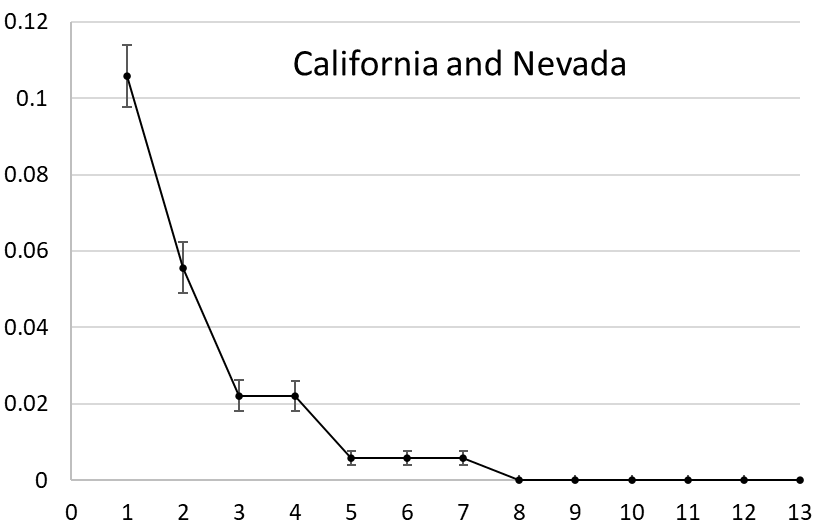 |
|  |  | 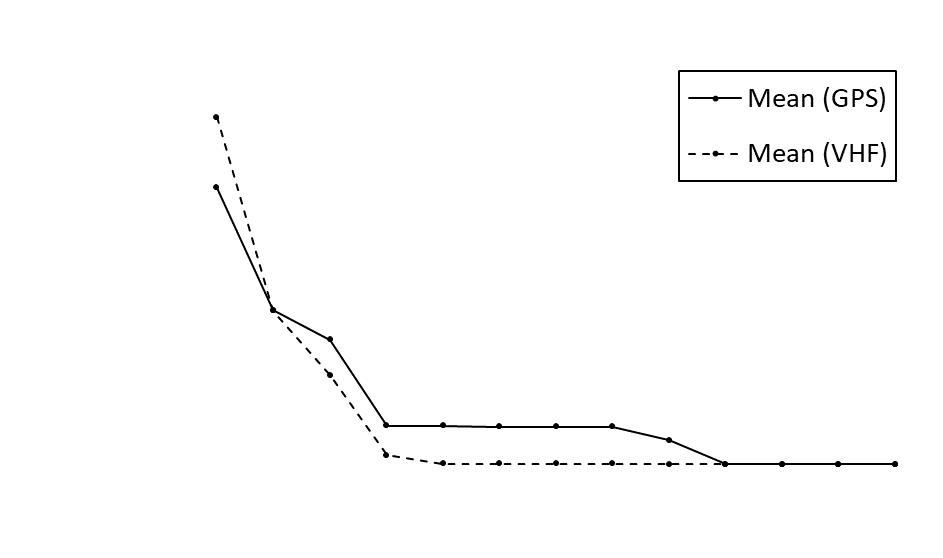 |
|  | Cluster level | |

**Figure S12**. Very high frequency (VHF) and global positioning systems (GPS) data were used from multiple research studies across the greater sage-grouse (*Centrocercus urophasianus*) range to evaluate hierarchical population units in the western United States. The dynamic Brownian bridge movement model (Kranstauber, Kays, Lapoint, Wikelski, & Safi, 2012) and GPS data were used to estimate the time spent outside the home range. We calculated the mean and standard error (SE; whiskers) of time and use birds spent outside their home cluster (polygon) for each cluster level. The Bi-state movement data reflect a genetically isolated population at central Nevada and California state boundaries. See Tables S9 – S11 for contributions and data details.

| Mean proportion of time (GPS) or locations (VHF) outside home cluster (±SE) | 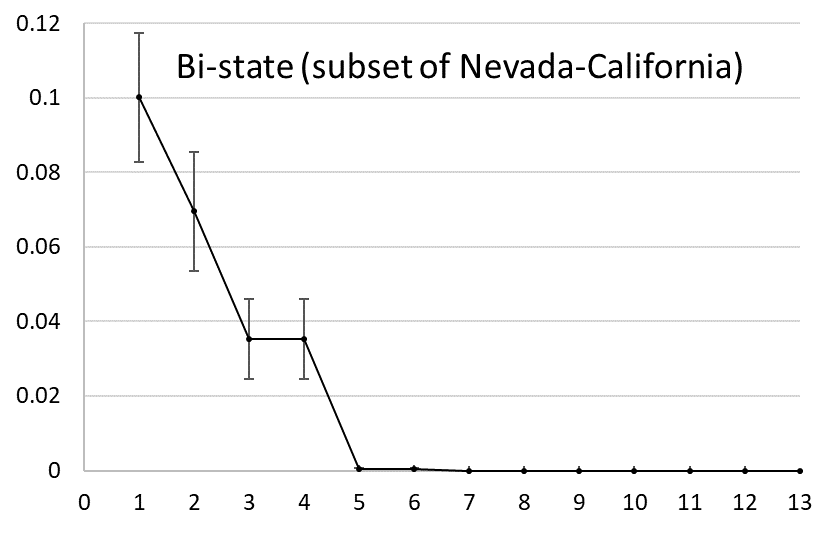 | 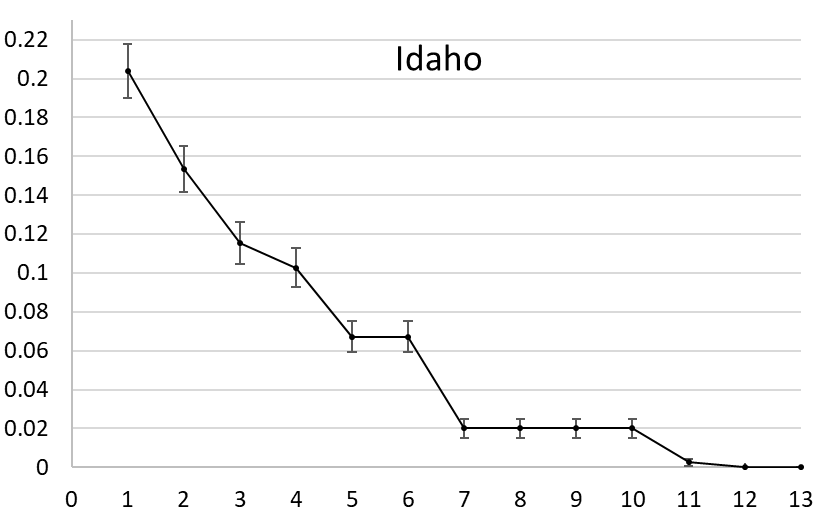 |
| --- | --- | --- |
|  | 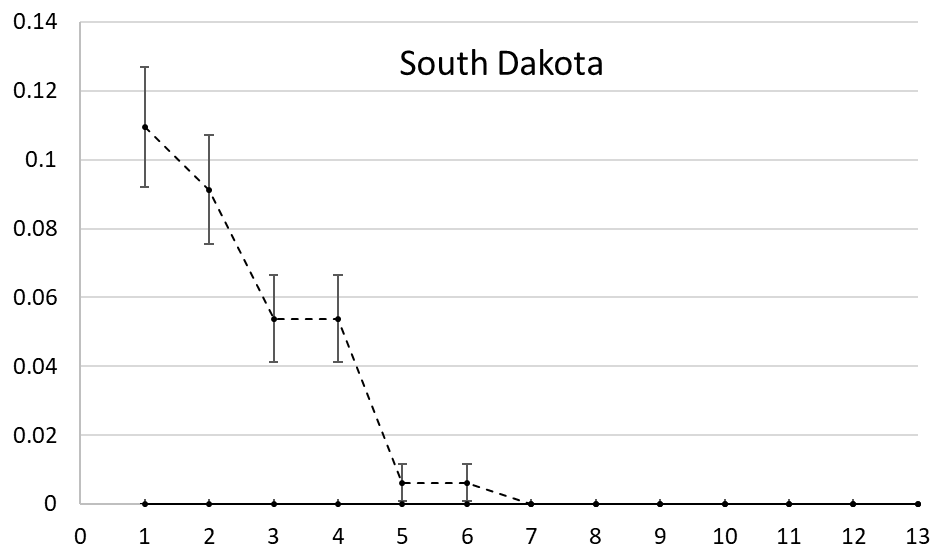 | 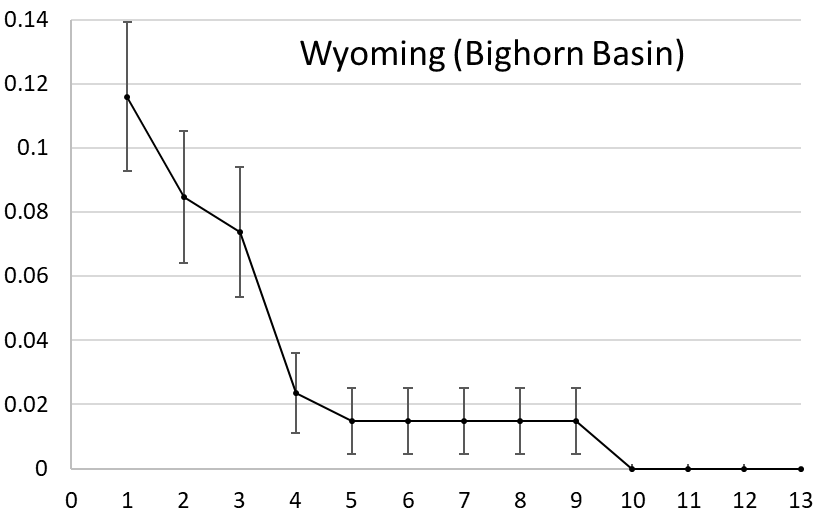 |
|  | 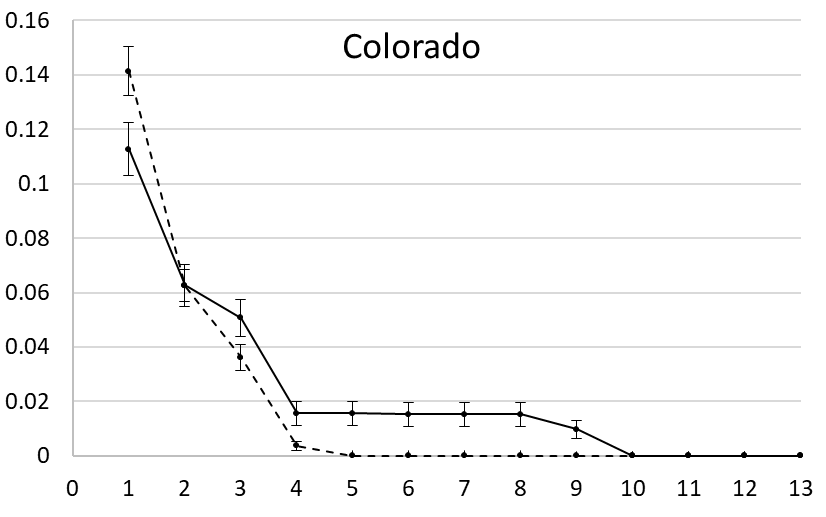 | 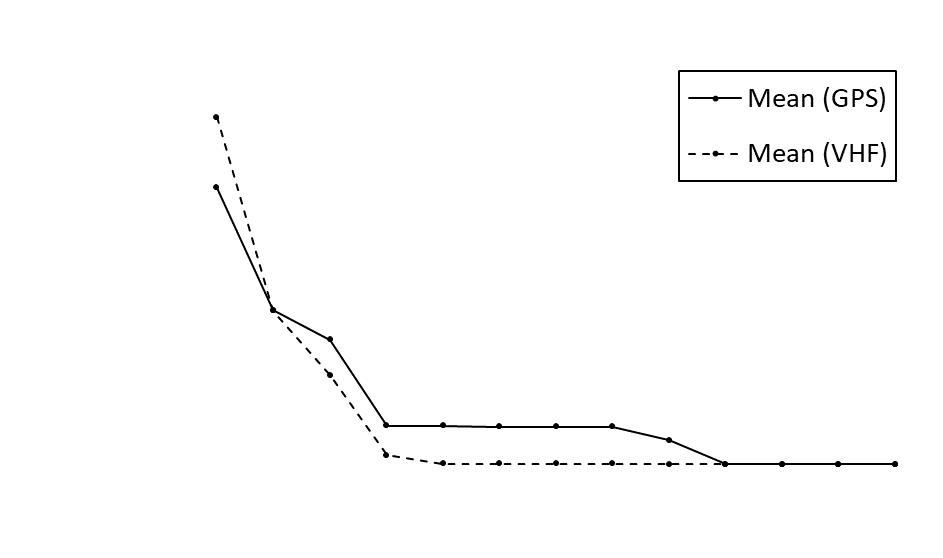 |
|  | Cluster level | |

**Figure S13**. Very high frequency (VHF) and global positioning systems (GPS) data used from multiple research studies across the greater sage-grouse (*Centrocercus urophasianus*) range to evaluate hierarchical population units in the western United States. The dynamic Brownian bridge movement model (Kranstauber et al., 2012) and GPS data were used to estimate the time spent outside the home range. We calculated the mean and standard error (SE; whiskers) of time and use birds spent outside their home cluster (polygon) for each cluster level. The Bi-state movement data reflect a genetically isolated population at central Nevada and California state boundaries. See Tables S11 – S15 for contributions and data details.

# Literature Cited

AssunÇão, R., Krainski, E., Ribeiro, P. J., Jr., & Rodrigues, E. (2012). *Unsupervised classification of geographical units with uncertain attributes applied to vital rates.* Paper presented at the ENIA 2012, 2012, Curitiba. IX Encontro de Inteligência Artificial - ENIA, 2012.

AssunÇão, R. M., Neves, M. C., Câmara, G., & Da Costa Freitas, C. (2006). Efficient regionalization techniques for socio‐economic geographical units using minimum spanning trees. *International Journal of Geographical Information Science, 20*(7), 797-811. doi:10.1080/13658810600665111

Burnham, K. P., Anderson, D. R., & Huyvaert, K. P. (2011). AIC model selection and multimodel inference in behavioral ecology: some background, observations, and comparisons. *Behavioral Ecology and Sociobiology, 65*(1), 23-35. doi:10.1007/s00265-010-1029-6

Gessler, P. E., Moore, I. D., McKenzie, N. J., & Ryan, P. J. (1995). Soil-landscape modelling and spatial prediction of soil attributes. *International Journal of Geographical Information Systems, 9*(4), 421-432. doi:10.1080/02693799508902047

Iijima, H. (2020). A review of wildlife abundance estimation models: Comparison of models for correct application. *Mammal Study, 45*(3), 177-188. doi:10.3106/ms2019-0082

Kranstauber, B., Kays, R., Lapoint, S. D., Wikelski, M., & Safi, K. (2012). A dynamic Brownian bridge movement model to estimate utilization distributions for heterogeneous animal movement. *Journal of Animal Ecology, 81*(4), 738-746. doi:10.1111/j.1365-2656.2012.01955.x

McCune, B., & Keon, D. (2002). Equations for potential annual direct incident radiation and heat load. *Journal of Vegetation Science, 13*(4), 603-606. doi:10.1111/j.1654-1103.2002.tb02087.x

O'Donnell, M. S., Edmunds, D. R., Aldridge, C. L., Heinrichs, J. A., Coates, P. S., Prochazka, B. G., & Hanser, S. E. (2019). Designing hierarchically nested and biologically relevant monitoring frameworks to study populations across scales. *Ecosphere, 10*(9), 1-34. doi:10.1002/ecs2.2872

O'Donnell, M. S., Edmunds, D. R., Aldridge, C. L., Heinrichs, J. A., Monroe, A. P., Coates, P. S., . . . Wiechman, L. A. (2022a). Defining fine‐scaled population structure among continuously distributed populations. *Methods in Ecology and Evolution*, 1-14. doi:10.1111/2041-210x.13949

O'Donnell, M. S., Edmunds, D. R., Aldridge, C. L., Heinrichs, J. A., Monroe, A. P., Coates, P. S., . . . Wiechman, L. A. (2022b). Greater sage-grouse population structure and connectivity data to inform the development of hierarchical population units (western United States). In: U.S. Geological Survey Data Release.

O'Donnell, M. S., Edmunds, D. R., Aldridge, C. L., Heinrichs, J. A., Monroe, A. P., Coates, P. S., . . . Wightman, C. S. (2021). Synthesizing and analyzing long-term monitoring data: A greater sage-grouse case study. *Ecological Informatics, 63*, 1-16. doi:10.1016/j.ecoinf.2021.101327

O'Donnell, M. S., & Ignizio, D. A. (2012). Bioclimatic predictors for supporting ecological applications in the conterminous United States. In (pp. 10). Reston, Virginia, USA: U.S. Geological Survey Data Series 691, 10 p.

Prism Climate Group. (2015). *Precipitation and temperature climate normals (1981 – 2010)*.

Rigge, M., Homer, C., Cleeves, L., Meyer, D. K., Bunde, B., Shi, H., . . . Bobo, M. (2020). Quantifying western U.S. rangelands as fractional components with multi-resolution remote sensing and in situ data. *Remote Sensing, 12*(3), 412-412. doi:10.3390/rs12030412

Sappington, J. M., Longshore, K. M., & Thompson, D. B. (2007). Quantifying landscape ruggedness for animal habitat analysis: a case study using bighorn sheep in the Mojave Desert. *The Journal of Wildlife Management, 71*(5), 1419-1426. doi:10.2193/2005-723

Soille, P. (2004). Optimal removal of spurious pits in grid digital elevation models. *Water Resources Research, 40*(12), 1-9. doi:10.1029/2004WR003060

Tarboton, D., Bras, R., & Rodriguez-Iturbe, I. (1991). On the extraction of channel networks from digital elevation data. *Hydrological Processes, 5*(1), 81-100. doi:10.1002/hyp.3360050107

U.S. Fish and Wildlife Service. (2014a). Greater sage-grouse 2015 USFWS status review management zones. In: U.S. Fish and Wildlife Service, <https://www.sciencebase.gov/catalog/item/56f96b30e4b0a6037df06216>, accessed 24 June 2021.

U.S. Fish and Wildlife Service. (2014b). Greater sage-grouse 2015 USFWS status review priority areas for conservation (PACs). In: U.S. Fish and Wildlife Service, <https://www.sciencebase.gov/catalog/item/56f96d88e4b0a6037df066a3>, accessed 24 June 2021.

U.S. Fish and Wildlife Service. (2015). Greater sage-grouse draft current range. In: U.S. Fish and Wildlife Service. Denver, Colorado, USA. <https://www.sciencebase.gov/catalog/item/56f96693e4b0a6037df06034>. Accessed 8 Sept, 2015.

U.S. Geological Survey. (2018). *1/3rd arc-second digital elevation models (DEMs) - USGS National Map 3DEP downloadable data collection*.

U.S. Geological Survey, & Homer, C. G. (2019). *National Land Cover Database (NLCD) 2016 Shrubland Fractional Components for the Western U.S. (ver. 1.0, May 15 2019), U.S. Geological Survey data release, accessed 3/28/2019 (available from author); v2:* [*https://doi.org/10.5066/P9LTU2QM*](https://doi.org/10.5066/P9LTU2QM).

Weiss, A. D. (2001). *Topographic position and landforms analysis*. Retrieved from San Diego, CA: <http://www.jennessent.com/downloads/tpi-poster-tnc_18x22.pdf>

Xian, G., Homer, C., Rigge, M., Shi, H., & Meyer, D. (2015). Characterization of shrubland ecosystem components as continuous fields in the northwest United States. *Remote Sensing of Environment, 168*, 286-300. doi:10.1016/j.rse.2015.07.014
